# Comprehensive analysis of omics data identifies relevant gene networks for Attention-Deficit/Hyperactivity Disorder (ADHD)

**DOI:** 10.1038/s41398-022-02182-8

**Published:** 2022-09-24

**Authors:** Judit Cabana-Domínguez, María Soler Artigas, Lorena Arribas, Silvia Alemany, Laura Vilar-Ribó, Natalia Llonga, Christian Fadeuilhe, Montse Corrales, Vanesa Richarte, Josep Antoni Ramos-Quiroga, Marta Ribasés

**Affiliations:** 1 Psychiatric Genetics Unit, Group of Psychiatry, Mental Health and Addiction, Vall d’Hebron Research Institute (VHIR), Universitat Autònoma de Barcelona, Barcelona, Spain; 2 Department of Mental Health, Hospital Universitari Vall d’Hebron, Barcelona, Spain; 3 Biomedical Network Research Centre on Mental Health (CIBERSAM), Madrid, Spain; 4 Department of Genetics, Microbiology, and Statistics, Faculty of Biology, Universitat de Barcelona, Barcelona, Spain; 5 Department of Psychiatry and Forensic Medicine, Universitat Autònoma de Barcelona, Barcelona, Spain

**Keywords:** ADHD, Genomics

## Abstract

Attention-deficit/hyperactivity disorder (ADHD) is a highly prevalent neurodevelopmental disorder that results from the interaction of both genetic and environmental risk factors. Genome-wide association studies have started to identify multiple genetic risk loci associated with ADHD, however, the exact causal genes and biological mechanisms remain largely unknown. We performed a multi-step analysis to identify and characterize modules of co-expressed genes associated with ADHD using data from peripheral blood mononuclear cells of 270 ADHD cases and 279 controls. We identified seven ADHD-associated modules of co-expressed genes, some of them enriched in both genetic and epigenetic signatures for ADHD and in biological pathways relevant for psychiatric disorders, such as the regulation of gene expression, epigenetics and immune system. In addition, for some of the modules, we found evidence of potential regulatory mechanisms, including microRNAs and common genetic variants. In conclusion, our results point to promising genes and pathways for ADHD, supporting the use of peripheral blood to assess gene expression signatures in psychiatric disorders. Furthermore, they highlight that the combination of multi-omics signals provides deeper and broader insights into the biological mechanisms underlying ADHD.

## Introduction

Attention-deficit/hyperactivity disorder (ADHD) is a highly prevalent neurodevelopmental disorder that affects around 5–6% of children and adolescents worldwide, and in 40–65% of cases persist into adulthood [1]. It is mainly characterized by inattention and/or hyperactivity and high levels of impulsivity.

ADHD is a complex disorder that results from the interaction of both genetic and environmental risk factors, with an estimated heritability of 70–80% throughout the lifespan [2]. Several studies support the role of both common and rare genetic variants in the development of ADHD, although its etiology and pathogenesis still remain largely unknown [2]. The first genome-wide association study (GWAS) meta-analysis identifying genetic risk variants for ADHD (20,183 cases and 35,191 controls) was published in 2019 [3]. They identified 12 independent ADHD risk loci and estimated that common variants account for 22% of the total ADHD heritability. In addition, very recently, a larger GWAS meta-analysis on ADHD reported 21 new loci and a reduced estimated SNP heritability (*h*^2^_SNP_ = 14%) [4]. These data highlight that part of the genetic variance still needs to be explained, which may be accounted, in part, for gene by environment interactions [5]. Epigenetic processes (i.e. histone modifications, DNA methylation and microRNAs) are potential mechanisms by which environmental risk factors lead to changes on gene expression and long-lasting alterations in the neuronal circuits found in psychiatric disorders like ADHD [6]. Recently, the first epigenome-wide association study (EWAS) in peripheral blood mononuclear cells (PBMCs) from adults with ADHD was published, identifying four regions differentially methylated and located in genes previously related to autoimmune disorders, cancer, or neuroticism [7]. Additional EWAS in saliva and whole blood have been performed both in adults and children with ADHD diagnosis or ADHD symptoms, however, results among studies are not consistent and further studies with larger sample sizes are needed [8–12].

Although genetic and epigenetic factors that contribute to the etiology of ADHD have started to be identified through GWAS and EWAS, their biological relevance is difficult to characterize, in part, because genetic risk loci were usually associated with the nearest gene, which may not be necessarily the true causal one. In contrast, the analysis of gene expression profiles provides a closer physiological picture of the disorder that is easier to interpret, and reduces the burden of multiple testing. Transcriptome studies in ADHD, nevertheless, are limited by the inaccessibility of brain samples and have been focused on whole blood or PBMCs. To date, eight transcriptomic studies on ADHD have been performed, highlighting alterations in genes involved in several neuronal functions and the immune system [13–20]. However, the studies performed so far were based on differential gene expression analyses between ADHD cases and controls, which assume that every gene acts as an independent unit in the expression landscape and select genes based on statistical significance. In contrast, gene co-expression network analyses use an unsupervised framework to identify groups of genes with similar expression patterns (co-expressed genes) independently of any phenotype and then correlate these gene modules with a phenotype of interest. This approach has been widely used to characterize patterns of co-expression in normal brain [21, 22] and both in brain and blood samples from several psychiatric disorders [23–28].

In the present study, we aimed to perform a multi-step analysis to identify and characterize modules of co-expressed genes associated with ADHD using expression data from PBMCs of ADHD cases and controls. To further understand the biological relevance and provide a more accurate picture of the regulatory mechanisms, we performed a comprehensive characterization of genes in each module and combined genomic and transcriptomic data to identify loci that may regulate the ADHD-associated co-expressed genes.

## Materials and methods

### Study design

**Fig. 1:**
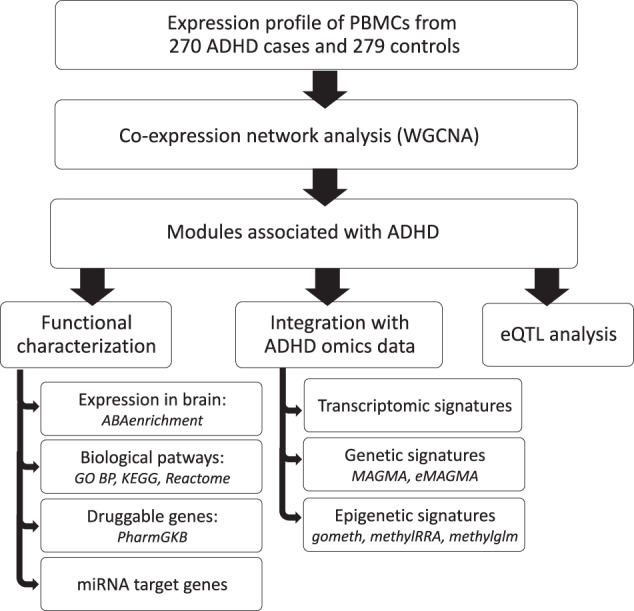
Flowchart of the study. Modules of co-expressed genes were identified from peripheral blood mononuclear cells (PBMCs) of processed transcriptomic data from 270 ADHD cases and 279 controls by using Weighted Gene Correlation Network Analysis (WGCNA). Then, we assessed the association of the resulting modules with the ADHD status and investigated their biologically relevance by (i) performing enrichment analyses in brain expression (*ABAenrichment* R package), functional pathways, druggable genes and miRNA target genes using WebGestAlt webtool; (ii) integrating ADHD transcriptomic, genetic and epigenetic data from GWAS meta-analysis [3] and EWAS [7] on ADHD; and (iii) running a co-expression module eQTL analysis to identify loci regulating the ADHD-associated modules of co-expressed genes.

A comprehensive and multi-step approach was applied to identify and characterize modules of co-expressed genes in PBMCs. In the first step we ran a Weighted Gene Correlation Network Analysis (WGCNA) on the processed transcriptomic data from 270 ADHD cases and 279 controls and assessed the association of the resulting co-expression modules with ADHD status. Subsequently, we disentangled the biological relevance of the ADHD-associated co-expression modules by (i) performing enrichment analyses in brain expression, functional pathways, druggable genes and miRNA target genes, (ii) combining results with ADHD genetic, transcriptomic, and epigenetic signatures, and (iii) running a co-expression module eQTL analysis to identify loci regulating the ADHD-associated modules of co-expressed genes (Fig. 1).

### Participants

Analysis of co-expression modules was performed in an in-house sample of 270 ADHD cases (59.3% male, mean age = 34.2 years, s.d = 11.7) and 279 controls (56.9% male, mean age = 36.6 years, s.d = 9.9). All subjects were of European ancestry. Clinical assessment was conducted by structured interviews and self-reported questionnaires as previously described [19]. Detailed information is available in Supplementary Information. The study was approved by the Clinical Research Ethics Committee (CREC) of Hospital Universitari Vall d’Hebron, methods were performed in accordance with the relevant guidelines and regulations and written informed consent was obtained from all subjects before inclusion in the study.

### Transcriptome profiling and weighted gene correlation network analysis (WGCNA)

RNA from PBMCs was isolated, hybridized to GeneChip Human Gene 1.1 ST 96-Array plate (Affymetrix) and data were analyzed as previously described [19] (Supplementary Information). Modules of co-expressed genes were identified from processed transcriptomic data by the *WGCNA* R-package [29]. A soft-thresholding power of 4 was selected (Fig. S1) and one-step network construction and module detection was performed considering an unsigned network type with default values (additional details in Supplementary Information). Gene expression for each module was represented by a module eigengene, derived from its first principal component and treated as a quantitative trait in the downstream analyses. The association between the module eigengenes and ADHD status or potential confounding factors (age, sex, RNA integrity number (RIN), and batch) was tested using regression analyses. Bonferroni correction was applied to correct for multiple testing considering the overall number of co-expression modules constructed (*P* < 0.05/27 modules <1E-03).

Within each module we examined the correlation between module membership (an indicator of the intramodular connectivity of a gene based on the association between its expression and the module eigengene) and gene significance (effect size of the association between each gene and ADHD) using Pearson correlation (Fig. S2).

### Enrichment analyses in the ADHD-associated co-expression modules

We assessed whether genes in each ADHD-associated co-expression module were expressed in specific brain regions at different developmental stages using data from the Allen Human Brain Atlas with *ABAEnrichment* R-package [30] (additional details in Supplementary Information). Then, enrichment analyses with the webtool WebGestAlt (WEB-based GEne SeT AnaLysis Toolkit, http://www.webgestalt.org/) [31] were performed on: (i) Gene Ontology non-redundant Biological Process (GO), Kyoto Encyclopedia of Genes and Genomes (KEGG) and Reactome, (ii) target genes of pharmacological drugs based on the information from PharmGKB and (iii) miRNA target genes. False discovery rate *P*-value (*P*_FDR_) < 0.05 was set as the significance threshold.

In addition, the correlation between the identified miRNA and its corresponding module eigengene was tested using the non-parametric Spearman rank correlation test in a subset of 310 individuals included in the WGCNA (60% overlap; 150 ADHD cases and 160 controls) from whom miRNA expression profile data from PBMC were available as described in Sanchez-Mora et al. [16]. Expression was available and retrieved from a total of 27 mature miRNAs and Bonferroni correction was used to adjust for multiple testing (*P* < 0.05/27 tests <1.85E−03).

### Integrative analysis of ADHD-associated co-expression modules and ADHD omics data

#### ADHD transcriptomic signatures

After quality control and sample processing, differential gene expression profiles between the 270 ADHD cases and 279 controls used in the WGCNA analysis were obtained with *Limma* R-package [32]. Only genes with *P*_FDR_ < 0.05 and fold change (FC) > |1.15| were considered differentially expressed and were used to test for enrichment in the ADHD-associated co-expression modules using a F-Fisher test and Bonferroni correction across all modules (*P* < 0.05/7 modules <7.1E−03).

#### ADHD genetic signatures

The identified ADHD-associated co-expression modules were used as gene sets to test for enrichment in ADHD genetic signatures, considering the European ancestry GWAS summary statistics on ADHD described by Demontis et al. [3]. Gene-based analyses were run in MAGMA_v1.08 [33] using the SNP-wise mean model, and SNPs were assigned to genes based on a positional-approach and eMAGMA [34, 35]. Competitive gene-set analysis was performed using *P*-values obtained from each gene-based analysis. Bonferroni correction was applied to correct for multiple testing (*P* < 7.1E-03; Supplementary Information)

#### ADHD methylation signatures

To test for enrichment in ADHD epigenetic marks in modules of co-expressed genes we used the summary statistics of an EWAS on PBMCs from 103 ADHD patients and 100 controls [7] (90% sample overlap with the in-house sample used in WGCNA), setting the unadjusted *P*-value < 0.01 to select differentially methylated proves (*n* = 3967 CpG sites). We considered enrichment in epigenetic signatures in a co-expression module when the three approaches used (*methylglm, methylRRA* and *gsameth* [36]) were significant after applying the Bonferroni correction for multiple comparison corrections (*P* < 7.1E−03; Supplementary Information).

### Gene-module eQTL analysis and functional annotation

Genetic information was available from a subset of 231 ADHD subjects and 264 controls included in the WGCNA. A GWAS with each module eigengene as the dependent variable were performed to identify genetic variants associated with each co-expression module. After ascertain normality of module eigengene (Table S1), seven gene-module eQTL analyses were run under an additive linear regression model using PLINK_1.09, adding as covariates the first 10 principal components, sex, age, and the genotyping wave. Lead SNPs were identified in each eQTL analysis considering a *P*-value < 1E−06 and functionally annotated using the FUMA protocol (Functional Mapping and Annotation of Genome-Wide Association Studies, https://fuma.ctglab.nl/) [37] (Supplementary Information).

Raw data from this article are not publicly available because of limitations in ethical approvals and the summary data will be available upon request.

## Results

The WGCNA identified a total of 27 modules of co-expressed genes with size ranging from 33 to 2191 genes (Fig. S1). 42.7% of genes (*N* = 8114) were not assigned to any module and remained in the module M0. Seven co-expression modules were associated with ADHD after multiple testing correction (modules M1–M7, Table S2). No association between module eigengenes and potential confounders, including age, sex, RIN, or batch, was detected for any module (Table S2). All modules were consistent across samples and have characteristic band structures suggestive of well-defined modules (Fig. S2). Interestingly, modules M1, M3, and M6 showed high module membership—gene significance correlation (*r*^2^ > 0.4), suggesting that the higher the connectivity of a gene within the module, the stronger the association with ADHD (Fig. S3).

Different patterns of gene expression in the brain at different developmental stages were found across ADHD-associated co-expression modules. M2 genes are broadly expressed in the whole brain during the lifespan, while genes in M7 are expressed in a specific brain area, the M1C_primary motor cortex, only during the prenatal stage. Besides, genes in modules M1 and M4 show broader expression in different areas from the telencephalon during the prenatal period and are mainly expressed in the cerebellar cortex after birth (Table 1 and S3).

**Table 1:** Summary of the main results from the analyses on the ADHD-associated modules of co-expressed genes.

Module	No. genes	Association with ADHD^a^	Brain expression	Main biological function	ATC1 drug categories	miRNA target genes	ADHD transcriptomic signature	ADHD genetic signature^b^	ADHD epigenetic signature^c^
**M1**	1546	Effect= 9.53 *P* = 9.3E−06	Prenatal—telencephalon Postnatal—cerebellum	Posttranscriptional regulation of gene expression Methylation	A, B, C, L	40 miRNAs (24 miR families)	–	–	–
**M2**	1239	Effect = −6.49 *P* = 1.7E−03	Whole brain	mRNA processing Metabolism	A, B, C, D, J, L	–	–	*–*	***mglm*** = **5.2E−03**
**M3**	63	Effect= 7.87 *P* = 1.8E−03	–	–	–	–	–	–	*gsam* = 0.0269 *mglm* = 7.9E−03
**M4**	885	Effect = −7.82 *P* = 1.8E−03	Prenatal - telencephalon Postnatal - cerebellum	Regulation of gene expression Covalent chromatin modification	B, L	-	-	***MA*** = **1.8E−03** ***eM*** = **4.2E−03**	***gsam*** = **1.6E−04** ***mRRA*** = **1.1E−03** ***mglm*** = **8.1E−05**
**M5**	133	Effect = −11.17 *P* = 2.6E-06	−	Immune response	A, B, C, D, H, J, L, M, N, R, S	−	***P*** = **2.2E−16**	−	−
**M6**	258	Effect= 11.58 *P* = 2.1E−07	–	mRNA processing	J	−	−	−	−
**M7**	185	Effect= 8.66 *P* = 4.4E-05	Prenatal - M1C	Posttranscriptional regulation of gene expression Epigenetics	B	5 miRNAs	-	-	*gsam* = 0.0157

In bold results from gene-set analyses that overcome multiple testing corrections for each method.

ATC1 (Anatomical Therapeutic Chemical) categories: A = Alimentary system and metabolism; B = Blood and hematopoietic organs; C = Heart therapy; D = Dermatics; H = Hormone preparations excl.sex; J = Antiinfective for systemic use; L = Antineoplastic and immunomodulating agents; M = musculo-skeletal system; N = Nervous system; R = Respiratory tract; S = Sensory organs. MA = MAGMA; eM = eMAGMA; mglm = methlyglm; gsam = gsameth; mRRA = methylRRA; DE = differentially expressed; M1C = human primary motor cortex.

^a^Logistic regression Effect = log(OR).

^b^Demontis et al. Nat Genet. 2019;51(1):63–75.

^c^Rovira et al. Transl Psychiatry. 2020;10(1):199.

To explore the biological relevance of ADHD-associated co-expression modules further, we performed a functional enrichment analysis in genes in each module and found that several of them were enriched in genes involved in pathways previously related to psychiatric disorders [38], including the *posttranscriptional regulation of gene expression* and *epigenetics* (M1 and M7), *covalent chromatin remodeling* (M4) or *immune system* and *inflammatory response* (M5), among others (Table 1 and S4-6). We also performed an enrichment analysis in druggable genes in the ADHD-associated co-expression modules. For six out of the seven modules we identified enrichment in target genes of at least one drug, being *Antiinfective for systemic use* and *Antineoplastic and immunomodulating agents* the most common Anatomical Therapeutic Chemical classification categories across all modules (Table 1 and S7-8). Interestingly, module M5, enriched in genes involved in the immune system and inflammatory response, showed enrichment in drugs from all Anatomical Therapeutic Chemical categories, especially those related to the immune response, as expected.

Enrichment in miRNA target genes was identified in modules M1 and M7. Genes in M1 were targeted by 24 families of miRNAs, resulting on 40 mature miRNAs, and genes in M7 were targeted by five mature miRNAs (Table 1 and S9). Consistently, a significant correlation between the eigengene profile of module M1 and the expression of four out of 27 of these miRNAs (hsa-miR-142–5p, hsa-miR-181a-5p, hsa-miR-192–5p, and hsa-miR-215–5p) was found in a subset of 310 individuals (150 ADHD cases and 160 controls) from which miRNA and gene expression from PBMCs was available (Fig. S4 and Table S10).

Then, we further explored the ADHD-associated co-expression modules by integrating transcriptomics with genetic and epigenetic data on ADHD. We explored whether genes differentially expressed between ADHD cases and controls were grouped in any of the identified ADHD-associated modules of co-expressed genes, and found a significant enrichment in module M5 (*P* < 2.2E−16), which also remained significant when considering only highly connected genes (module membership > 0.8; Table 1). In addition, we found module M4 significantly enriched in genetics (*P*_MAGMA_ = 1.8E−03; *P*_eMAGMA_ = 4.2E−03) and epigenetics (*P*_gsameth_ = 1.6E−04; *P*_methylRRA_ = 1.1E−03; *P*_methylglm_ = 8.1E−05) signatures for ADHD, using data from GWAS meta-analysis [3] and EWAS [7] on ADHD (Table 1 and S11).

**Fig. 2:**
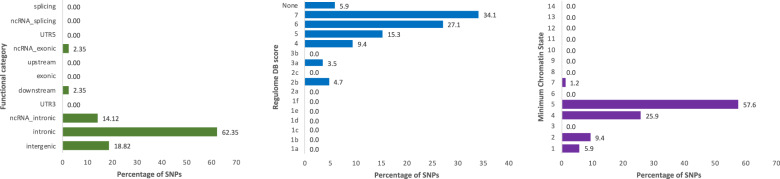
Functional categories, Regulome DB scores, and minimum chromatin states for independent risk loci associated to any module eigengene. Regulome DB score predicts likelihood of regulatory functionality, lower scores indicate higher likelihood. Further information can be found in Boyle et al. [68]. Minimum Chromatin State across 127 tissue and cell types, lower scores indicate higher accessibility, with states 1–7 referring to open chromatin states.

**Fig. 3:**
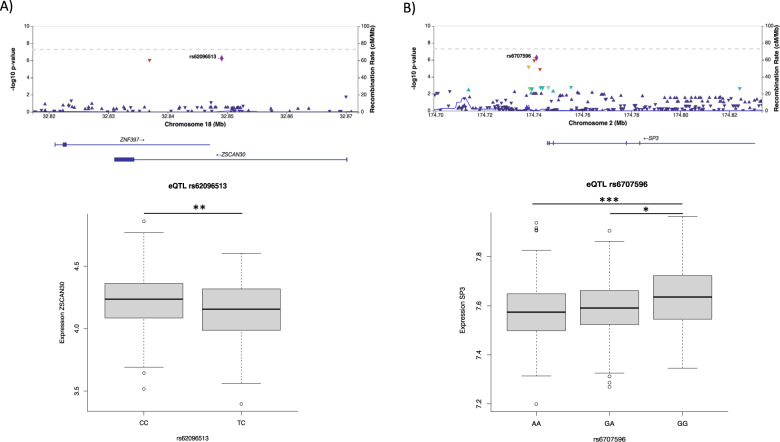
Regional plots and cis-eQTL analyses in PBMCs in our in-house sample of 270 ADHD cases and 279 controls. In the top figure, regional plots for module eigengenes for the (**A**) rs62096513 and (**B**) rs6707596 loci and in the bottom figure, boxplots showing the effect of rs62096513 in *ZSCAN30* and rs6707596 in *SP3* gene expression. Results from the linear regression *p*-value are shown: *P < 0.5, **P < 0.01, ***P < 0.001.

We performed a co-expression module eQTL analysis to identify loci regulating ADHD-associated modules in a subset of 495 individuals included in the WGCNA (91.3%) from whom genomic and gene expression profiles were available. After strict quality control criteria, we ran a GWAS on module eigengenes of each of the seven ADHD-associated co-expression modules independently (M1–M7; Fig. S5). QQ plots indicate minimal effects of genomic inflation, and consequently population substructure, on the analyses (Fig. S6). No SNP overcame the genome-wide significance threshold, but 12 independent genomic loci showed suggestive evidence of association (*P* < 1E−06) with different module eigengenes (Table 2 and Fig. S7). Functional annotations revealed that these loci lay on regions of open chromatin and that most of the signals were intergenic or intronic (Fig. 2). Several SNPs in these genomic risk loci were likely to affect the binding of transcription factors (RBD score = 2b; rs73866266, rs59928606, rs10830974 and rs36098630), had CADD scores > 12.37, suggesting high deleteriousness (rs73170573, rs13408514, rs1508617, rs9565360, and rs10830974; Fig. 2, Table 2 and S12) or were located in regulatory regions of the brain (rs73170578, rs62096513, and rs12583109), according to the information of enhancer and promotor histone marks from the HaploReg webtool [39] (Table 2). In addition, four SNPs, rs62096513, rs6707596, rs66506812 and rs2462337, lay in nearby genes encoding transcription factors (*ZSCAN30*, *SP3*, *CSRNP3* and *CUX1*, respectively; Table 2). Of them, rs6707596 nearby *SP3* and rs62096513 located in intron 1 of *ZSCAN30* were cis-eQTL of these genes in PBMCs in our sample (Fig. 3). Interestingly, the co-expression module M1, which showed suggestive evidence of association with rs62096513 that lies in blood and brain regulatory regions of *ZSCAN30* and is cis-eQTL in PBMCs, is enriched in target genes for this specific transcription factor (P = 1.27E-07), which suggest that *ZSCAN30* may be upstream regulator of the M1 module of co-expressed genes.

**Table 2:** Top hits from GWAS on the module eigengenes from the seven ADHD-associated co-expression modules.

Module	SNP	GWAS P-value	MAF	intra/intergenic	Nearby genes	TF^a^	Enrichment on target genes^b^	Regulatory region in^c^		eQTL in PBMCs^d^	eQTL whole blood^e^
								blood	brain		
M1	rs73170578	2.17E−07	0.061	intra	*CNTNAP2*	–	–	–	SNP in LD (rs73170573)	–	–
M1	rs62096513	5.40E−07	0.066	intra	*ZSCAN30*	YES	YES	YES	YES	YES	*–*
M3	rs75370437	2.56E−07	0.055	intra	*ACSM1, ACSM3*	–	–	–	–	–	*ACSM1*
M4	rs6707596	5.53E−07	0.344	intra	*SP3*	YES	NO	SNP in LD (rs13010038)	–	YES	*AC106900.6*
M4	rs72873859	5.65E−07	0.296	intra	*HEATR5B*	–	–	–	–	*–*	*STRN, PRKD3, RP11–288C18.1*
M5	rs66506812	3.35E−07	0.055	intra	*CSRNP3*	YES	NA	–	–	–	–
M5	rs10830974	7.21E−07	0.360	inter	*SLC36A4, MTNR1B*	–	–	–	–	–	–
M6	rs73866245	1.35E−07	0.023	inter	*PCOLCE2, PAQR9, U2SURP*	–	–	–	–	–	–
M6	rs114943986	1.37E−07	0.037	inter	*PRR16*	–	–	–	–	–	–
M6	rs2462337	3.52E−07	0.271	inter	*CUX1*	YES	NO	YES	–	–	–
M6	rs72806699	6.39E−07	0.010	intra	*MEAK7*	–	–	–	–	–	–
M7	rs12583109	6.00E−07	0.153	intra	*NBEA, MAB21L1*	–	–	–	SNP in LD (rs9565360)	–	–

^a^TF: the nearby gene is a transcription factor.

^b^Enrichment analysis of transcription factor’s target genes in their corresponding module using F-Fisher test. NA: list of TF target genes not available.

^c^Haploreg v4.1 tool (https://pubs.broadinstitute.org/mammals/haploreg/haploreg.php), when one SNP in high LD (*r*^2^ > 0.8) is located in a regulatory region is indicated.

^d^Results from the eQTL analysis in our samples.

^e^GTEx v8 (https://www.gtexportal.org/home/).

## Discussion

In the present study, we used a network-based approach to identify novel ADHD-associated modules of co-expressed genes in PBMCs. To further investigate the biological significance of the ADHD-associated networks identified, we performed a comprehensive characterization of each module by performing enrichment analysis in biological pathways and drug or miRNA target genes. We also performed an integrative analysis by combining transcriptomic, genetic and epigenetic data on ADHD and run an eQTL analysis to identify genetic variants that could regulate the ADHD-associated modules of co-expressed genes. Our results identified seven ADHD-associated modules of co-expressed genes and support that the study of gene correlation networks may improve our understanding of the complex molecular systems underling ADHD.

Two of the ADHD-associated co-expression modules identified (M1 and M7), were enriched in genes involved in *posttranscriptional regulation of gene expression* and *epigenetic modifications*, two relevant pathways in the pathogenesis of ADHD [6]. In the same line, we found enrichment in target genes for several miRNAs in these two modules. In particular, the expression of four of them (hsa-miR-142–5p, hsa-miR-181a-5p, hsa-miR-192–5p and hsa-miR-215–5p) also correlates with the eigengene profile of module M1, pointing them as potential upstream regulatory mechanisms underlying the M1 co-expression network. Some of these miRNAs have been previously related to ADHD, like miR-192–5p upregulated in PBMCs of ADHD patients [16], and comorbid psychiatric disorders, such as miR-192–5p and miR-215–5p that were differentially expressed in the dorsolateral prefrontal cortex of major depression patients [40] or miR-181a-5p extensively related to drug addiction both in mice and human studies [41–45]. Interestingly, these miRNAs share many target genes, suggesting a complex and redundant regulatory system, particularly in the case of miR-291–5p and miR-215–5p which recognize the same seed sequence. Several of these miRNAs may regulate a number of central genes (those with high intramodular connectivity) from module M1, such as *CPSF6* encoding a subunit of a cleavage factor required for the RNA cleavage and polyadenylation processing, which was previously related to externalizing behaviors including ADHD [46], and *RICTOR*, which plays an essential role during the neurodevelopment and has been associated with hyperactivity and reduced anxiety-like behavior in conditional *knock-out* mice in the dorsal neural progenitor cells [47].

Module M1, as well as M2 and M6, were also enriched in genes that encode proteins involved in the *processing of messenger RNA* (mRNA), which includes any process related to the conversion of a primary mRNA transcript into one or more mature mRNAs. mRNA processing and alternative splicing are key processes for both the diversification of protein isoforms and the spatio-temporal control of transcripts, essential for the neuronal development, maturation, and synaptic function [48], and genetic variants in genes encoding these proteins have been related to rare neurodevelopmental disorders [49], as well as common psychiatric disorders like schizophrenia [50].

Module M5 was enriched in genes involved in *immune system* and *inflammatory response*, pathways known to play an important role in the development of neuropsychiatric disorders [38, 51, 52], particularly in ADHD [53]. Moreover, genes in module M5, and to a less extent in module M2, are targeted by a great variety of known therapeutic drugs, especially by those that target the immune system (including the Anatomical Therapeutic Chemical categories *Antiinfective for systemic use* and *Antineoplastic and immunomodulating agents*), pointing to genes in these co-expression networks as potential therapeutic targets. Importantly, a recent study that explored the druggable genome in ADHD also pointed to drugs to treat autoimmune disorders and malignancies as a potential novel path for the treatment of ADHD [54]. Besides, in module M5 we also found an enrichment in genes differentially expressed in ADHD patients compared with controls, suggesting that differentially expressed genes in ADHD cases are co-expressed and participate in the same biologic pathways. Furthermore, this enrichment was also significant when considering only highly connected genes, highlighting that the genes differentially expressed are central nodes highly connected in this network, reinforcing their relevance in the pathophysiology of ADHD.

The integrative analysis of transcriptomics, genomics, and epigenomics data on ADHD revealed that genes in module M4, also involved in the *regulation of gene expression* and *epigenetic mechanisms*, were enriched in both genetic and epigenetic signatures previously described for ADHD [3, 7]. We used two complementary approaches to assign ADHD-associated SNPs to genes, based on position or eQTL results, and found consistent results. *PNPLA2* and *IQSEC1* were the central genes in the module more significantly associated with ADHD using both methods. *PNPLA2* encodes an enzyme involved in the hydrolysis of triglycerides in adipose tissue, and has been related to obesity [55], a highly comorbid disorder in ADHD [56]. In addition, a recent study pointed *PNPLA2* as one of the most high-confidence causal genes for ADHD, after combining GWAS, eQTL and gene expression data [57]. *IQSEC1* encodes a guanine nucleotide exchange factor, essential for the maintenance of glutamatergic synapses [58], one of the key neurotransmitter systems involved in the pathophysiology of ADHD in combination with dopamine [59, 60].

The eQTL analysis did not reveal any genetic variant that overcame the genome-wide significance threshold, but we found 12 independent genomic loci that showed suggestive evidence of association (*P* < 1e−06) with the different module eigengenes. We identified a genetic variant associated with the co-expression module M1, rs62096513, which is located in a blood and brain regulatory region of a transcription factor, *ZSCAN30*, and regulates its expression in PBMCs. Interestingly, module M1 was enriched in target genes for ZSCAN30 that is also included in the same module, suggesting that this transcription factor is an upstream regulator of the co-expressed genes in the module. Besides, we identified another genetic variant associated with the M1 module eigengene, rs73170578, located in *CNTNAP2*, which encodes a neuronal transmembrane protein member of the neurexin superfamily that function as cell adhesion molecules and receptors. Both rare and common genetic variants in *CNTNAP2* have been associated with neurodevelopmental disorders [61, 62], with a special relevance in ADHD and autism [63, 64]. In addition, module M4 was associated with rs6707596, that is an eQTL of the *SP3* gene in PBMCs, a transcription factor involved in synaptic plasticity [65]. Finally, we identified four genetic variants associated with M6 module, among them, rs2462337 is located in a blood regulatory region upstream the *CUX1* gene, a transcription factor involved in the control of neuronal differentiation and the regulation of dendritic branching, spine development, and synapse formation in cortical neurons [66].

Gene networks analyses reduce the dimensionality of genome-wide gene expression data without losing important biological information and alleviate the multiple testing burden associated with the traditional gene-based methods. Similar network-based studies have been performed using gene expression data in both brain and blood in several psychiatric disorders like autism, schizophrenia and bipolar disorder [23–28]. These studies were usually performed in small sample sizes (*n* < 100 individuals), limiting their statistical power. In contrast, we improved the resolution and robustness of gene networks by considering more than 500 subjects, which allowed the identification of seven ADHD-associated modules enriched in relevant and highly significant biological pathways. However, although our transcriptomic analyses were performed mainly in medication-naive ADHD patients without comorbid disorders (93.7% of all ADHD cases), we cannot discard that these conditions may have influenced the results of the present study. So, further studies in the same cell type are required to confirm our results. Additionally, the identified modules were based on expression data from PBMCs, a non-invasive peripheral tissue whose expression profile has been proposed as a surrogate for expression profiling in the central nervous system [67], and further evidence in the brain is required to confirm their role in the pathophysiology of the disorder.

In summary, we conducted a multi-step analysis to identify and characterize modules of co-expressed genes associated with ADHD using expression data from PBMCs in ADHD cases and controls. We identified seven ADHD-associated modules of co-expressed genes, some of them being enriched in both genetic and epigenetic signatures for ADHD and on biological pathways relevant for psychiatric disorders, such as the regulation of gene expression, epigenetic mechanisms and immune signaling. We also found preliminary evidence for some potential regulatory mechanisms, including microRNAs and genetic variants, for some of the ADHD-associated modules of co-expressed genes identified. These results pinpoint promising genes and pathways for ADHD, support the use of peripheral blood to assess gene expression signatures for the disorder and highlight that the combination of multi-omics signals provides deeper and broader insights into the biological mechanisms underlying the disorder.

## Supplementary information

Supplementary information

Supplementary tables
