# Supplementary material for: Comprehensive analysis of omics data identifies relevant gene networks for Attention-Deficit/Hyperactivity Disorder (ADHD)
Source: Transl Psychiatry. 2022 Sep 24;12:409. doi: 10.1038/s41398-022-02182-8 (PMC9509350; doi:10.1038/s41398-022-02182-8)
Supplement: Supplementary file 1 — Supplementary information [file 41398_2022_2182_MOESM1_ESM.docx]

**Comprehensive analysis of omics data identifies relevant gene networks for Attention Deficit/Hyperactivity Disorder (ADHD).**

Judit Cabana-Domínguez, PhD^1,2,3,4^, María Soler Artigas, PhD^1,2,3,4^, Lorena Arribas^1,2^, Silvia Alemany, PhD^1,2^, Laura Vilar-Ribó, MSc^1,2,3^, Natalia Llonga, MSc^1,2^, Christian Fadeuilhe, PhD^1,2,3,5^, Montse Corrales, PhD^1,2,3,5^, Vanesa Richarte, PhD^1,2,3,5^, Josep Antoni Ramos-Quiroga, PhD^1,2,3,5^, Marta Ribasés, PhD^1,2,3,4^.

**SUPPLEMENTARY MATERIALS AND METHODS:**

**Clinical assessment**

#### An in-house sample of 274 adult ADHD cases (59.1% male, mean age=34.2 years, s.d=11.7) and 282 controls (57.1% male, mean age=36.5 years, s.d=9.9) was recruited. Clinical assessment was conducted by structured interviews and self-reported questionnaires as previously described ^1^, based in two different steps: (i) assessment of ADHD diagnosis based on symptomatology using the Conner's Adult ADHD Diagnostic Interview for DSM-IV (CAADID) and (ii) assessment of the severity of ADHD symptoms, the levels of impairment and the presence of comorbid disorders to increase the diagnostic accuracy with the Conners’ ADHD Rating Scale (CAARS), the ADHD Rating Scale (ADHD-RS), the Clinical Global Impression (CGI), the Wender Utah Rating Scale (WURS), the Sheehan Disability Inventory (SDS), and the Structured Clinical Interview for DSM-IV Axis I and II Disorders (SCID-I and SCID-II). Exclusion criteria were IQ < 70; a history or the current presence of a condition or illness, including neurologic, metabolic, cardiac, liver, kidney, or respiratory disease; a chronic medication of any kind; birth weight ≤ 1.5 kg; and other neurological or systemic disorders that might explain ADHD symptoms. All cases were evaluated and recruited prospectively from a restricted geographic area in a specialized out-patient program for adult ADHD at the Hospital Universitari Vall d'Hebron of Barcelona (Spain).

#### The control sample consisted of unrelated blood donors matched by sex with the clinical group. Individuals with ADHD symptomatology were excluded retrospectively from the control sample under the following criteria: (1) diagnosed with ADHD previously and (2) answering positively to the life-time presence of the following ADHD symptoms: (a) often has trouble in keeping attention on tasks, (b) usually loses things needed for tasks, (c) often fidgets with hands or feet or squirms in seat, and (d) often gets up from seat when remaining in seat is expected.

**Transcriptome profiling**

#### *RNA isolation and microarray assays*

PBMCs were separated by Ficoll density gradient method immediately after blood extraction, and total mRNA was isolated using Qiazol Lysis reagent and the RNAeasy Midi Kit (QIAgen, Hilden, Germany). RNA integrity and concentration of samples were assayed by 2100 Bioanalyzer (Agilent Technologies Inc., Santa Clara, CA, USA). RNA was retrotranscribed using the Ambion WT Expression Kit (Life Technologies, Carlsbad, CA, USA). The cDNA was subsequently fragmented, labeled, and hybridized with the GeneChip WT Terminal Labeling and Hybridization Kit (Affymetrix, Santa Clara, CA, USA). Samples were hybridized to the GeneChip Human Gene 1.1 ST 96-Array plate (Affymetrix), covering a total of 36,079 transcripts that correspond to 21,014 genes. The array processing and data generation were assessed using the Gene Titan Affymetrix microarray platform.

#### *Bioinformatics and statistical analyses*

Raw data was pre-processed as previously described ^1^. In brief, data was processed with the Robust Multichip Analysis (RMA) algorithm (background correction and normalization) implemented in the *OligoR* R-package ^2^. Sample outliers were removed using the *arrayQualityMetrics* R-package ^3^. Transcript probes corresponding to unknown genes, genes in non-autosomal chromosomes or matching to multiple genes in the GRCh37/hg19 human genome build were discarded, ending up with 19,004 probes corresponding to 18,055 unique genes. Microarray batch effects and non-biological experimental variation (RNA integrity number (RIN), age and gender) were adjusted for using the *empiricalBayesLM* algorithm included in WGCNA R package ^4,5^.

**Weighted Gene Correlation Network Analysis (WGCNA)**

Modules of co-expressed genes were identified from processed transcriptomic data by the *WGCNA* R-package ^5^. First, sample hierarchical clustering was performed to detect outliers (4 cases and 3 controls; Supplementary Figure S1a), ending up with 270 ADHD cases and 279 controls. A soft-thresholding power of 4, meeting a degree of independence of 0.85 with the minimum power value, was selected (Supplementary Figure S1b). One-step network construction and module detection was performed considering an unsigned network type with default values, including a minimal module size of 30 and a cut height for module merging of 0.25. This method identifies correlated networks assigning each gene to a module independently of any phenotype and represents the gene expression for each module by a module eigengene.

**Enrichment analyses in the ADHD-associated co-expression modules: Expression in brain areas**

Expression in brain regions was analyzed using the *ABAEnrichment* R-package ^6^ and data from the Developing Human Brain Atlas, which include information of 27 brain regions of five developmental stages: prenatal, infant (0-2 yrs), child (3-11 yrs), adolescent (12-19 yrs) and adult (> 19 yrs). Genes were annotated to brain regions using default expression cut-offs, i.e. 10%-steps of expression quantiles across all brain regions were used. Enrichment analysis was then performed using a hypergeometric test. The threshold for significant enrichment was set at meanFWER < 0.05.

**Integrative analysis of ADHD-associated co-expression modules and ADHD omics data**

*ADHD genetic signatures*

Because of sample overlap with our in-house sample and to avoid biases, we removed the Spanish study (572 cases and 425 controls) and considered GWAS summary statistics from Demontis et al. ^7^ on a restricted sample of 18,527 cases and 33,769 controls ^7^. Gene-based analyses were run in MAGMA v1.08 ^8^ using the SNP-wise mean model, which computes the gene-based statistic based on the sum of the assigned SNP -log(10) P values. SNPs were assigned to genes using two different approaches: (i) a positional-approach that assigns SNPs to their nearest gene considering only SNPs located within the transcribed region (based on NCBI 37.3 gene definitions) +/- 1Kb and (ii) eMAGMA ^9,10^, a modified version of the positional-approach on MAGMA that integrated cis-eQTL information from the GTEx project v8 ^11^, based on significant SNP-gene associations in whole blood. In both cases, MAGMA accounts for gene length, number of SNPs in a gene and linkage disequilibrium between markers, using as a reference panel the European ancestry samples from the 1000 Genomes Project, phase 3 ^12^. Then, a competitive gene-set analysis was performed using P-values obtained from each gene-based analysis to test whether genes in a specific co-expression module were more strongly associated with ADHD than other genes, while correcting for confounding effects such as gene length and size of the gene set. The Bonferroni correction was applied to correct for multiple testing (P < 0.05/7 modules < 7.1E-03).

*ADHD methylation signatures*

Three different approaches to adjust for the number of CpGs per gene were considered: (i) *methylglm*, which incorporates the number of CpGs as a covariate in logistic regression, (ii) *methylRRA*, which adjusts for multiple P-values of each gene by Robust Rank Aggregation, and then applies a Preranked version of Gene Set Enrichment Analysis (GSEA Preranked) in gene-set testing (both methods from the *methylgsa* R package ^13^), and (iii) *gsameth*, which adjusts the number of CpGs for each gene by weighted resampling and Wallenius non-central hypergeometric approximation (from *missMethyl* R package ^14^).

**Gene module eQTL analysis**

Genetic information was available from a subset of 495 individuals included in the WGCNA (91.3 %): 231 ADHD subjects (average age at assessment of 33.8 years (s.d. = 11.6)) and 264 controls (average age at assessment of 36.7 years (s.d. = 10.1)). Samples were genotyped using the HumanOmni 2.5 or the Infinium Global Screening Array MD v2.0 Illumina arrays. Genotyping data was preprocessed according the RICOPILI pipeline (<https://sites.google.com/a/broadinstitute.org/ricopili/overview>) ^15^ and imputed with the Michigan Imputation Server ([https://imputationserver.sph.umich.edu/index.html#](https://imputationserver.sph.umich.edu/index.html)!) ^16^ using the European ancestry subgroup of the 1000 Genomes phase 3 as reference panel. SNPs with an imputation score (INFO score) < 0.8 or minor allele frequency (MAF) < 0.01 were discarded, ending up with 5,257,362 SNPs. Normality for all module eigengene was asserted by Kolmogorov-Smirnov test (P > 0.05), Kurtosis |≤ 4| and Skewness |≤ 1| (Supplementary Table S1). For those module eigengenes not following a normal distribution, data was transformed using the rank-based inverse normal transformation. Then, we run all seven gene-module eQTL analyses under an additive linear regression model using PLINK 1.09, adding as covariates the first ten principal components, sex, age and the genotyping wave.

*Functional annotation*

All SNPs with a P-value < 0.05 and having an LD r2 ≥ 0.6 with one of the lead SNPs were functionally annotated using the FUMA protocol (<https://fuma.ctglab.nl/>) ^17^ by combining data from Combined Annotation Dependent Depletion (CADD) scores, RegulomeDB (RDB) scores and chromatin states. CADD score predicts deleteriousness of SNPs based on 63 functional annotations (CADD threshold ≥ 12.37) ^18^. RDB is a categorical score to estimate the regulatory functionality of SNPs based on cis-eQTLs and evidence for transcription factor binding^19^. Chromatin states shows the accessibility of genomic regions with 15 categorical states predicted by ChromHMM based on 5 chromatin marks for 127 epigenomes ^20,21^. Lead SNPs in each eQTL study were further analyzed by: (i) exploring whether they lie in histone modification marks including enhancer (H3K4me1 and H3K27ac) or promoter (H3K4me3 and H3K9ac) regions in 10 different brain areas or primary mononuclear cells from peripheral blood using the Haploreg v4.1 tool (<https://pubs.broadinstitute.org/mammals/haploreg/haploreg.php>) ^22^; (ii) Evaluating their effect on gene expression through a cis-eQTL analysis using data from all brain tissues and whole blood from GTEx data v8 (<https://www.gtexportal.org/home/>) ^11^ and in our in-house data from PBMCs using linear regression analysis, after ascertain normality and homoscedasticity in the expression of genes. (iii) When a lead SNP was located nearby a transcription factor coding gene, a list of target genes for this specific transcription factor was obtained from the Gene Set Enrichment Analysis databases (GSEA; <https://www.gsea-msigdb.org/gsea/index.jsp>) ^23,24^ and an enrichment analysis in the corresponding module was performed with a F-Fisher exact test.

**Bibliography:**

1 Mortimer N, Sánchez-Mora C, Rovira P, Vilar-Ribó L, Richarte V, Corrales M *et al.* Transcriptome profiling in adult attention-deficit hyperactivity disorder. *Eur Neuropsychopharmacol* 2020; **41**: 160–166.

2 Carvalho BS, Irizarry RA. A framework for oligonucleotide microarray preprocessing. *Bioinformatics* 2010; **26**: 2363–2367.

3 Kauffmann A, Gentleman R, Huber W. arrayQualityMetrics - A bioconductor package for quality assessment of microarray data. *Bioinformatics* 2009; **25**: 415–416.

4 Schäfer J, Strimmer K. An empirical Bayes approach to inferring large-scale gene association networks. *Bioinformatics* 2005; **21**: 754–764.

5 Langfelder P, Horvath S. WGCNA: An R package for weighted correlation network analysis. *BMC Bioinformatics* 2008; **9**: 1–13.

6 Grote S, Prüfer K, Kelso J, Dannemann M. ABAEnrichment: An R package to test for gene set expression enrichment in the adult and developing human brain. *Bioinformatics* 2016; **32**: 3201–3203.

7 Demontis D, Walters RK, Martin J, Mattheisen M, Als TD, Agerbo E *et al.* Discovery of the first genome-wide significant risk loci for attention deficit/hyperactivity disorder. *Nat Genet* 2019; **51**: 63–75.

8 de Leeuw CA, Mooij JM, Heskes T, Posthuma D. MAGMA: Generalized Gene-Set Analysis of GWAS Data. *PLoS Comput Biol* 2015; **11**: e1004219.

9 Gerring ZF, Mina-Vargas A, Gamazon ER, Derks EM. E-MAGMA: An eQTL-informed method to identify risk genes using genome-wide association study summary statistics. *Bioinformatics* 2021; **37**: 2245–2249.

10 Gerring ZF, Gamazon ER, Derks EM. A gene co-expression network-based analysis of multiple brain tissues reveals novel genes and molecular pathways underlying major depression. *PLoS Genet* 2019; **15**: e1008245.

11 The GTEx Consortium. The GTEx Consortium atlas of genetic regulatory effects across human tissues. *Science* 2020; **369**: 1318–1330.

12 Delaneau O, Marchini J, McVeanh GA, Donnelly P, Lunter G, Marchini JL *et al.* Integrating sequence and array data to create an improved 1000 Genomes Project haplotype reference panel. *Nat Commun* 2014; **5**: 1–9.

13 Ren X, Kuan PF. methylGSA: a Bioconductor package and Shiny app for DNA methylation data length bias adjustment in gene set testing. *Bioinformatics* 2019; **35**: 1958–1959.

14 Maksimovic J, Oshlack A, Phipson B. Gene set enrichment analysis for genome-wide DNA methylation data. *Genome Biol* 2021; **22**: 1–26.

15 Lam M, Awasthi S, Watson HJ, Goldstein J, Panagiotaropoulou G, Trubetskoy V *et al.* RICOPILI: Rapid Imputation for COnsortias PIpeLIne. *Bioinformatics* 2020; **36**: 930–933.

16 Das S, Forer L, Schönherr S, Sidore C, Locke AE, Kwong A *et al.* Next-generation genotype imputation service and methods. *Nat Genet* 2016; **48**: 1284–1287.

17 Watanabe K, Taskesen E, Van Bochoven A, Posthuma D. Functional mapping and annotation of genetic associations with FUMA. *Nat Commun* 2017; **8**: 1826.

18 Kircher M, Witten DM, Jain P, O’roak BJ, Cooper GM, Shendure J. A general framework for estimating the relative pathogenicity of human genetic variants. *Nat Genet* 2014; **46**: 310–315.

19 Boyle AP, Hong EL, Hariharan M, Cheng Y, Schaub MA, Kasowski M *et al.* Annotation of functional variation in personal genomes using RegulomeDB. *Genome Res* 2012; **22**: 1790–1797.

20 Kundaje A, Meuleman W, Ernst J, Bilenky M, Yen A, Heravi-Moussavi A *et al.* Integrative analysis of 111 reference human epigenomes. *Nature* 2015; **518**: 317–330.

21 Ernst J, Kellis M. Large-scale imputation of epigenomic datasets for systematic annotation of diverse human tissues. *Nat Biotechnol* 2015; **33**: 364–376.

22 Ward LD, Kellis M. HaploReg v4: systematic mining of putative causal variants, cell types, regulators and target genes for human complex traits and disease. *Nucleic Acids Res* 2016; **44**: D877–D881.

23 Subramanian A, Tamayo P, Mootha VK, Mukherjee S, Ebert BL, Gillette MA *et al.* Gene set enrichment analysis: A knowledge-based approach for interpreting genome-wide expression profiles. *Proc Natl Acad Sci* 2005; **102**: 15545–15550.

24 Mootha VK, Lindgren CM, Eriksson KF, Subramanian A, Sihag S, Lehar J *et al.* PGC-1α-responsive genes involved in oxidative phosphorylation are coordinately downregulated in human diabetes. *Nat Genet 2003 343* 2003; **34**: 267–273.

**SUPPLEMENTARY FIGURES:**

a)

b)

c)

**Supplementary Figure S1. Plots from the WGCNA.** A) Clustering dendrogram of samples. The red line indicates the threshold used to identify outliers. B) Soft threshold selection process. C) Cluster dendrogram of modules. Each color represents one specific co‑expression module, and branches above represent genes.

1. Module M1


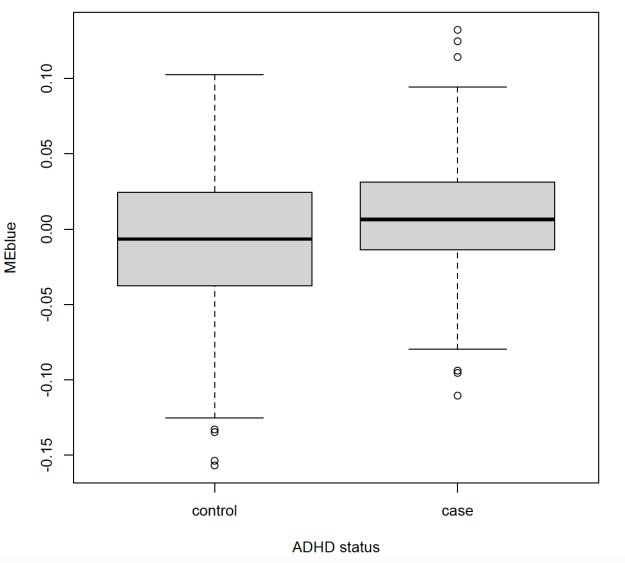

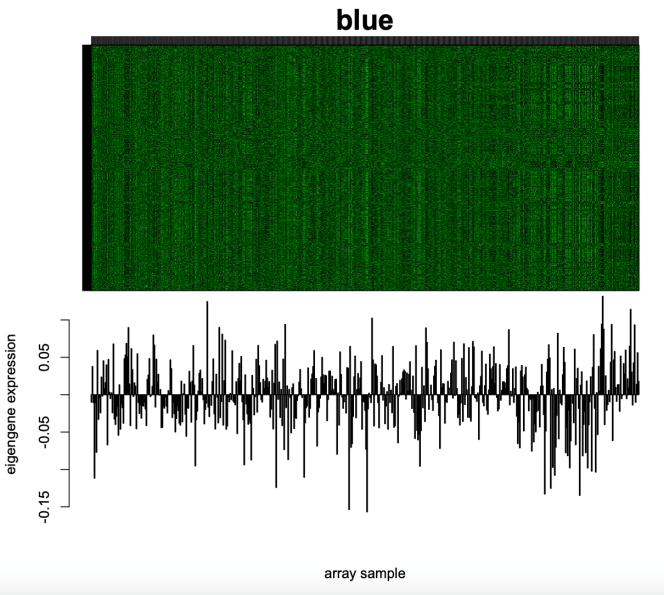


1. Module M2


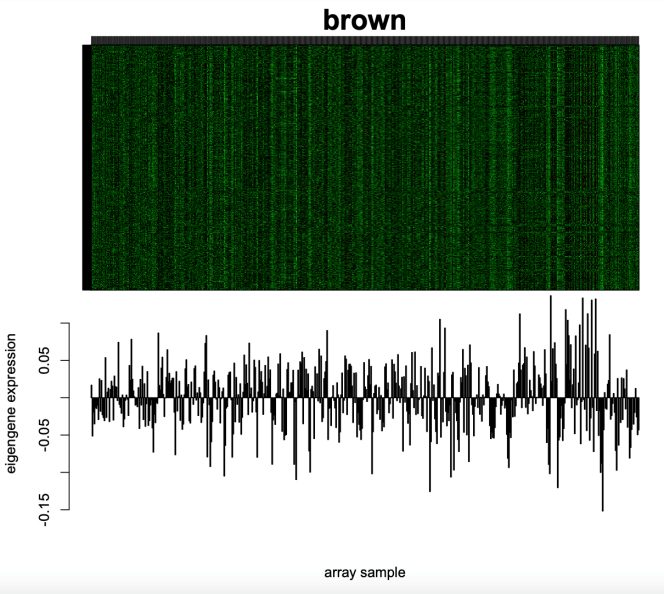

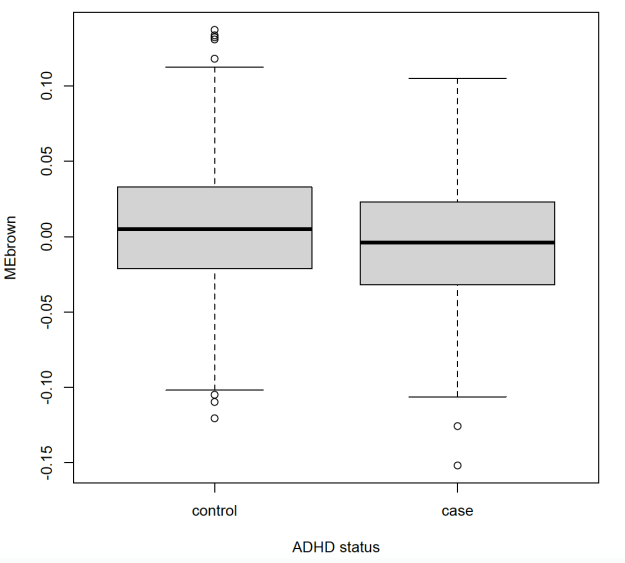


1. Module M3


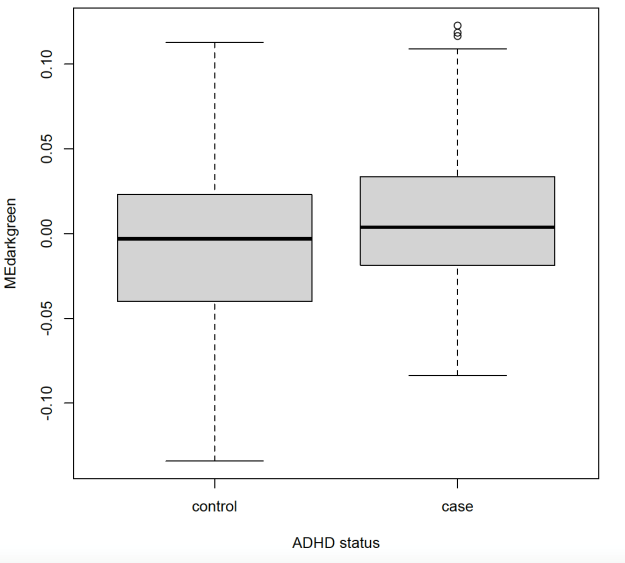

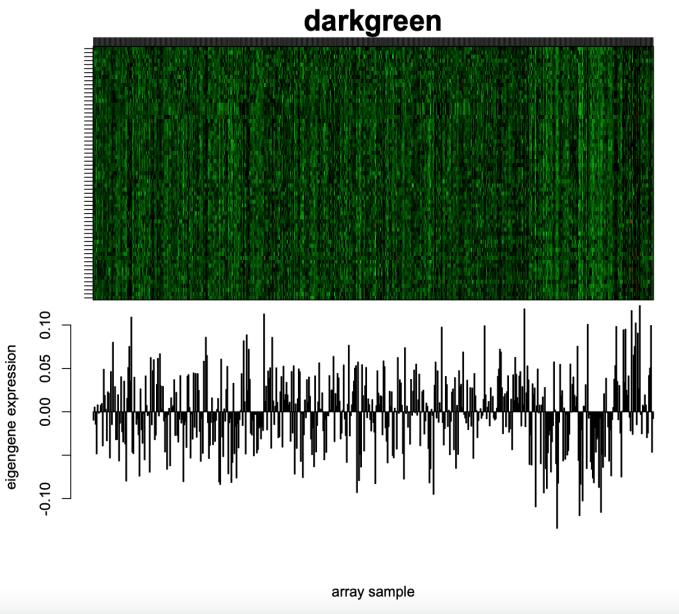


1. Module M4


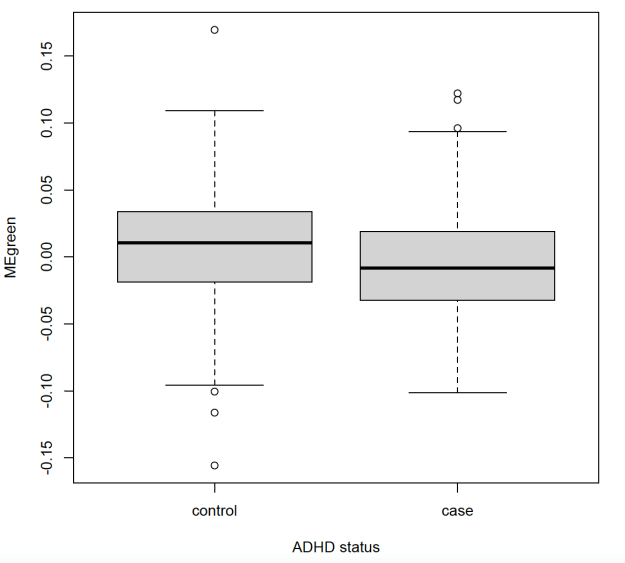

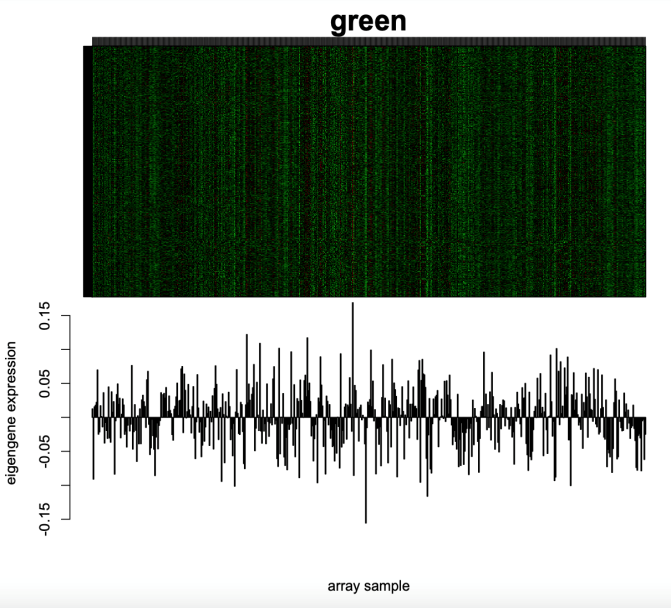


1. Module M5


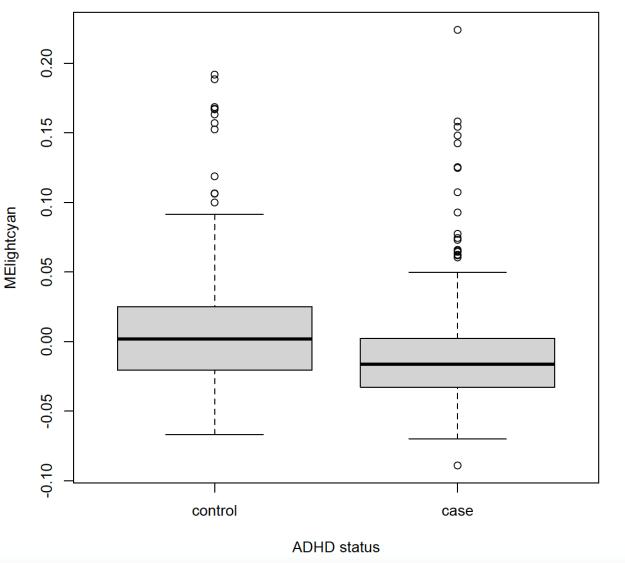

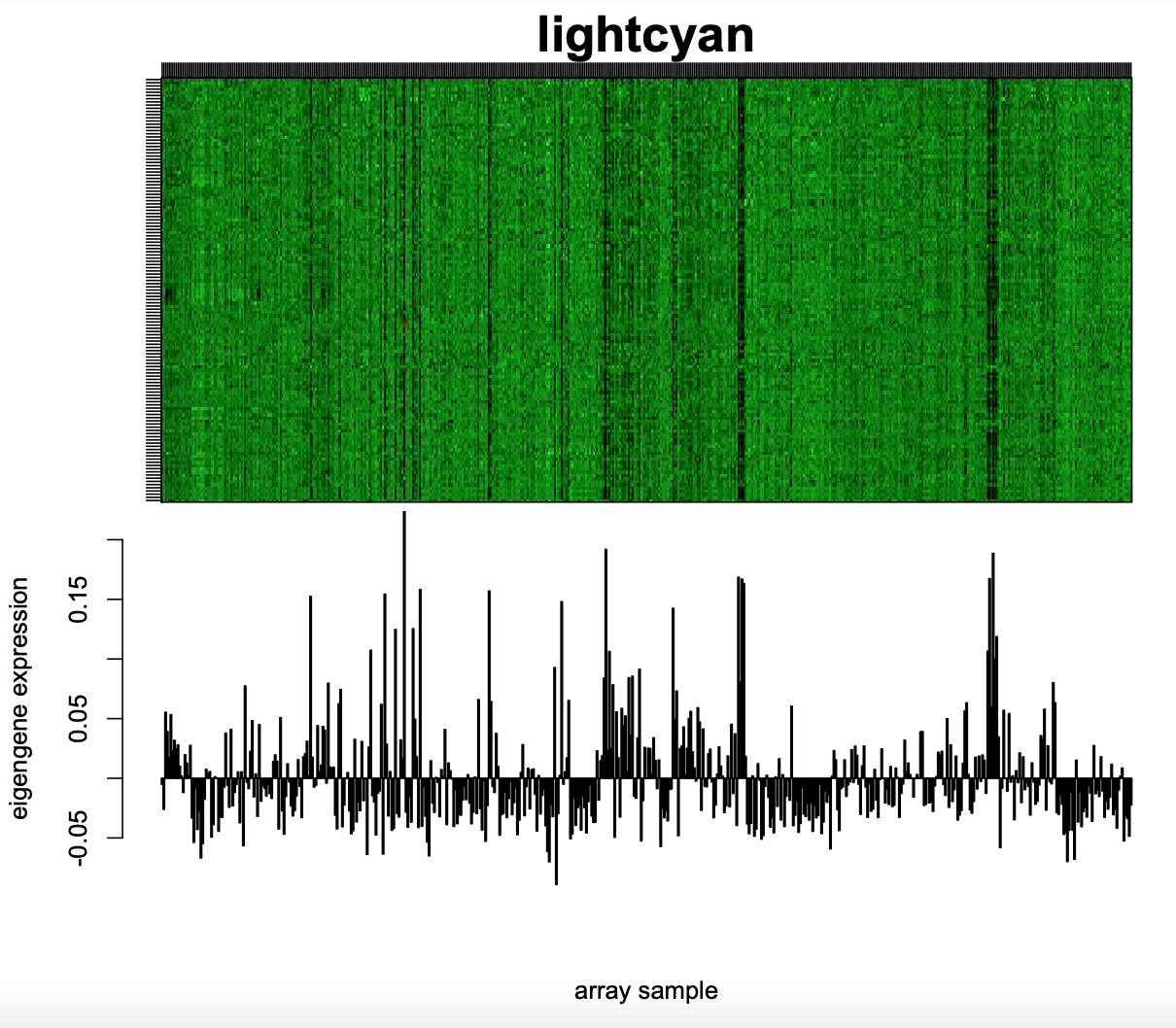


1. Module M6


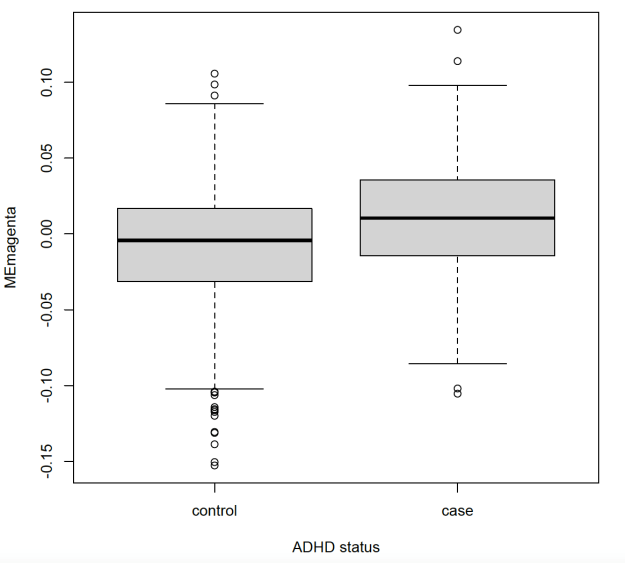

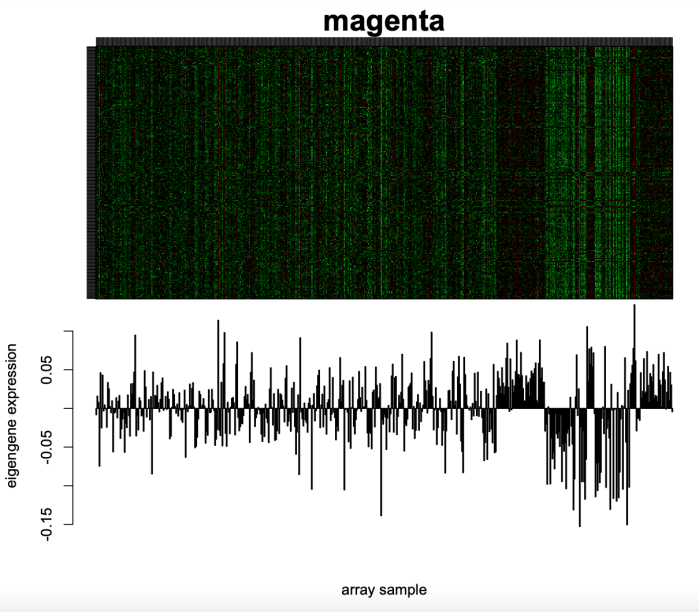


1. Module M7


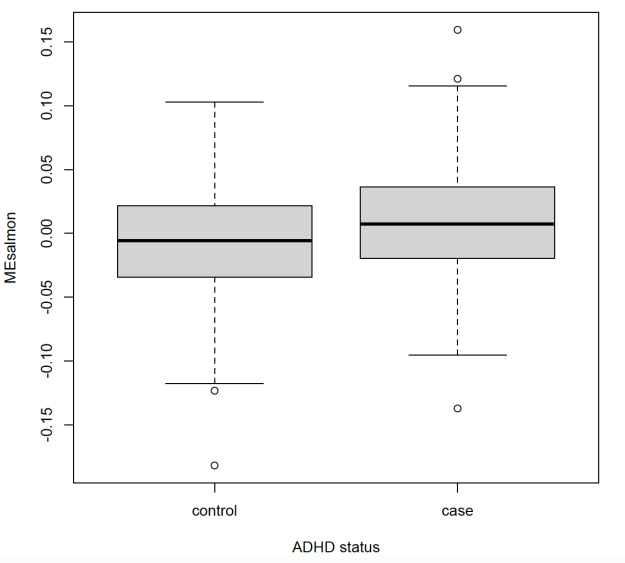

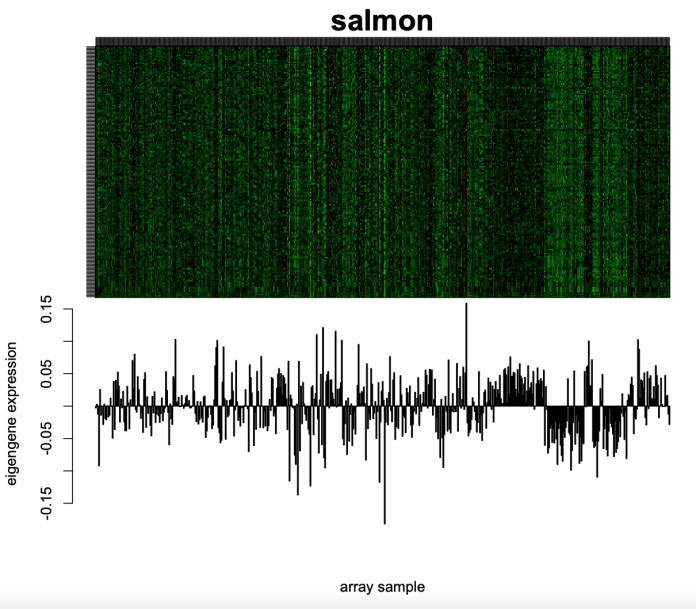


**Supplementary Figure S2. Module diagnostics for the co-expression networks.** At the left, relationship between module heatmap and module eigengene. All modules show the characteristic band structures suggestive of well-defined modules, consistent across samples. For each module. At the right, boxplot of the module eigengene grouped by ADHD status.

a) Module M1 b) Module M2


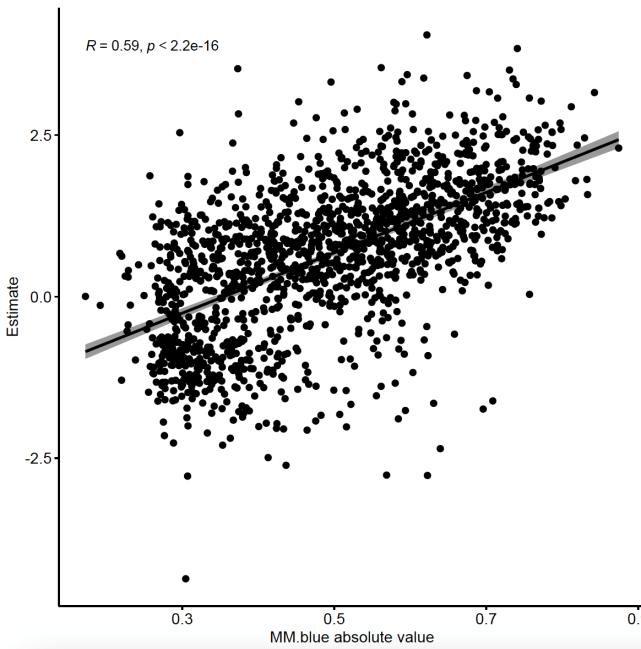

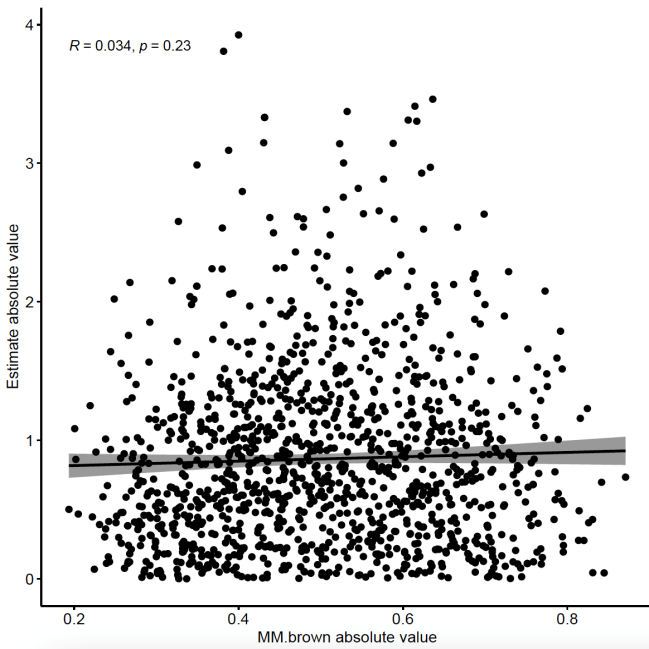


c) Module M3 d) Module M4


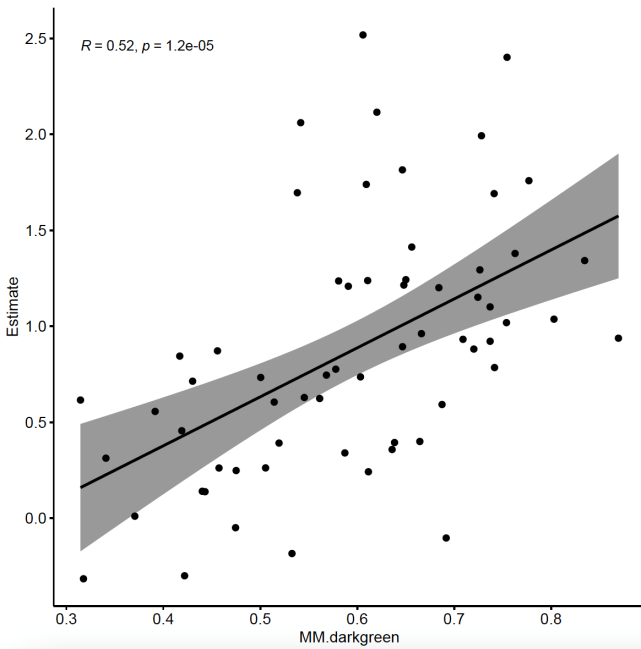

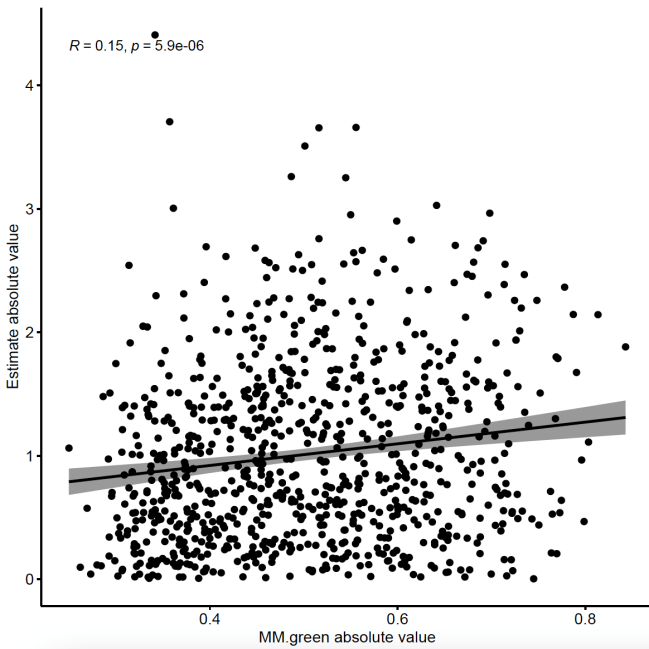


e) Module M5 f) Module M6


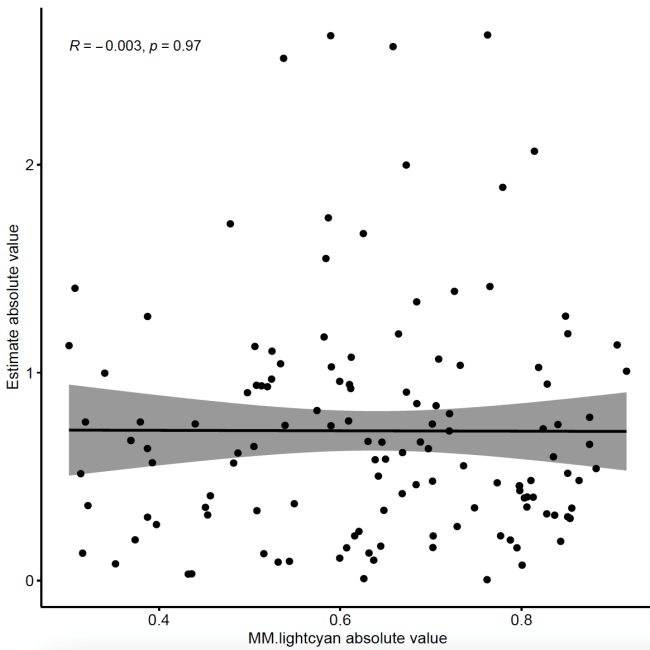

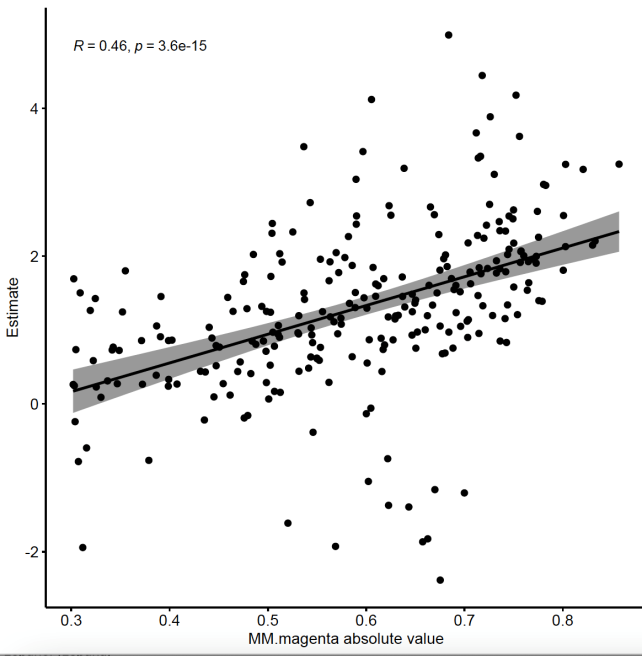


g) Module M7


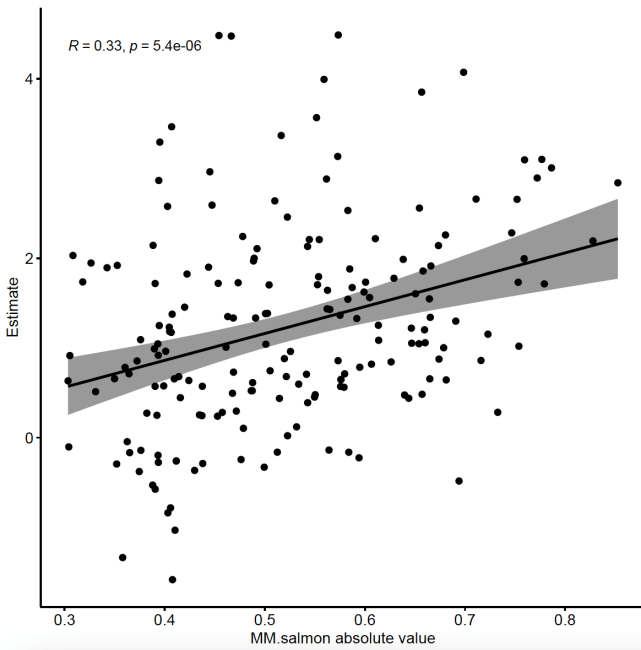


**Supplementary Figure S3. Correlation between module membership (MM) and gene significance (GS) for genes in each module.** Using the MM (an indicator of the intramodular connectivity of a gene) and GS (effect size of the association between each gene in the module and ADHD), central genes in the network that have a high significance for ADHD are identified. In all cases the absolute value for MM and the GS are plotted.

a) b)

c) d)

**Supplementary Figure S4. Correlation between M1 module eigengene (ME) and miRNA expression profiles:** a) hsa-miR-142-5p, b) hsa-miR-181a-5p, c) miR-192-5p and d) miR-215-5p.

a) Module M1 b) Module M2


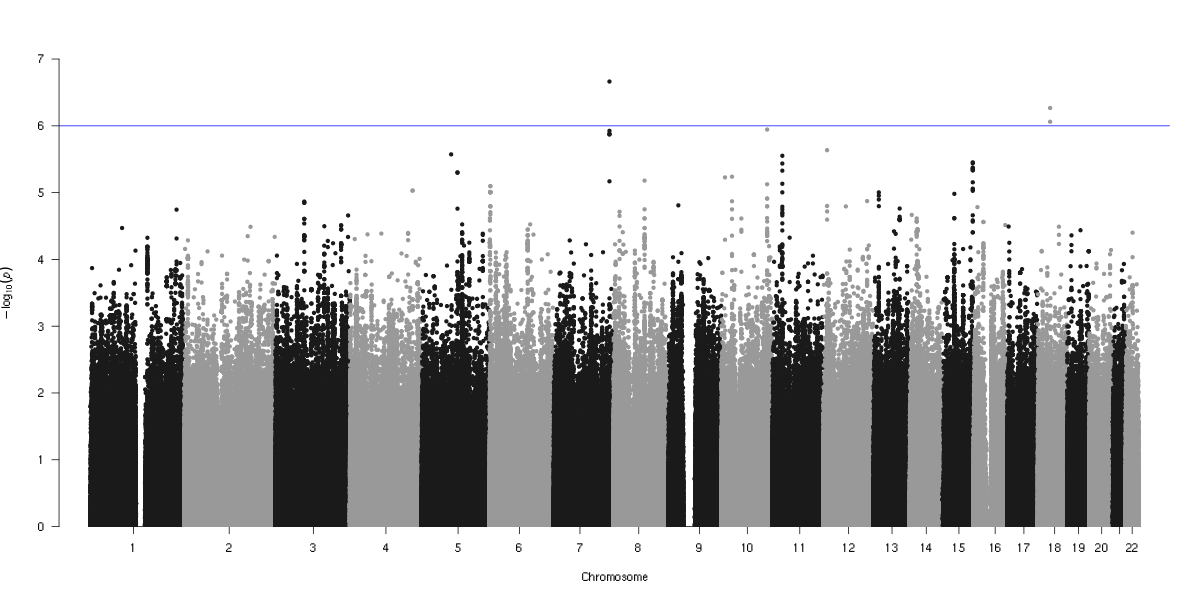

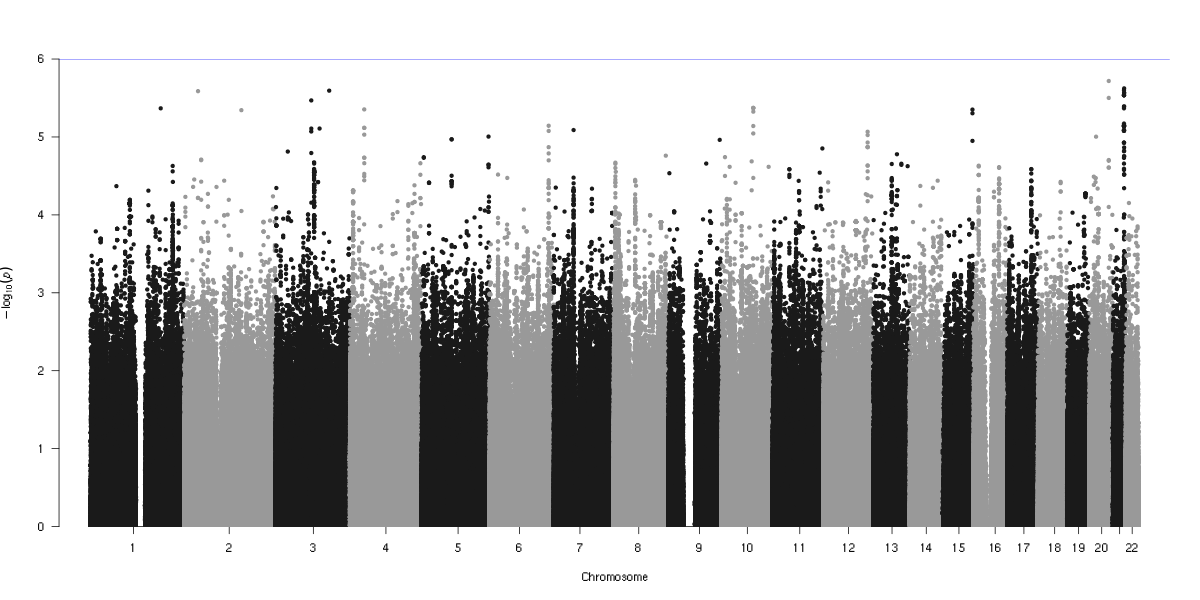


c) Module M3 d) Module M4


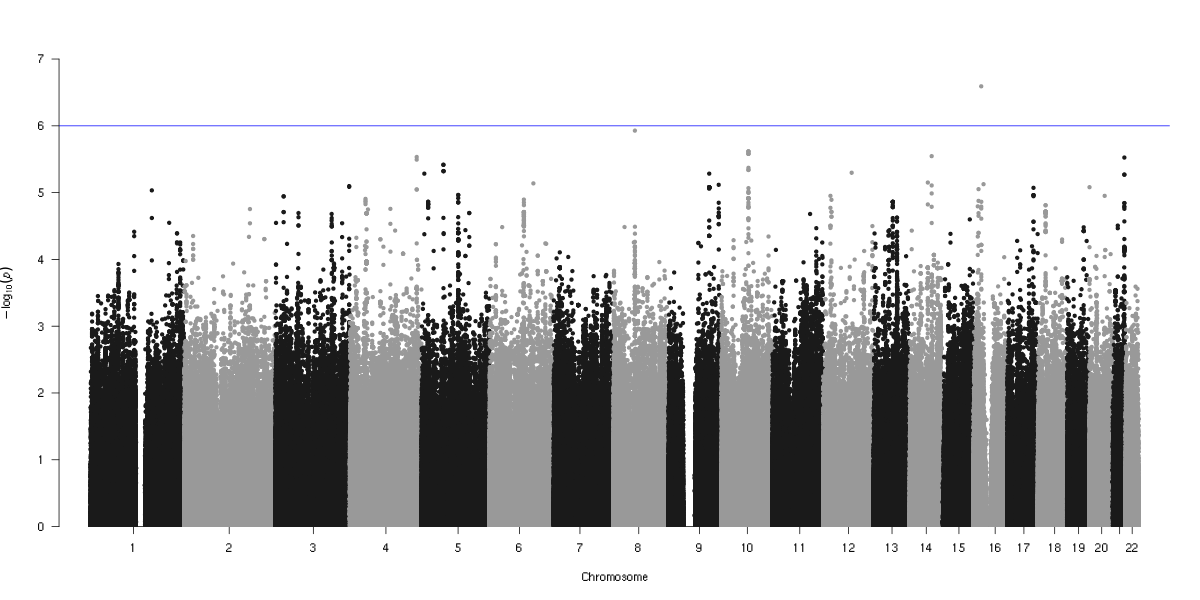

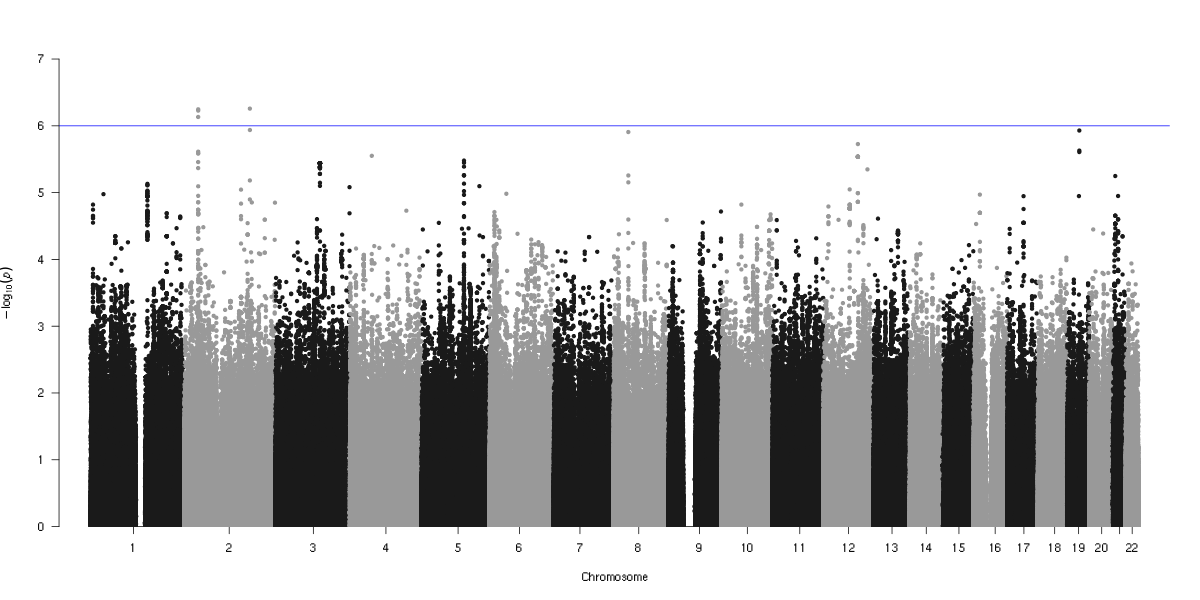


e) Module M5 f) Module M6


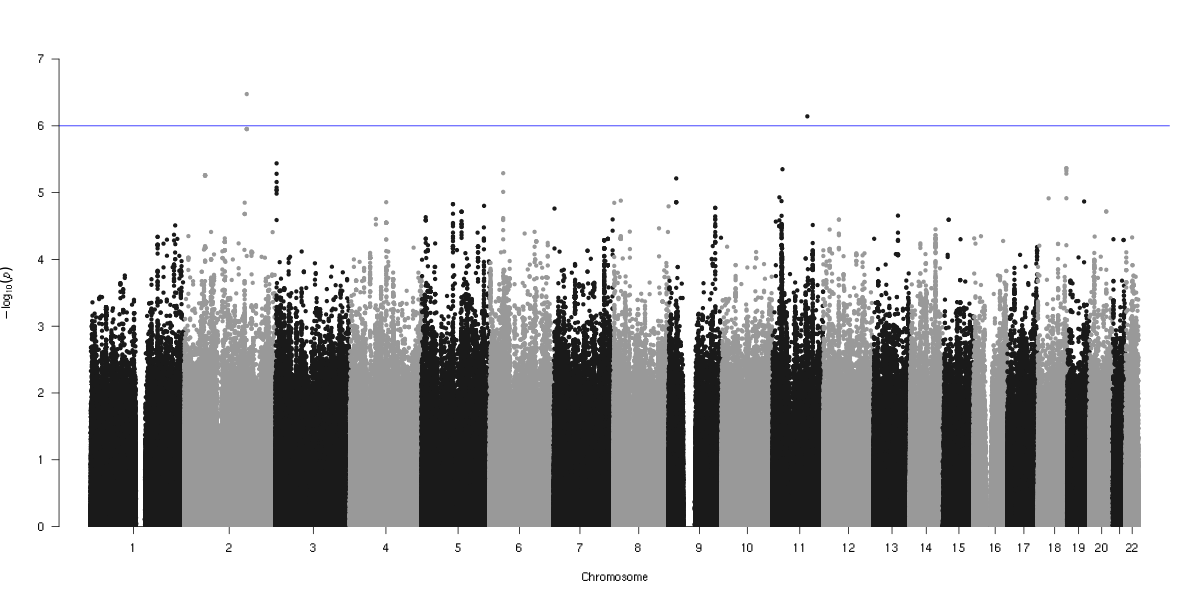

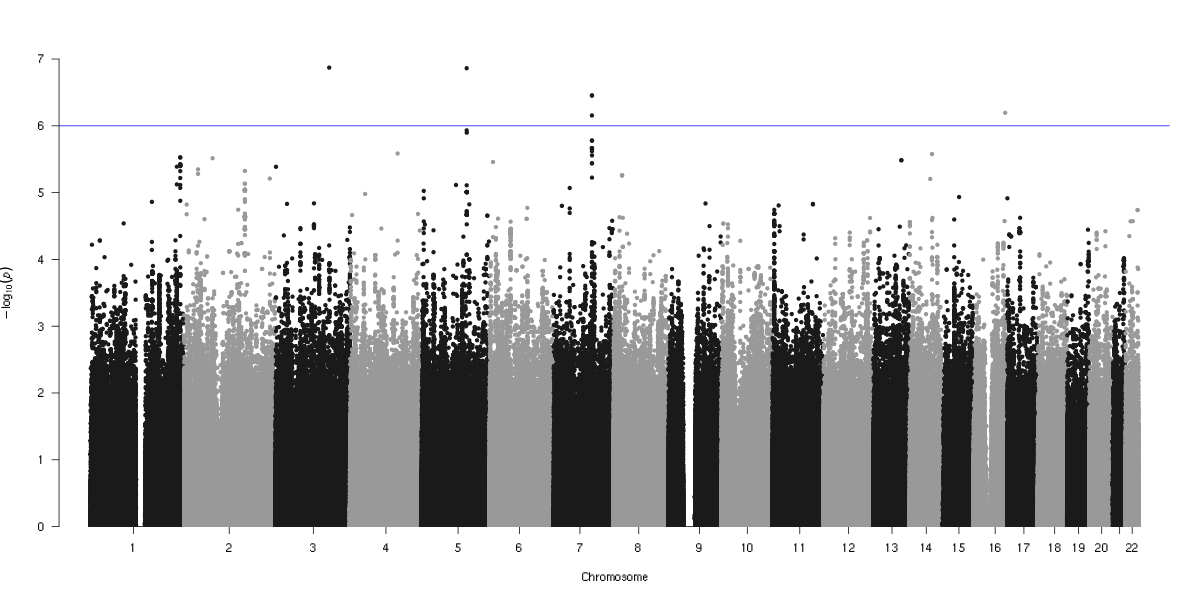


g) Module M7


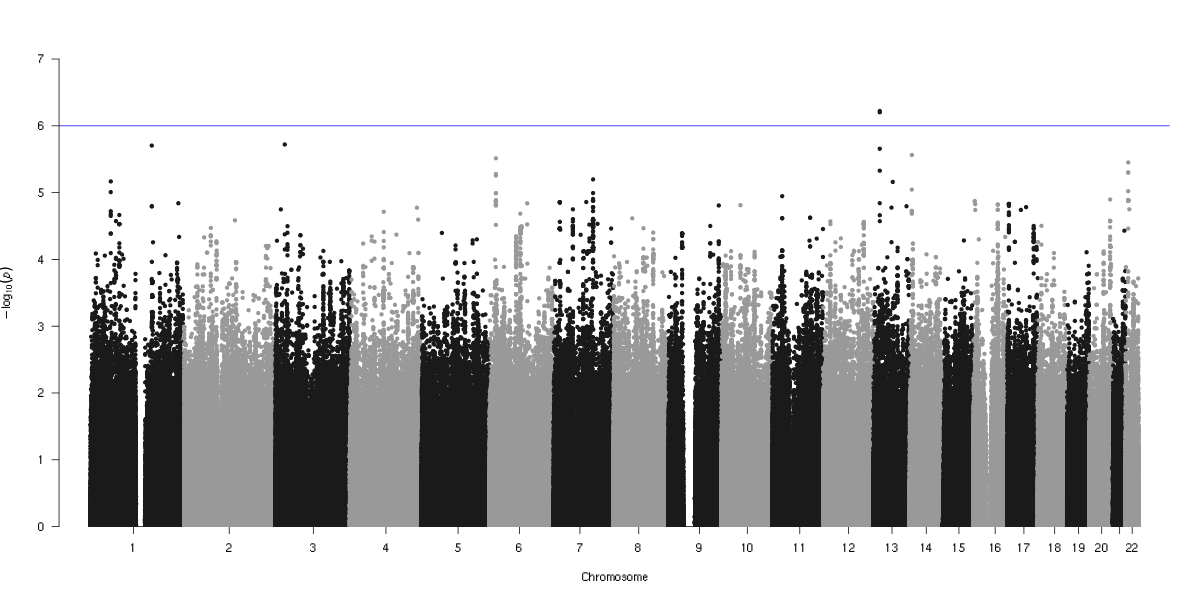


**Supplementary Figure S5. Manhattan plots from GWAS on module eigengenes.** In blue genome-wide suggestive association threshold (P < 1e-06).

a) Module M1 b) Module M2 c) Module M3


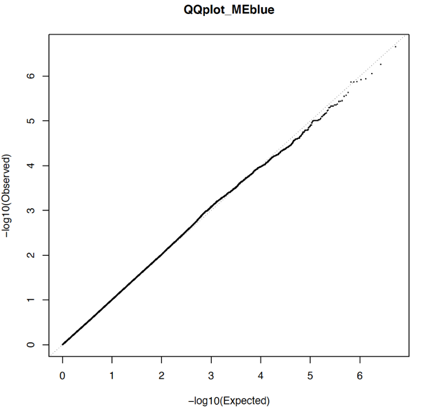

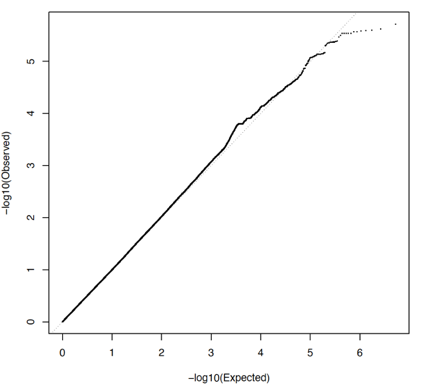

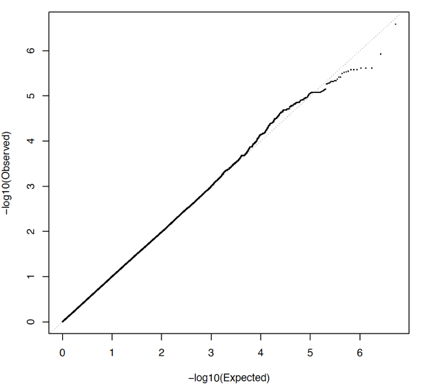


e) Module M4 f) Module M5 g) Module M6


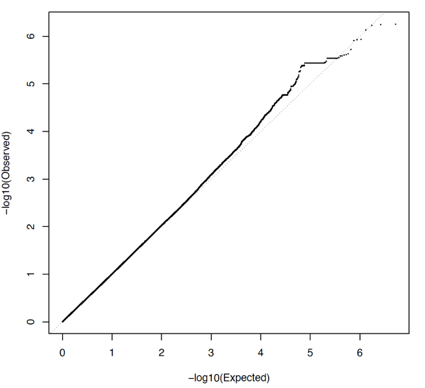

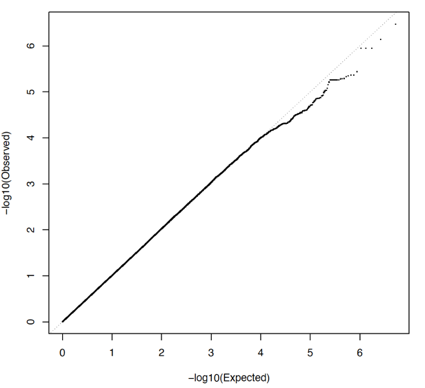

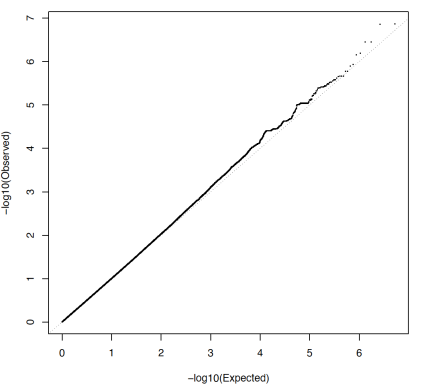


h) Module M7


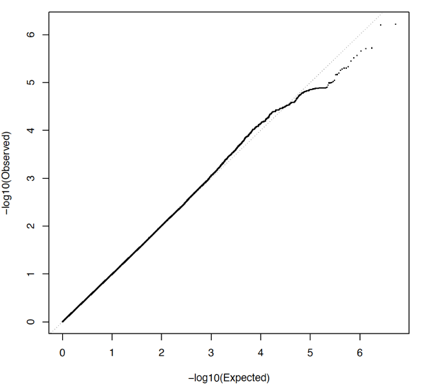


**Supplementary Figure S6. QQ plots from GWAS on module eigengenes.**

a)

**
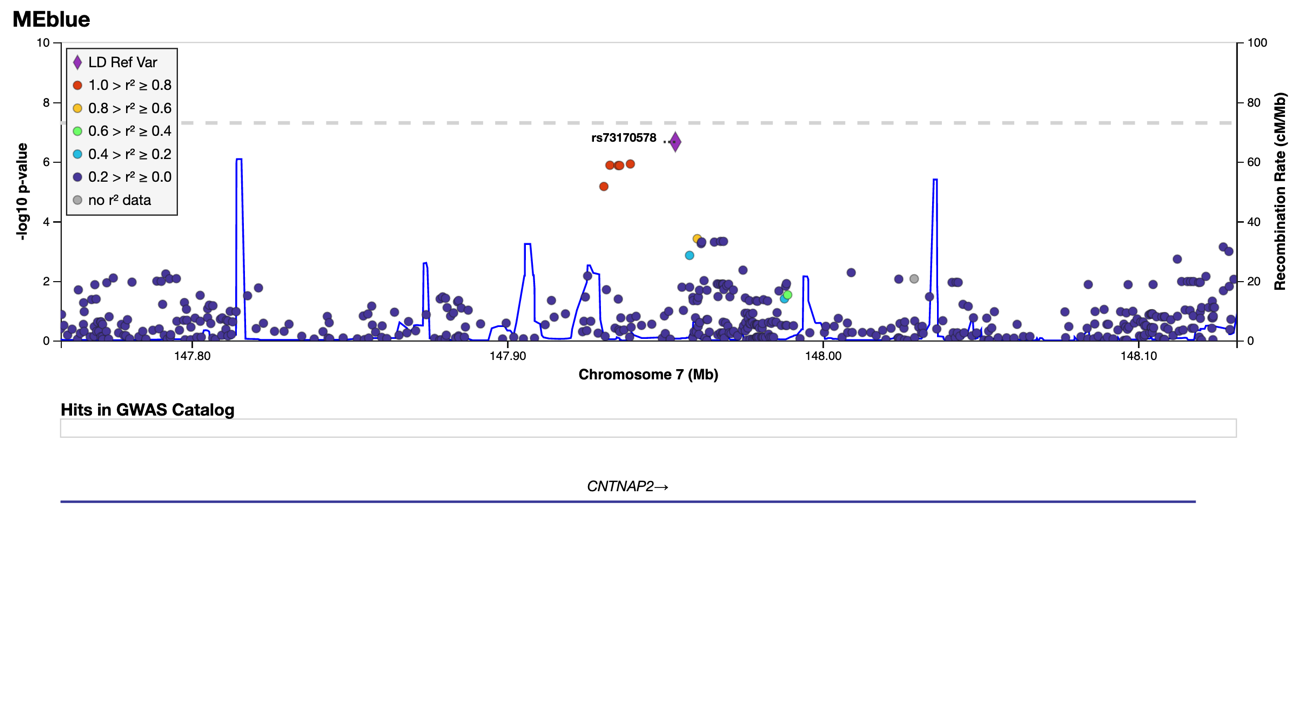
**

b)

**
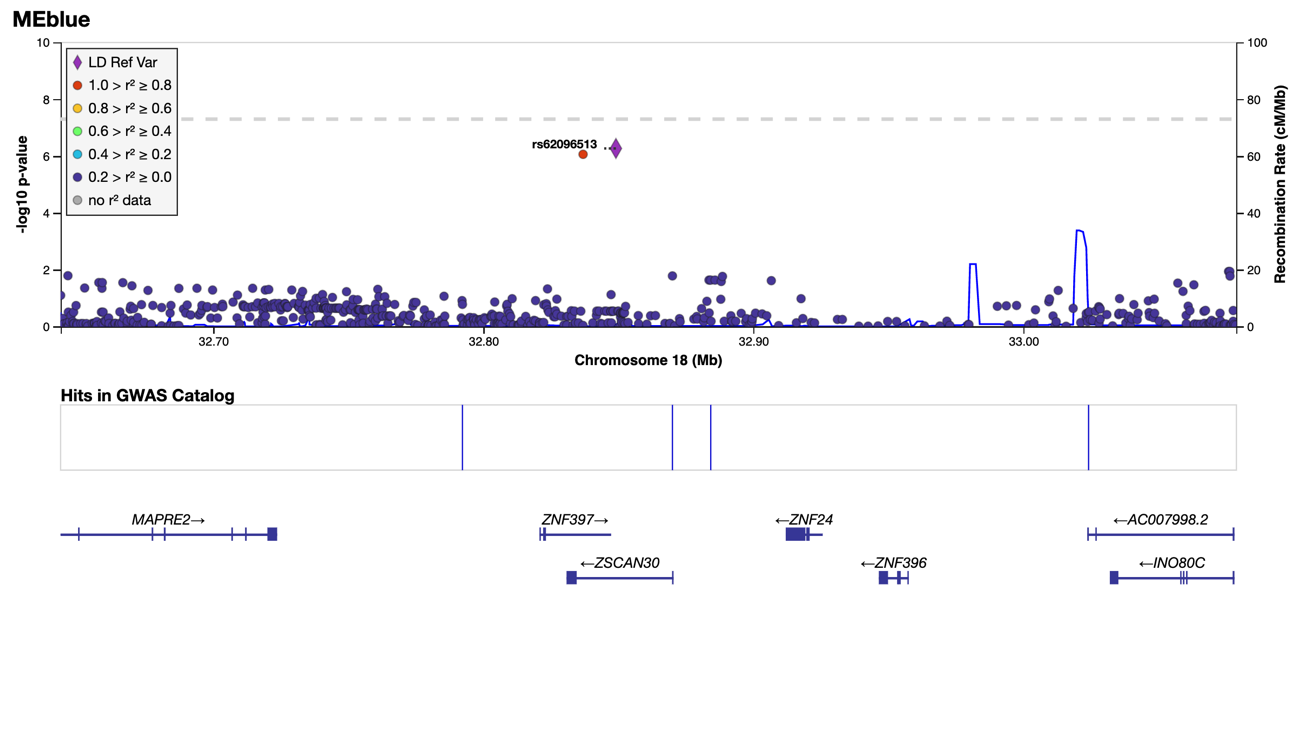
**

c)


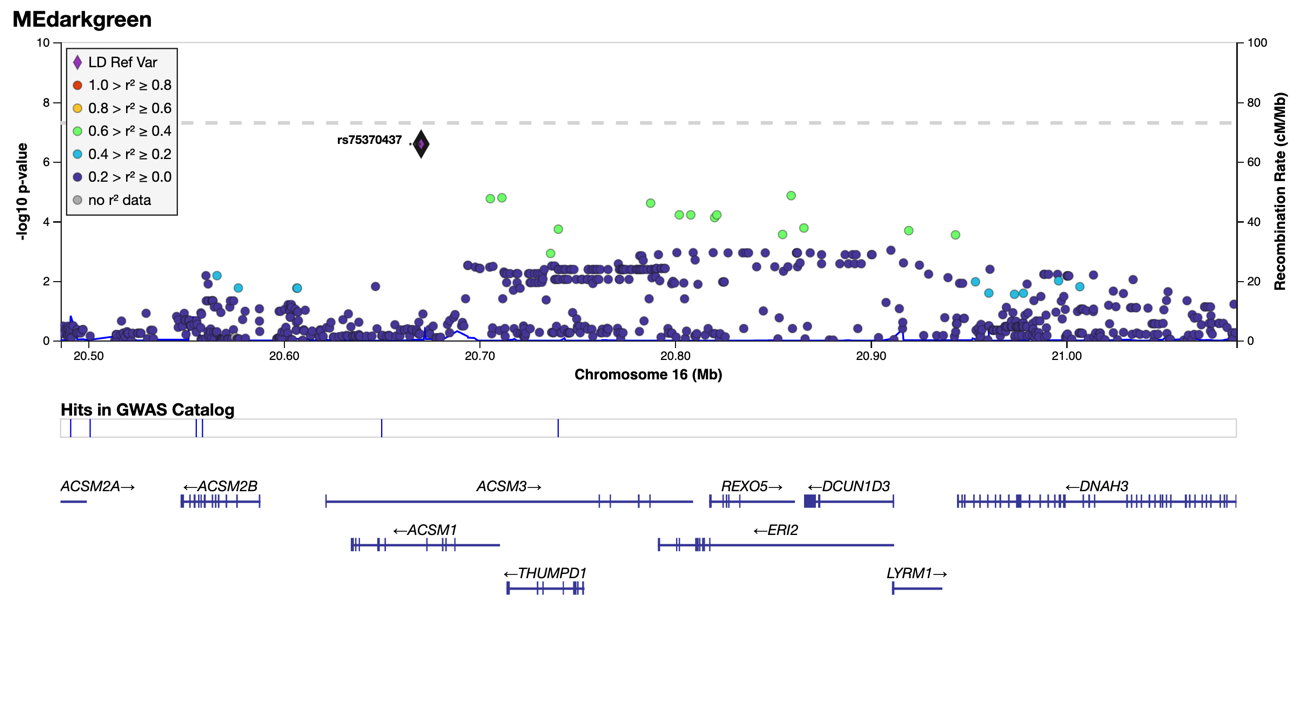


d)


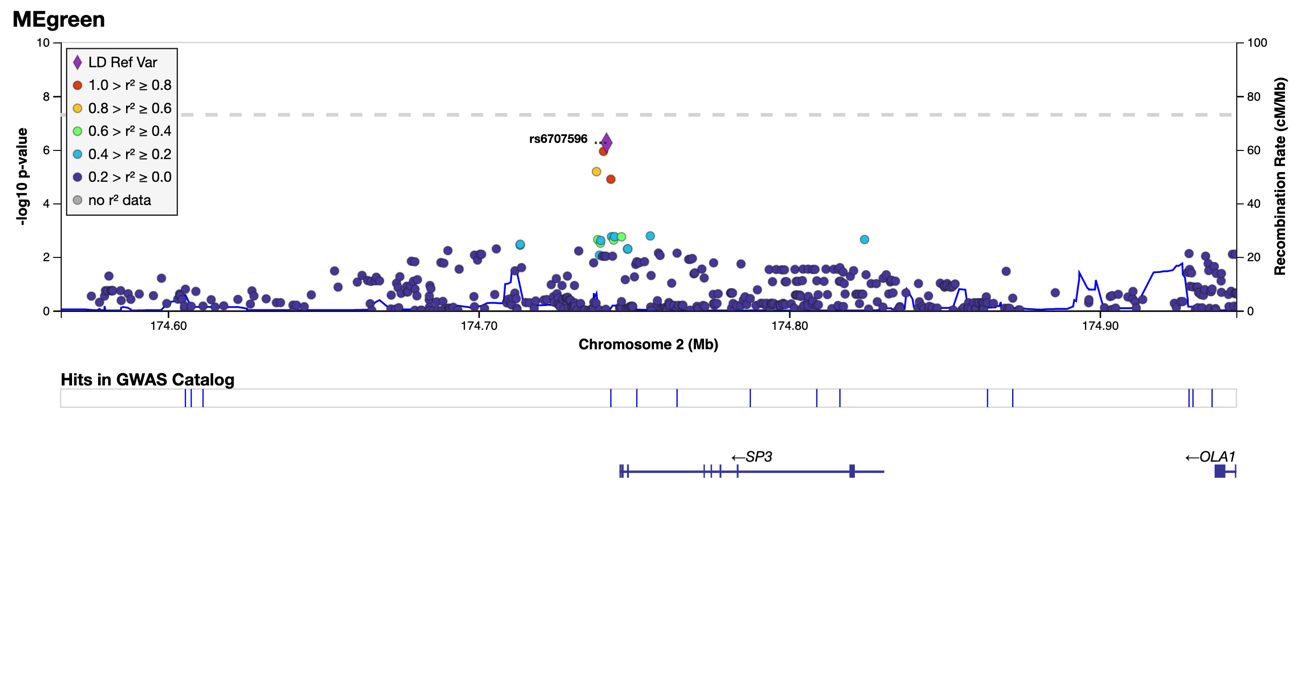


e)


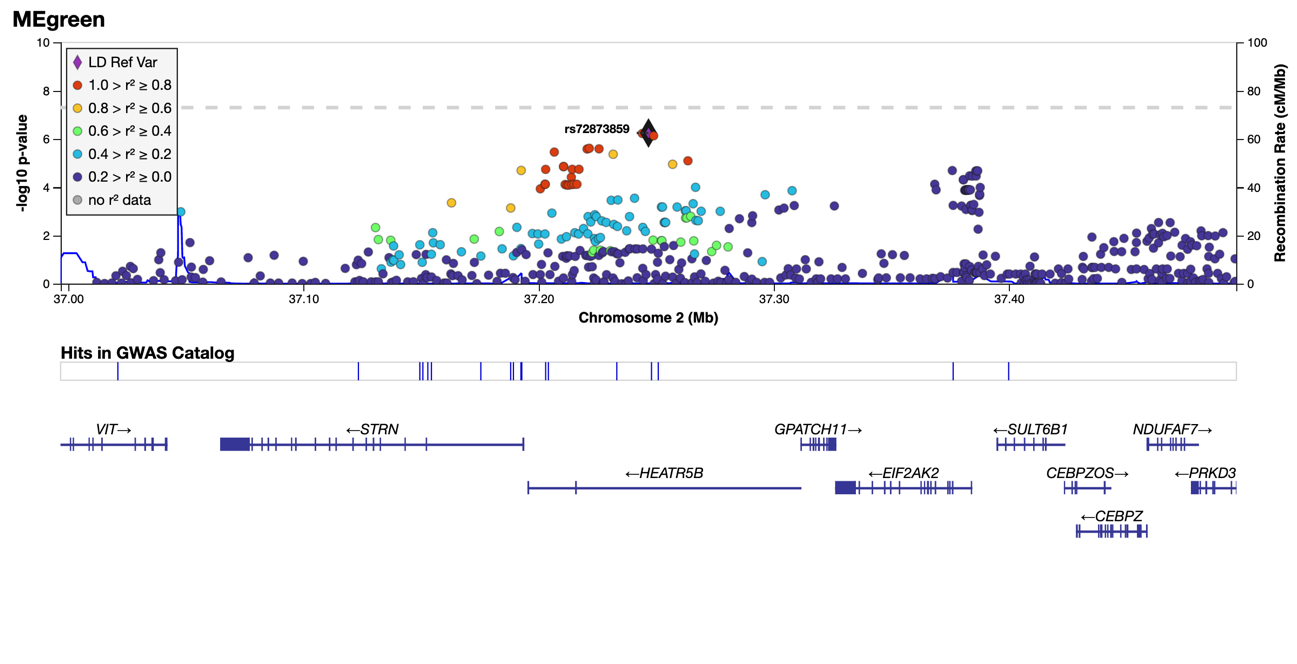


f)


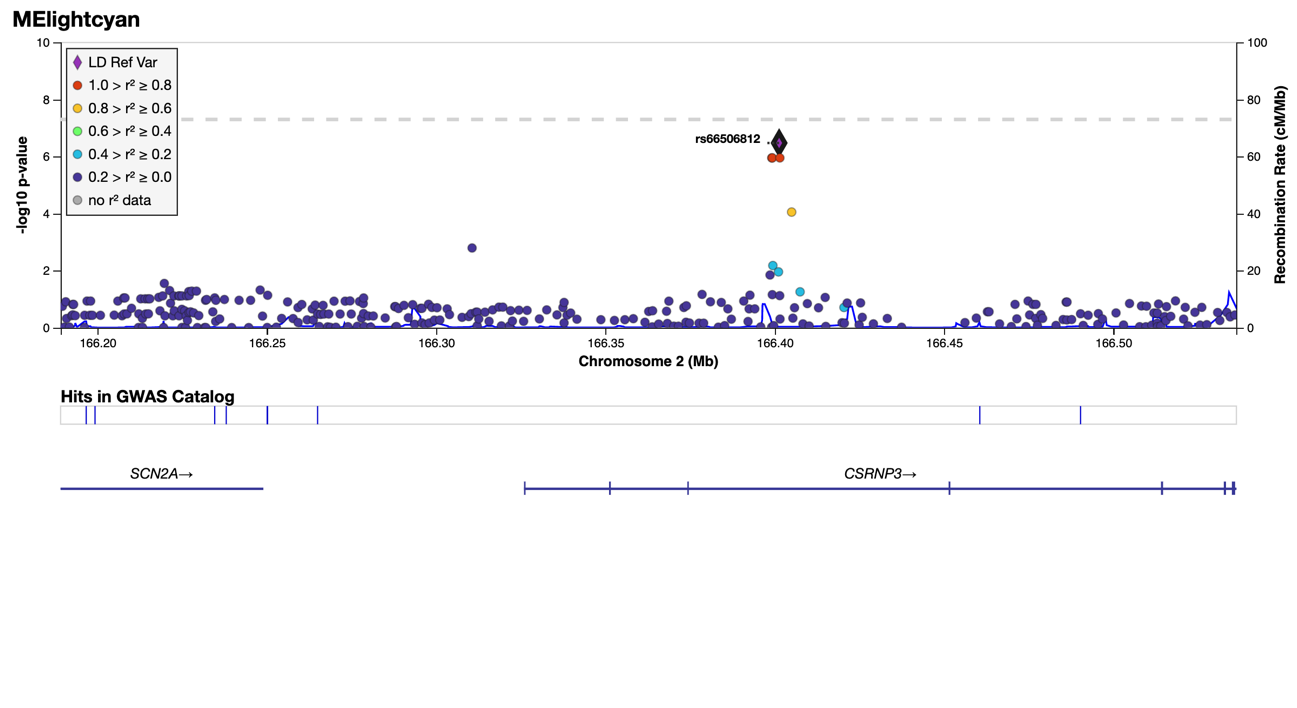


g)


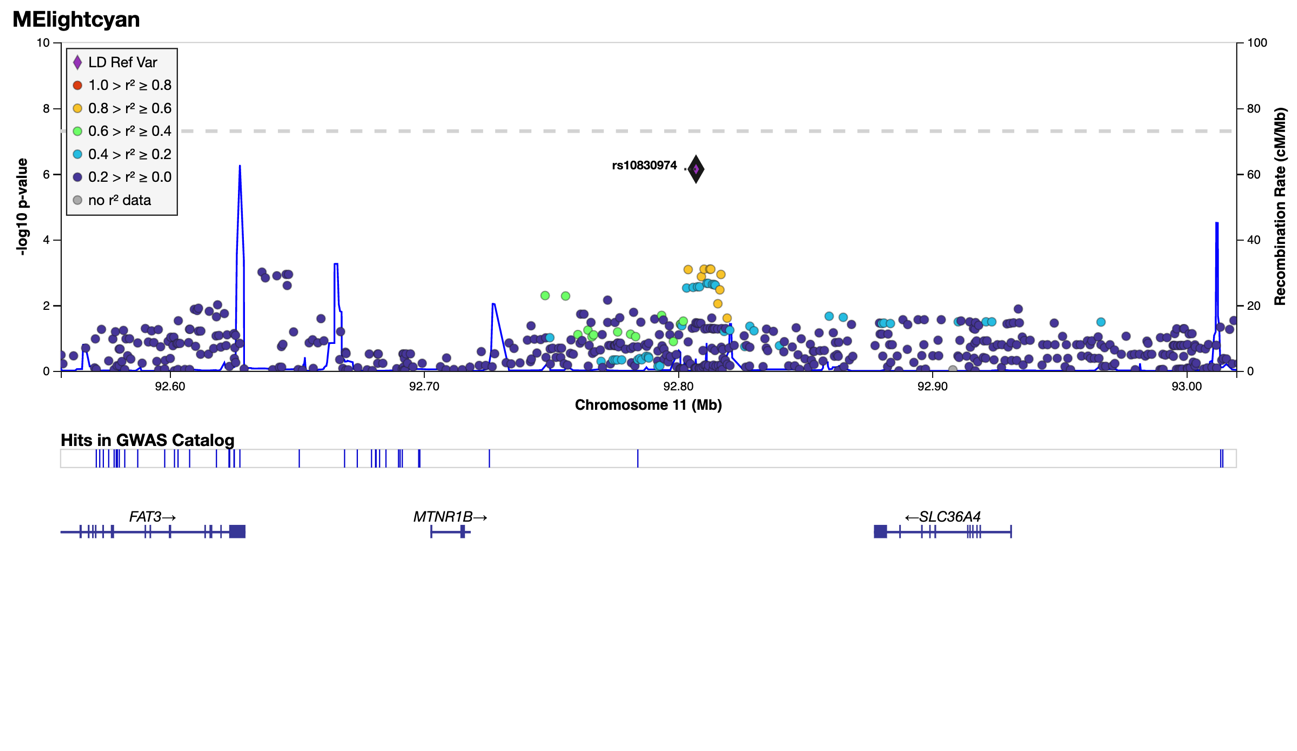


h)


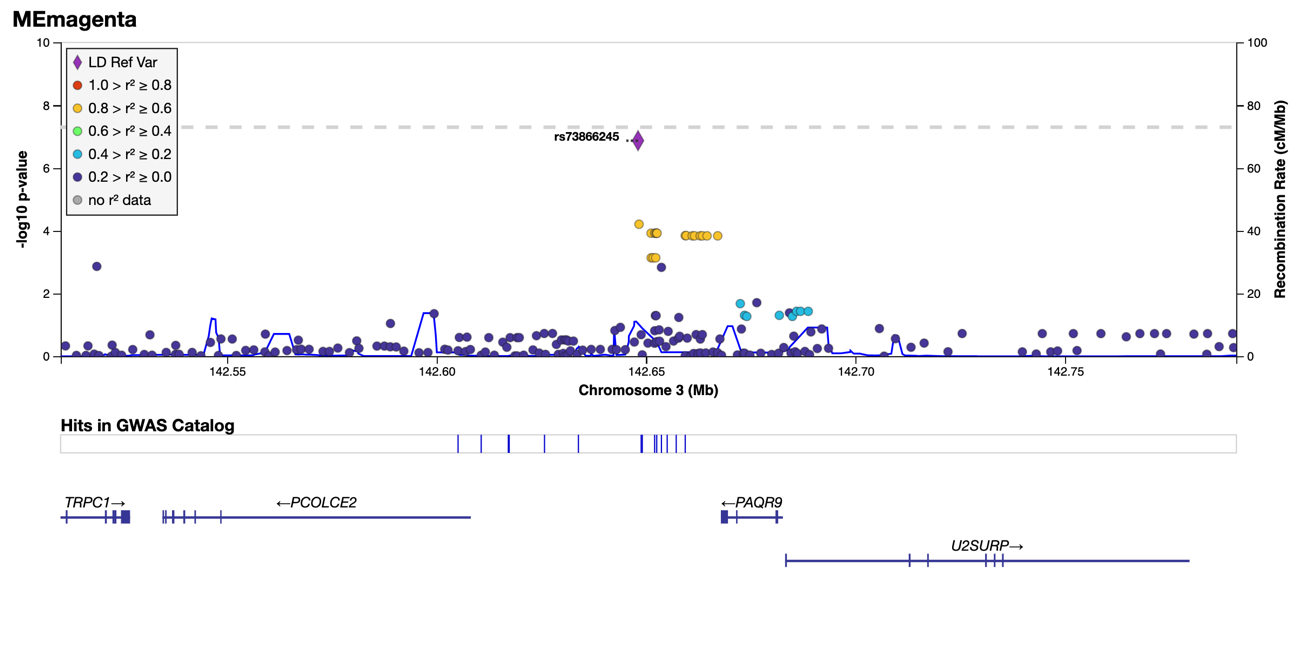


i)


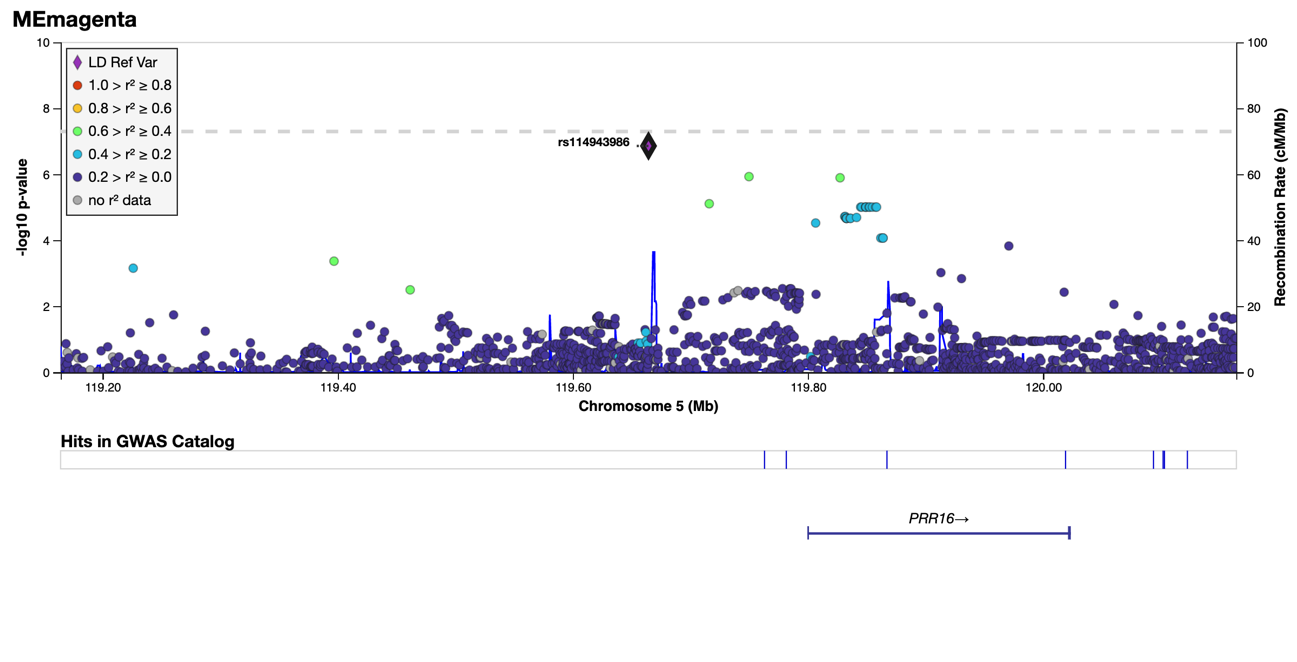


j)


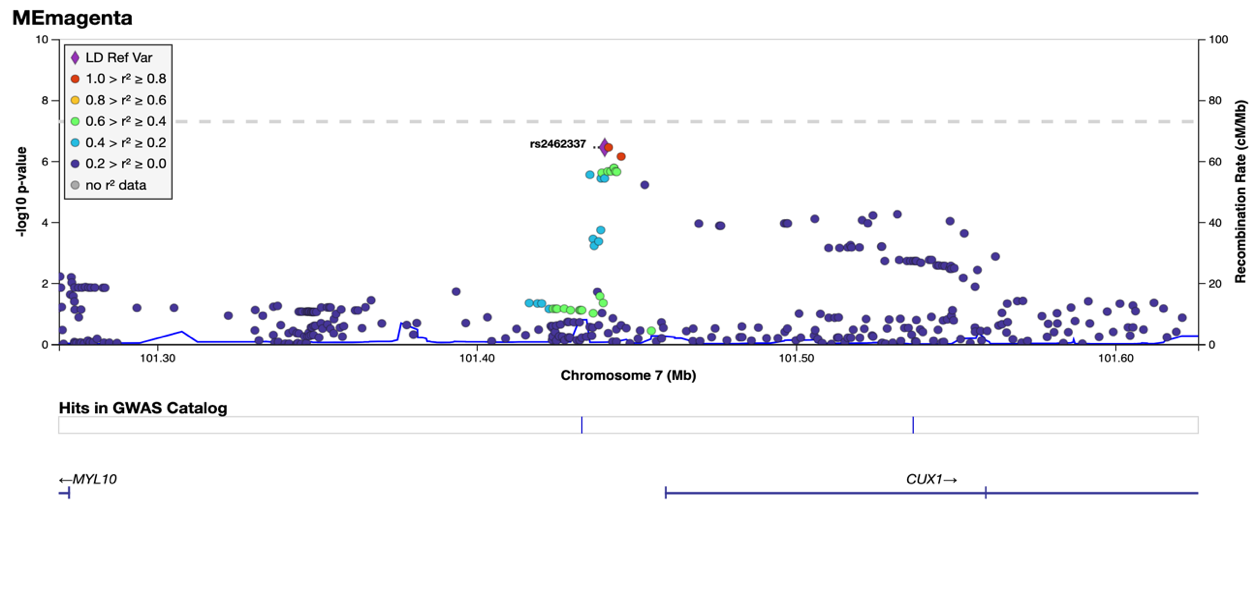


k)


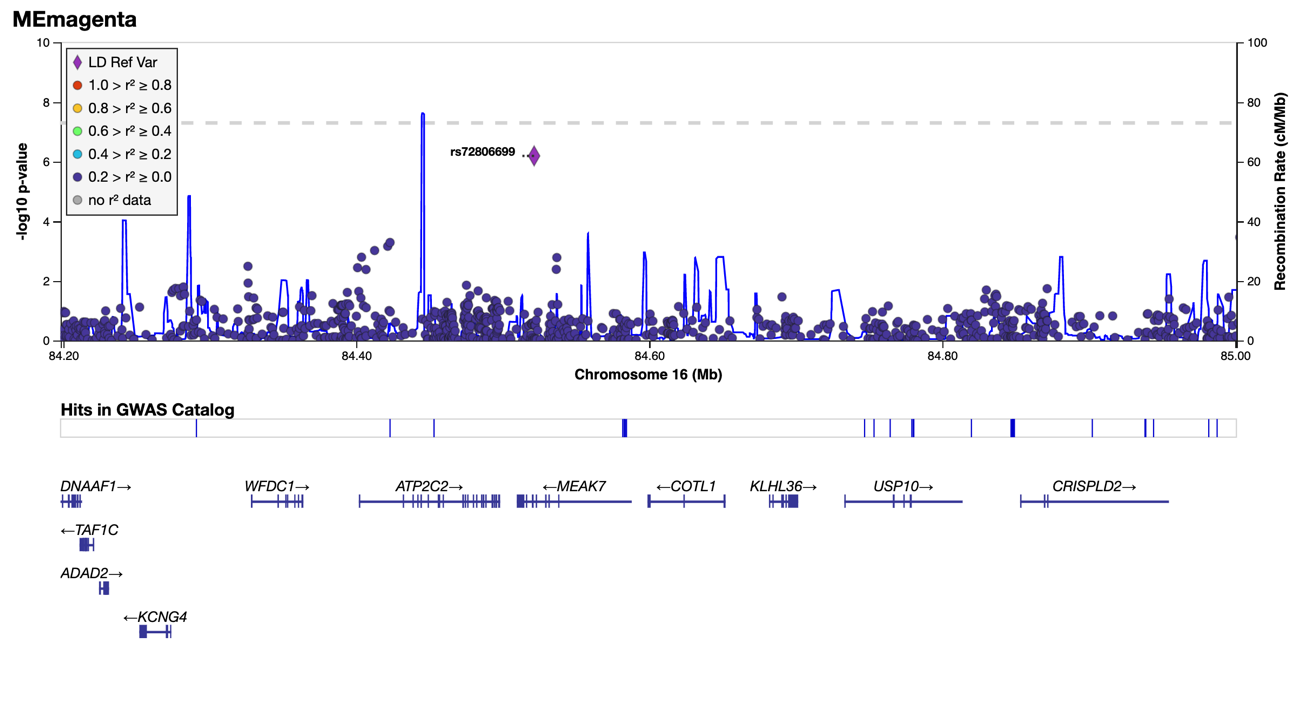


l)


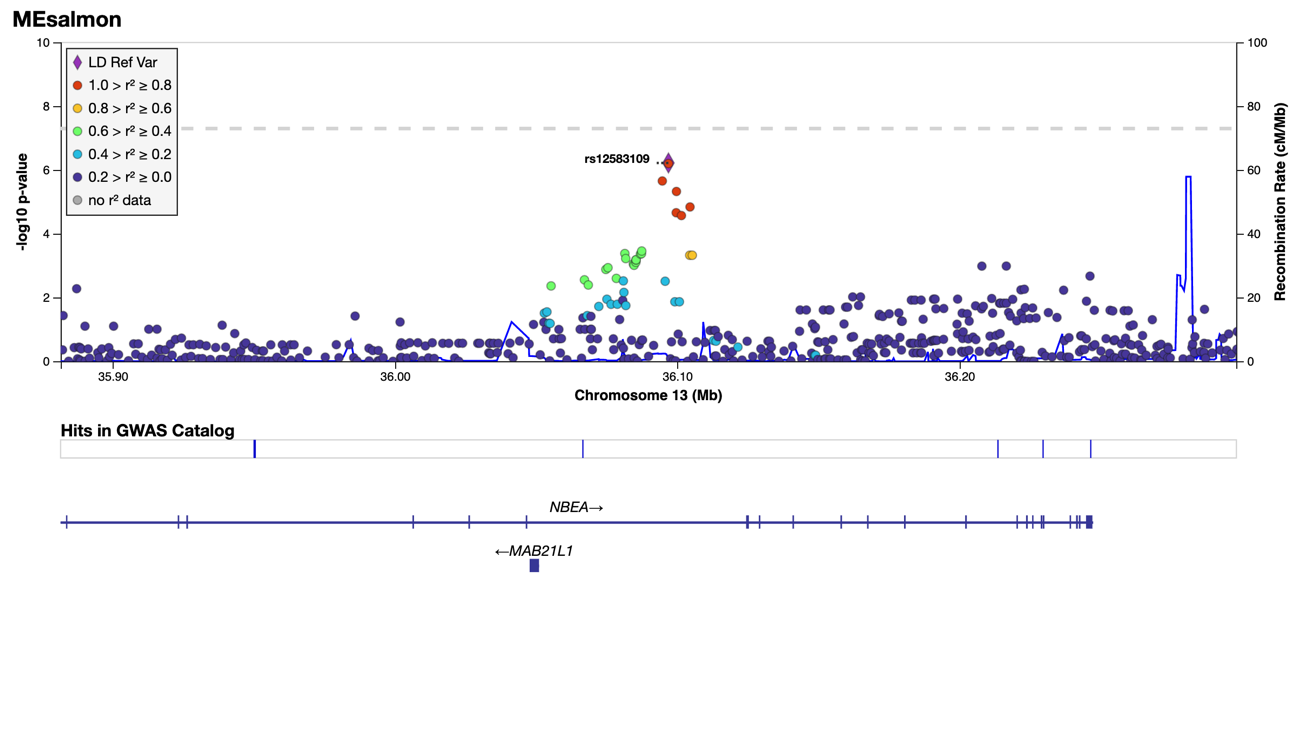


**Supplementary Figure S7. Regional plots of the top hits from the GWAS on module eigengenes**. Top hits from the GWAS on M1 (a-b), M3 (c), M4 (d-e), M5 (f-g), M6 (h-k) and M7 (l) module eigengenes.

**SUPPLEMENTARY TABLES:**

| **Supplementary Table S1. Results from normality tests of module eigengenes** | | | | | |
| --- | --- | --- | --- | --- | --- |
|  |  |  |  |  |  |
| **Module** | **Kolmogorov-Smirnov** | **Kurtosis** | **Skewness** | **INT** |  |
| M1 | 0.5091 | 3.6364 | -0.2476 | - |  |
| M2 | 0.7016 | 3.6941 | -0.0024 | - |  |
| M3 | 0.5846 | 3.0059 | -0.1039 | - |  |
| M4 | 0.5388 | 3.4014 | 0.05 | - |  |
| M5 | **1.10E-06** | **7.1107** | **1.6793** | YES |  |
| M6 | 0.0539 | 3.8996 | -0.4359 | - |  |
| M7 | 0.887 | 3.2765 | 0.0455 | - |  |
| In bold results that overcome the normality thresholds; INT: Rank-based inverse normal transformation | | | | |  |

| **Supplementary Table S9. Enrichment on miRNA target genes among genes in each co-expression module** | | | | | |
| --- | --- | --- | --- | --- | --- |
| Highlighted in green miRNAs which expression is significantly correlated with the module eigengene | | | | | |
|  |  |  |  |  |  |
| **Module M1** |  |  |  |  |  |
| **Gene Set** | **Size** | **Expect** | **Ratio** | **P Value** | **FDR** |
| TGAATGT,MIR-181A,MIR-181B,MIR-181C,MIR-181D | 484 | 44.012 | 1.772 | 2.2E-07 | 2.8E-05 |
| ATCATGA,MIR-433 | 115 | 10.457 | 2.773 | 2.5E-07 | 2.8E-05 |
| ATGTTAA,MIR-302C | 243 | 22.097 | 1.901 | 3.0E-05 | 2.2E-03 |
| CATTTCA,MIR-203 | 287 | 26.098 | 1.763 | 8.8E-05 | 4.2E-03 |
| ATACTGT,MIR-144 | 199 | 18.096 | 1.934 | 9.6E-05 | 4.2E-03 |
| AACTGGA,MIR-145 | 234 | 21.278 | 1.833 | 1.3E-04 | 4.8E-03 |
| CTTGTAT,MIR-381 | 205 | 18.641 | 1.878 | 1.8E-04 | 5.6E-03 |
| ATGCAGT,MIR-217 | 115 | 10.457 | 2.199 | 2.2E-04 | 6.2E-03 |
| GTGTTGA,MIR-505 | 105 | 9.548 | 2.199 | 4.1E-04 | 0.0101 |
| ATGCTGG,MIR-338 | 114 | 10.366 | 2.122 | 5.1E-04 | 0.0113 |
| ACTTTAT,MIR-142-5P | 288 | 26.189 | 1.642 | 7.1E-04 | 0.0143 |
| AAGCAAT,MIR-137 | 223 | 20.278 | 1.726 | 8.9E-04 | 0.0165 |
| TACTTGA,MIR-26A,MIR-26B | 299 | 27.189 | 1.582 | 1.5E-03 | 0.0261 |
| GCACTTT,MIR-17-5P,MIR-20A,MIR-106A,MIR-106B,MIR-20B,MIR-519D | 595 | 54.105 | 1.386 | 1.8E-03 | 0.0274 |
| ATTACAT,MIR-380-3P | 102 | 9.275 | 2.049 | 1.9E-03 | 0.0274 |
| TGCACTT,MIR-519C,MIR-519B,MIR-519A | 448 | 40.738 | 1.448 | 2.0E-03 | 0.0280 |
| TAGGTCA,MIR-192,MIR-215 | 47 | 4.274 | 2.574 | 2.7E-03 | 0.0350 |
| TTGCACT,MIR-130A,MIR-301,MIR-130B | 403 | 36.646 | 1.446 | 3.5E-03 | 0.0415 |
| CTTTGTA,MIR-524 | 433 | 39.374 | 1.422 | 3.9E-03 | 0.0415 |
| TTGGAGA,MIR-515-5P,MIR-519E | 149 | 13.549 | 1.771 | 3.9E-03 | 0.0415 |
| ATGTACA,MIR-493 | 314 | 28.553 | 1.506 | 3.9E-03 | 0.0415 |
| ACCATTT,MIR-522 | 160 | 14.549 | 1.718 | 4.9E-03 | 0.0495 |
| GACTGTT,MIR-212,MIR-132 | 161 | 14.640 | 1.708 | 5.3E-03 | 0.0500 |
| AACTGAC,MIR-223 | 96 | 8.730 | 1.947 | 5.4E-03 | 0.0500 |
|  |  |  |  |  |  |
| **Module M7** |  |  |  |  |  |
| **Gene Set** | **Size** | **Expect** | **Ratio** | **P Value** | **FDR** |
| CTTGTAT,MIR-381 | 205 | 3.199 | 3.751 | 7.8E-05 | 0.0172 |
| CCTGAGT,MIR-510 | 45 | 0.702 | 7.121 | 6.3E-04 | 0.0497 |
| GGGATGC,MIR-324-5P | 49 | 0.765 | 6.539 | 9.4E-04 | 0.0497 |
| AGCGCAG,MIR-191 | 13 | 0.203 | 14.789 | 9.4E-04 | 0.0497 |
| GTAAACC,MIR-299-5P | 51 | 0.796 | 6.283 | 1.1E-03 | 0.0497 |

| **Supplementary Table S10. Results from the correlations between miRNA expression and module eigengene** | | | |
| --- | --- | --- | --- |
| Higlighted in green significant correlations that overcome the Bonferroni correction | | | |
|  |  |  |  |
| **Module M1** |  |  |  |
| **mature miRNA** | **Gene-set** | **rho** | **p-value** |
| hsa-miR-106a-5p | GCACTTT,MIR-106A | -0.0491 | 0.3890 |
| hsa-miR-106b-5p | GCACTTT,MIR-106B | 0.0402 | 0.4808 |
| hsa-miR-130a-3p | TTGCACT,MIR-130A | -0.0200 | 0.7259 |
| hsa-miR-130b-3p | TTGCACT,MIR-130B | -0.0548 | 0.3359 |
| hsa-miR-132-3p | GACTGTT,MIR-132 | 0.1656 | 0.0035 |
| hsa-miR-142-5p | ACTTTAT,MIR-142-5P | 0.2849 | 3.37E-07 |
| hsa-miR-145-5p | AACTGGA,MIR-145 | -0.0863 | 0.1294 |
| hsa-miR-17-5p | GCACTTT,MIR-17-5P | -0.0979 | 0.0854 |
| hsa-miR-181a-5p | TGAATGT,MIR-181A | 0.2819 | 4.52E-07 |
| hsa-miR-181b-5p | TGAATGT,MIR-181B | 0.0947 | 0.0961 |
| hsa-miR-181c-5p | TGAATGT,MIR-181C | 0.1646 | 0.0037 |
| hsa-miR-181d-5p | TGAATGT,MIR-181D | -0.0048 | 0.9325 |
| hsa-miR-192-5p | TAGGTCA,MIR-192 | 0.2897 | 2.09E-07 |
| hsa-miR-20a-5p | GCACTTT,MIR-20A | -0.1098 | 0.0534 |
| hsa-miR-20b-5p | GCACTTT,MIR-20B | -0.0981 | 0.0847 |
| hsa-miR-215-5p | TAGGTCA,MIR-215 | 0.2057 | 2.66E-04 |
| hsa-miR-223-3p | AACTGAC,MIR-223 | -0.1689 | 0.0029 |
| hsa-miR-26a-5p | TACTTGA,MIR-26A | 0.1030 | 0.0701 |
| hsa-miR-26b-5p | TACTTGA,MIR-26B | -0.0271 | 0.6350 |
| hsa-miR-338-3p | ATGCTGG,MIR-338 | 0.0035 | 0.9510 |
| hsa-miR-433-3p | ATCATGA,MIR-433 | -0.0282 | 0.6213 |
| hsa-miR-493-5p | ATGTACA,MIR-493 | -0.0514 | 0.3670 |
| hsa-miR-505-3p | GTGTTGA,MIR-505 | 0.0111 | 0.8457 |
|  |  |  |  |
| **Module M7** |  |  |  |
| **mature miRNA** | **Gene-set** | **rho** | **p-value** |
| hsa-miR-191-3p | AGCGCAG,MIR-191 | 0.0867 | 0.1277 |
| hsa-miR-299-5p | GTAAACC,MIR-299-5P | -0.0799 | 0.1605 |
| hsa-miR-324-5p | GGGATGC,MIR-324-5P | -0.1348 | 0.0176 |
| hsa-miR-381-3p | CTTGTAT,MIR-381 | -0.0446 | 0.4341 |

| **Supplementary Table S11. Results from gene-set analysis using GWAS and EWAS data on ADHD** | | | | | | |
| --- | --- | --- | --- | --- | --- | --- |
|  |  |  |  |  |  |  |
| **Module** | **GWAS gene-set analysis** | |  | **EWAS gene-set analysis** | | |
|  | **MAGMA** | **eMAGMA** |  | **gsameth** | **methylRRA** | **methylglm** |
| M1 | 0.292 | 0.821 |  | 0.88 | 0.615 | 0.323 |
| M2 | 0.795 | 0.752 |  | 0.601 | 0.841 | **5.20E-03** |
| M3 | 0.855 | 0.359 |  | 0.027 | 0.142 | 7.90E-03 |
| M4 | **1.80E-03** | **4.20E-03** |  | **1.60E-04** | **1.00E-03** | **8.20E-05** |
| M5 | 0.52 | 0.739 |  | 0.087 | 0.214 | 0.38 |
| M6 | 0.689 | 0.841 |  | 0.113 | 0.187 | 0.67 |
| M7 | 0.375 | 0.454 |  | 0.015 | 0.857 | 0.177 |
| In bold results that overcome multiple testing correction for each method. Underlined nominal associations. GWAS: Genome-wide association study; EWAS: Epigenome-wide association study; MAGMA: the proximity-based gene mapping method. eMAGMA: eQTL gene mapping based on the whole blood cis-eQTL from GTEx v8. | | | | | | |
