# Supplementary material for: Comprehensive analysis of omics data identifies relevant gene networks for Attention-Deficit/Hyperactivity Disorder (ADHD)
Source: Transl Psychiatry. 2022 Sep 24;12:409. doi: 10.1038/s41398-022-02182-8 (PMC9509350; doi:10.1038/s41398-022-02182-8)
Supplement: Supplementary file 2 — Supplementary tables [file 41398_2022_2182_MOESM2_ESM.pdf]

Supplementary Table S2. Results from the regression analysis to identify co-expression modules associated with ADHD and confounding factors.

	Number	ADHD		Gender		RIN		Microarray batch		Age	
**Module ID**	**of genes**	**effect size**	**p-value**	**effect size**	**p-value**	**effect size**	**p-value**	**effect size**	**p-value**	**effect size**	**p-value**
**M1**	1546	9.5337	**9.28E-06**	-0.7261	0.7205	0.43884	0.615	1.3971	0.4640	-4.0386	0.7120
**M2**	1239	-6.4924	**1.70E-03**	0.4227	0.8348	0.1047	0.904	-0.8271	0.6650	0.6534	0.9520
**M3**	79	7.8721	**1.59E-04**	0.3452	0.8648	0.02632	0.976	0.8483	0.6570	3.6853	0.7360
**M4**	885	-7.8269	**1.83E-04**	1.0067	0.6197	-0.64123	0.462	-0.9483	0.6190	3.5230	0.7470
**M5**	133	-11.1709	**2.59E-06**	-0.0923	0.9637	-1.17389	0.178	-0.3409	0.8580	7.7282	0.4790
**M6**	258	11.5868	**2.04E-07**	0.0682	0.9732	0.0771	0.93	0.8855	0.6430	-3.2804	0.7640
**M7**	185	8.6624	**4.38E-05**	-0.0980	0.9615	0.25624	0.769	-0.1538	0.9360	0.5251	0.9620
**M8**	453	1.9718	0.3260	-	-	-	-	-	-	-	-
**M9**	161	4.4237	0.0296	-	-	-	-	-	-	-	-
**M10**	56	2.3655	0.2400	-	-	-	-	-	-	-	-
**M11**	35	1.3604	0.4970	-	-	-	-	-	-	-	-
**M12**	79	-5.6841	8.54E-03	-	-	-	-	-	-	-	-
**M13**	60	-4.1308	0.0414	-	-	-	-	-	-	-	-
**M14**	223	3.6911	0.0674	-	-	-	-	-	-	-	-
**M15**	124	0.5776	0.7730	-	-	-	-	-	-	-	-
**M16**	115	-1.2796	0.5250	-	-	-	-	-	-	-	-
**M17**	115	-5.6475	5.93E-03	-	-	-	-	-	-	-	-
**M18**	143	-3.5769	0.0766	-	-	-	-	-	-	-	-
**M19**	43	-5.6555	5.70E-03	-	-	-	-	-	-	-	-
**M20**	283	-1.0344	0.6050								
**M21**	234	4.4669	0.0281	-	-	-	-	-	-	-	-
**M22**	792	-0.3459	0.8630	-	-	-	-	-	-	-	-
**M23**	106	5.5036	9.22E-03	-	-	-	-	-	-	-	-
**M24**	203	-4.8706	0.0171	-	-	-	-	-	-	-	-
**M25**	2191	-3.6795	0.0683	-	-	-	-	-	-	-	-
**M26**	33	-2.6450	0.2050	-	-	-	-	-	-	-	-
**M27**	1132	-2.2596	0.2600	-	-	-	-	-	-	-	-
**M0**	8114	-4.2688	0.0358	-	-	-	-	-	-	-	-

*RIN: RNA integrity number

Supplementary Table S3. Gene expression in brain at different developmental stages across ADHD-associated co-expression modules

Module M1

Age_ category	Structure_id	Structure	Main brain structure	n_significant	mean_ FWER	min_ FWER	Equivalent_structures	FWERs
1	Allen:10225	IPC_posteroventral (inferior) parietal cortex	prosencephalon	7	**0.0254**	0	Allen:10225;Allen:10214	0;0;0;0;0;0;0,001;0,119;0,109
1	Allen:10163	M1C_primary motor cortex (area M1, area 4)	telencephalon	8	**0.0394**	0	Allen:10163;Allen:10162	0;0;0;0;0;0;0;0,014;0,341
1	Allen:10161	FCx_frontal neocortex	telencephalon	8	**0.0430**	0	Allen:10161	0,006;0;0;0;0;0;0;0,007;0,374
2	Allen:10657	CBC_cerebellar cortex	rhombencephalon	9	**1.7E-03**	0	Allen:10657;Allen:10656;Allen:10655;Allen:10654;Allen:10653	0;0;0;0;0;0;0;0,005;0,01
3	Allen:10657	CBC_cerebellar cortex	rhombencephalon	8	**0.0394**	0	Allen:10657;Allen:10656;Allen:10655;Allen:10654;Allen:10653	0;0;0;0;0;0;0,002;0,018;0,335
4	Allen:10657	CBC_cerebellar cortex	rhombencephalon	7	**0.0250**	0	Allen:10657;Allen:10656;Allen:10655;Allen:10654;Allen:10653	0;0;0;0;0;0,002;0;0,159;0,064

Module M2

Age_ category	Structure_id	Structure	Main brain structure	n_significant	mean_ FWER	min_ FWER	Equivalent_structures	FWERs
1	Allen:10236	A1C_primary auditory cortex (core)	telencephalon	9	**0**	0	Allen:10236	0;0;0;0;0;0;0;0;0
2	Allen:10236	A1C_primary auditory cortex (core)	telencephalon	9	**0**	0	Allen:10236	0;0;0;0;0;0;0;0;0
3	Allen:10236	A1C_primary auditory cortex (core)	telencephalon	9	**0**	0	Allen:10236	0;0;0;0;0;0;0;0;0

4	Allen:10236	A1C_primary auditory cortex (core)	telencephalon	9	**0**	0	Allen:10236	0;0;0;0;0;0;0;0;0
5	Allen:10236	A1C_primary auditory cortex (core)	telencephalon	9	**0**	0	Allen:10236	0;0;0;0;0;0;0;0;0
1	Allen:10361	AMY_amygdaloid complex	telencephalon	9	**0**	0	Allen:10361	0;0;0;0;0;0;0;0;0
3	Allen:10361	AMY_amygdaloid complex	telencephalon	9	**0**	0	Allen:10361	0;0;0;0;0;0;0;0;0
5	Allen:10361	AMY_amygdaloid complex	telencephalon	9	**5.6E-04**	0	Allen:10361	0,005;0;0;0;0;0;0;0;0
4	Allen:10361	AMY_amygdaloid complex	telencephalon	9	**2.6E-03**	0	Allen:10361	0,023;0;0;0;0;0;0;0;0
2	Allen:10361	AMY_amygdaloid complex	telencephalon	9	**3.4E-03**	0	Allen:10361	0,031;0;0;0;0;0;0;0;0
2	Allen:10657	CBC_cerebellar cortex	rhombencephalon	9	**0**	0	Allen:10657;Allen:10656;Allen:10655;Allen:10654;Allen:10653	0;0;0;0;0;0;0;0;0
3	Allen:10657	CBC_cerebellar cortex	rhombencephalon	9	**0**	0	Allen:10657;Allen:10656;Allen:10655;Allen:10654;Allen:10653	0;0;0;0;0;0;0;0;0
4	Allen:10657	CBC_cerebellar cortex	rhombencephalon	9	**0**	0	Allen:10657;Allen:10656;Allen:10655;Allen:10654;Allen:10653	0;0;0;0;0;0;0;0;0
5	Allen:10657	CBC_cerebellar cortex	rhombencephalon	9	**0**	0	Allen:10657;Allen:10656;Allen:10655;Allen:10654;Allen:10653	0;0;0;0;0;0;0;0;0
1	Allen:10331	CN_cerebral nuclei	telencephalon	9	**2.2E-04**	0	Allen:10331	0,002;0;0;0;0;0;0;0;0
3	Allen:10331	CN_cerebral nuclei	telencephalon	9	**1.2E-03**	0	Allen:10331	0,011;0;0;0;0;0;0;0;0
5	Allen:10331	CN_cerebral nuclei	telencephalon	8	**0.0151**	0	Allen:10331	0,136;0;0;0;0;0;0;0;0

2	Allen:10331	CN_cerebral nuclei	telencephalon	8	**0.0381**	0	Allen:10331	0,342;0,001;0;0;0;0;0;0;0
1	Allen:10173	DFC_dorsolateral prefrontal cortex	telencephalon	9	**0**	0	Allen:10173	0;0;0;0;0;0;0;0;0
2	Allen:10173	DFC_dorsolateral prefrontal cortex	telencephalon	9	**0**	0	Allen:10173	0;0;0;0;0;0;0;0;0
4	Allen:10173	DFC_dorsolateral prefrontal cortex	telencephalon	9	**4.4E-04**	0	Allen:10173	0,004;0;0;0;0;0;0;0;0
5	Allen:10173	DFC_dorsolateral prefrontal cortex	telencephalon	9	**2.3E-03**	0	Allen:10173	0,002;0;0;0;0;0;0;0;0,019
1	Allen:13322	DLTC_dorsolateral temporal neocortex	telencephalon	9	**0**	0	Allen:13322	0;0;0;0;0;0;0;0;0
3	Allen:13322	DLTC_dorsolateral temporal neocortex	telencephalon	9	**0**	0	Allen:13322	0;0;0;0;0;0;0;0;0
2	Allen:13322	DLTC_dorsolateral temporal neocortex	telencephalon	9	**1.1E-04**	0	Allen:13322	0,001;0;0;0;0;0;0;0;0
5	Allen:13322	DLTC_dorsolateral temporal neocortex	telencephalon	9	**1.7E-03**	0	Allen:13322	0,015;0;0;0;0;0;0;0;0
4	Allen:13322	DLTC_dorsolateral temporal neocortex	telencephalon	8	**7.0E-03**	0	Allen:13322	0,062;0;0;0;0;0;0;0;0,001
1	Allen:10161	FCx_frontal neocortex	telencephalon	8	**0.0136**	0	Allen:10161	0,12;0;0;0;0;0;0;0;0,002
5	Allen:10161	FCx_frontal neocortex	telencephalon	8	**0.0210**	0	Allen:10161	0,188;0;0;0;0;0;0;0;0,001
1	Allen:10294	HIP_hippocampus (hippocampal formation)	telencephalon	9	**0**	0	Allen:10294;Allen:10293;Allen:10292	0;0;0;0;0;0;0;0;0
3	Allen:10294	HIP_hippocampus (hippocampal formation)	telencephalon	9	**0**	0	Allen:10294;Allen:10293;Allen:10292	0;0;0;0;0;0;0;0;0
2	Allen:10294	HIP_hippocampus (hippocampal formation)	telencephalon	9	**3.3E-04**	0	Allen:10294;Allen:10293;Allen:10292	0,003;0;0;0;0;0;0;0;0
4	Allen:10294	HIP_hippocampus (hippocampal formation)	telencephalon	9	**4.4E-04**	0	Allen:10294;Allen:10293;Allen:10292	0,004;0;0;0;0;0;0;0;0

5	Allen:10294	HIP_hippocampus (hippocampal formation)	telencephalon	9	**6.7E-04**	0	Allen:10294;Allen:10293;Allen:10292	0,006;0;0;0;0;0;0;0;0
2	Allen:10225	IPC_posteroventral (inferior) parietal cortex	telencephalon	9	**0**	0	Allen:10225;Allen:10214	0;0;0;0;0;0;0;0;0
3	Allen:10225	IPC_posteroventral (inferior) parietal cortex	telencephalon	9	**0**	0	Allen:10225;Allen:10214	0;0;0;0;0;0;0;0;0
4	Allen:10225	IPC_posteroventral (inferior) parietal cortex	telencephalon	9	**2.2E-04**	0	Allen:10225;Allen:10214	0;0;0;0;0;0;0;0;0,002
1	Allen:10225	IPC_posteroventral (inferior) parietal cortex	telencephalon	9	**1.3E-03**	0	Allen:10225;Allen:10214	0;0;0;0;0;0;0;0;0,012
5	Allen:10225	IPC_posteroventral (inferior) parietal cortex	telencephalon	8	**0.0228**	0	Allen:10225;Allen:10214	0,001;0;0;0;0;0;0;0;0,204
1	Allen:10252	ITC_inferolateral temporal cortex (area TEv, area 20)	telencephalon	9	**0**	0	Allen:13324;Allen:10252	0;0;0;0;0;0;0;0;0
2	Allen:10252	ITC_inferolateral temporal cortex (area TEv, area 20)	telencephalon	9	**0**	0	Allen:13324;Allen:10252	0;0;0;0;0;0;0;0;0
3	Allen:10252	ITC_inferolateral temporal cortex (area TEv, area 20)	telencephalon	9	**0**	0	Allen:13324;Allen:10252	0;0;0;0;0;0;0;0;0
4	Allen:10252	ITC_inferolateral temporal cortex (area TEv, area 20)	telencephalon	9	**2.2E-04**	0	Allen:13324;Allen:10252	0,002;0;0;0;0;0;0;0;0
5	Allen:10252	ITC_inferolateral temporal cortex (area TEv, area 20)	telencephalon	8	**0.0149**	0	Allen:13324;Allen:10252	0;0;0;0;0;0;0;0;0,134
3	Allen:10163	M1C_primary motor cortex (area M1, area 4)	telencephalon	9	**0**	0	Allen:10163;Allen:10162	0;0;0;0;0;0;0;0;0
4	Allen:10163	M1C_primary motor cortex (area M1, area 4)	telencephalon	9	**0**	0	Allen:10163;Allen:10162	0;0;0;0;0;0;0;0;0

5	Allen:10163	M1C_primary motor cortex (area M1, area 4)	telencephalon	9	**4.4E-04**	0	Allen:10163;Allen:10162	0,004;0;0;0;0;0;0;0;0
1	Allen:10163	M1C_primary motor cortex (area M1, area 4)	telencephalon	8	**8.3E-03**	0	Allen:10163;Allen:10162	0;0;0;0;0;0;0;0;0,075
1	Allen:10398	MD_mediodorsal nucleus of thalamus	dienchephalon	9	**0**	0	Allen:10398;Allen:10397;Allen:10391;Allen:10390;Allen:10389	0;0;0;0;0;0;0;0;0
2	Allen:10398	MD_mediodorsal nucleus of thalamus	dienchephalon	9	**0**	0	Allen:10398;Allen:10397;Allen:10391;Allen:10390;Allen:10389	0;0;0;0;0;0;0;0;0
3	Allen:10398	MD_mediodorsal nucleus of thalamus	dienchephalon	9	**0**	0	Allen:10398;Allen:10397;Allen:10391;Allen:10390;Allen:10389	0;0;0;0;0;0;0;0;0
5	Allen:10398	MD_mediodorsal nucleus of thalamus	dienchephalon	9	**0**	0	Allen:10398;Allen:10397;Allen:10391;Allen:10390;Allen:10389	0;0;0;0;0;0;0;0;0
4	Allen:10398	MD_mediodorsal nucleus of thalamus	dienchephalon	9	**4.4E-04**	0	Allen:10398;Allen:10397;Allen:10391;Allen:10390;Allen:10389	0,004;0;0;0;0;0;0;0;0
1	Allen:10278	MFC_anterior (rostral) cingulate (medial prefrontal) cortex	telencephalon	9	**0**	0	Allen:10278;Allen:10277	0;0;0;0;0;0;0;0;0
2	Allen:10278	MFC_anterior (rostral) cingulate (medial prefrontal) cortex	telencephalon	9	**0**	0	Allen:10278;Allen:10277	0;0;0;0;0;0;0;0;0
4	Allen:10278	MFC_anterior (rostral) cingulate (medial prefrontal) cortex	telencephalon	9	**2.2E-04**	0	Allen:10278;Allen:10277	0,002;0;0;0;0;0;0;0;0
5	Allen:10278	MFC_anterior (rostral) cingulate (medial prefrontal) cortex	telencephalon	8	**0.0392**	0	Allen:10278;Allen:10277	0,024;0;0;0;0;0;0;0;0,329

1	Allen:10194	OFC_orbital frontal cortex	telencephalon	9	**0**	0	Allen:10194	0;0;0;0;0;0;0;0;0
3	Allen:10194	OFC_orbital frontal cortex	telencephalon	9	**0**	0	Allen:10194	0;0;0;0;0;0;0;0;0
4	Allen:10194	OFC_orbital frontal cortex	telencephalon	9	**0**	0	Allen:10194	0;0;0;0;0;0;0;0;0
2	Allen:10194	OFC_orbital frontal cortex	telencephalon	9	**1.1E-04**	0	Allen:10194	0,001;0;0;0;0;0;0;0;0
5	Allen:10194	OFC_orbital frontal cortex	telencephalon	8	**0.0114**	0	Allen:10194	0;0;0;0;0;0;0;0;0,103
2	Allen:10208	PCx_parietal neocortex	telencephalon	9	**0**	0	Allen:10208	0;0;0;0;0;0;0;0;0
3	Allen:10208	PCx_parietal neocortex	telencephalon	9	**0**	0	Allen:10208	0;0;0;0;0;0;0;0;0
4	Allen:10208	PCx_parietal neocortex	telencephalon	9	**2.3E-03**	0	Allen:10208	0,021;0;0;0;0;0;0;0;0
1	Allen:10208	PCx_parietal neocortex	telencephalon	8	**8.4E-03**	0	Allen:10208	0;0;0;0;0;0;0;0;0,076
5	Allen:10208	PCx_parietal neocortex	telencephalon	8	**9.8E-03**	0	Allen:10208	0,08;0;0;0;0;0;0;0;0,008
1	Allen:10172	PFC_prefrontal cortex	telencephalon	9	**0**	0	Allen:10172	0;0;0;0;0;0;0;0;0
2	Allen:10172	PFC_prefrontal cortex	telencephalon	9	**3.3E-04**	0	Allen:10172	0,003;0;0;0;0;0;0;0;0
4	Allen:10172	PFC_prefrontal cortex	telencephalon	8	**0.0217**	0	Allen:10172	0,195;0;0;0;0;0;0;0;0
2	Allen:10209	S1C_primary somatosensory cortex (area S1, areas 3,1,2)	telencephalon	9	**0**	0	Allen:10209	0;0;0;0;0;0;0;0;0
3	Allen:10209	S1C_primary somatosensory cortex (area S1, areas 3,1,2)	telencephalon	9	**0**	0	Allen:10209	0;0;0;0;0;0;0;0;0
4	Allen:10209	S1C_primary somatosensory cortex (area S1, areas 3,1,2)	telencephalon	9	**0**	0	Allen:10209	0;0;0;0;0;0;0;0;0

5	Allen:10209	S1C_primary somatosensory cortex (area S1, areas 3,1,2)	telencephalon	9	**4.4E-04**	0	Allen:10209	0,004;0;0;0;0;0;0;0;0
1	Allen:10209	S1C_primary somatosensory cortex (area S1, areas 3,1,2)	telencephalon	9	**4.8E-03**	0	Allen:10209	0;0;0;0;0;0;0;0;0,043
1	Allen:10243	STC_posterior (caudal) superior temporal cortex (area 22c)	telencephalon	9	**0**	0	Allen:10243;Allen:10240	0;0;0;0;0;0;0;0;0
2	Allen:10243	STC_posterior (caudal) superior temporal cortex (area 22c)	telencephalon	9	**0**	0	Allen:10243;Allen:10240	0;0;0;0;0;0;0;0;0
3	Allen:10243	STC_posterior (caudal) superior temporal cortex (area 22c)	telencephalon	9	**0**	0	Allen:10243;Allen:10240	0;0;0;0;0;0;0;0;0
4	Allen:10243	STC_posterior (caudal) superior temporal cortex (area 22c)	telencephalon	9	**2.2E-04**	0	Allen:10243;Allen:10240	0,002;0;0;0;0;0;0;0;0
5	Allen:10243	STC_posterior (caudal) superior temporal cortex (area 22c)	telencephalon	9	**5.6E-04**	0	Allen:10243;Allen:10240	0,004;0;0;0;0;0;0;0;0,001
1	Allen:10333	STR_striatum	telencephalon	9	**0**	0	Allen:10333;Allen:10332	0;0;0;0;0;0;0;0;0
2	Allen:10333	STR_striatum	telencephalon	9	**0**	0	Allen:10333;Allen:10332	0;0;0;0;0;0;0;0;0
3	Allen:10333	STR_striatum	telencephalon	9	**0**	0	Allen:10333;Allen:10332	0;0;0;0;0;0;0;0;0
5	Allen:10333	STR_striatum	telencephalon	9	**1.1E-04**	0	Allen:10333;Allen:10332	0,001;0;0;0;0;0;0;0;0
4	Allen:10333	STR_striatum	telencephalon	9	**2.2E-04**	0	Allen:10333;Allen:10332	0;0;0;0;0;0;0;0;0,002
1	Allen:10235	TCx_temporal neocortex	telencephalon	9	**0**	0	Allen:10235	0;0;0;0;0;0;0;0;0
3	Allen:10235	TCx_temporal neocortex	telencephalon	9	**0**	0	Allen:10235	0;0;0;0;0;0;0;0;0
2	Allen:10235	TCx_temporal neocortex	telencephalon	9	**7.8E-04**	0	Allen:10235	0,007;0;0;0;0;0;0;0;0

5	Allen:10235	TCx_temporal neocortex	telencephalon	8	**0.0113**	0	Allen:10235	0,092;0;0;0;0;0;0;0;0,01
1	Allen:10269	V1C_primary visual cortex (striate cortex, area V1/17)	telencephalon	9	**0**	0	Allen:10269;Allen:10268	0;0;0;0;0;0;0;0;0
2	Allen:10269	V1C_primary visual cortex (striate cortex, area V1/17)	telencephalon	9	**0**	0	Allen:10269;Allen:10268	0;0;0;0;0;0;0;0;0
3	Allen:10269	V1C_primary visual cortex (striate cortex, area V1/17)	telencephalon	9	**0**	0	Allen:10269;Allen:10268	0;0;0;0;0;0;0;0;0
4	Allen:10269	V1C_primary visual cortex (striate cortex, area V1/17)	telencephalon	9	**1.1E-04**	0	Allen:10269;Allen:10268	0,001;0;0;0;0;0;0;0;0
5	Allen:10269	V1C_primary visual cortex (striate cortex, area V1/17)	telencephalon	9	**7.8E-04**	0	Allen:10269;Allen:10268	0;0;0;0;0;0;0;0;0,007
1	Allen:10185	VFC_ventrolateral prefrontal cortex	telencephalon	9	**0**	0	Allen:10185	0;0;0;0;0;0;0;0;0
2	Allen:10185	VFC_ventrolateral prefrontal cortex	telencephalon	9	**0**	0	Allen:10185	0;0;0;0;0;0;0;0;0
3	Allen:10185	VFC_ventrolateral prefrontal cortex	telencephalon	9	**0**	0	Allen:10185	0;0;0;0;0;0;0;0;0
4	Allen:10185	VFC_ventrolateral prefrontal cortex	telencephalon	9	**4.4E-04**	0	Allen:10185	0,004;0;0;0;0;0;0;0;0
5	Allen:10185	VFC_ventrolateral prefrontal cortex	telencephalon	9	**3.8E-03**	0	Allen:10185	0,001;0;0;0;0;0;0;0;0,033

Module M4

Age_category	Structure_id	Structure	Main brain structure	n_significant	mean_FWER	min_FWER	Equivalent_structures	FWERs

1	Allen:10269	V1C_primary visual cortex (striate cortex, area V1/17)	telencephalon	9	**0**	0	Allen:10269;Allen:10268	0;0;0;0;0;0;0;0;0
1	Allen:10243	STC_posterior (caudal) superior temporal cortex (area 22c)	telencephalon	9	**3.3E-04**	0	Allen:10243;Allen:10240	0;0;0;0;0;0;0;0;0.003
1	Allen:10185	VFC_ventrolateral prefrontal cortex	telencephalon	9	**3.4E-03**	0	Allen:10185	0;0;0;0;0;0;0;0.002;0.029
1	Allen:13322	DLTC_dorsolateral temporal neocortex	telencephalon	9	**5.4E-03**	0	Allen:13322	0;0;0;0;0;0;0;0.002;0.047
1	Allen:10235	TCx_temporal neocortex	telencephalon	8	**8.9E-03**	0	Allen:10235	0.005;0;0;0;0;0;0;0.006;0.069
1	Allen:10172	PFC_prefrontal cortex	telencephalon	8	**0.0133**	0	Allen:10172	0;0;0;0;0;0;0.006;0.006;0.108
1	Allen:10333	STR_striatum	telencephalon	8	**0.0304**	0	Allen:10333;Allen:10332	0;0;0;0;0;0;0.001;0.002;0.271
1	Allen:10278	MFC_anterior (rostral) cingulate (medial prefrontal) cortex	telencephalon	8	**0.0384**	0	Allen:10278;Allen:10277	0;0;0;0;0;0;0.005;0.002;0.339
1	Allen:10331	CN_cerebral nuclei	telencephalon	8	**0.0391**	0	Allen:10331	0.006;0.003;0;0;0;0;0.012;0.03;0.301
1	Allen:10173	DFC_dorsolateral prefrontal cortex	telencephalon	8	**0.0466**	0	Allen:10173	0;0;0;0;0;0;0.02;0.019;0.38
2	Allen:10657	CBC_cerebellar cortex	rhombencephalon	9	**2.6E-03**	0	Allen:10657;Allen:10656;Allen:10655;Allen:10654;Allen:10653	0;0;0;0;0;0;0;0;0.023
2	Allen:10361	AMY_amygdaloid complex	telencephalon	8	**0.0302**	0	Allen:10361	0;0;0;0;0;0;0;0;0.272
3	Allen:10657	CBC_cerebellar cortex	rhombencephalon	9	**1.1E-04**	0	Allen:10657;Allen:10656;Allen:10655;Allen:10654;Allen:10653	0;0;0;0;0;0;0;0;0.001

4	Allen:10398	MD_mediodorsal nucleus of thalamus	dienchephalon	9	**7.8E-04**	0	Allen:10398;Allen:10397;Allen:10391;Allen:10390;Allen:10389	0;0;0;0;0;0;0;0;0.007
4	Allen:10657	CBC_cerebellar cortex	rhombencephalon	8	**6.4E-03**	0	Allen:10657;Allen:10656;Allen:10655;Allen:10654;Allen:10653	0;0;0;0;0;0;0.001;0;0.057
4	Allen:10294	HIP_hippocampus (hippocampal formation)	telencephalon	8	**0.0321**	0	Allen:10294;Allen:10293;Allen:10292	0;0;0;0;0;0;0;0;0.289
4	Allen:10194	OFC_orbital frontal cortex	telencephalon	8	**0.0460**	0	Allen:10194	0;0;0;0;0;0;0;0.001;0.413
5	Allen:10657	CBC_cerebellar cortex	rhombencephalon	8	**0.0128**	0	Allen:10657;Allen:10656;Allen:10655;Allen:10654;Allen:10653	0;0;0;0;0;0;0;0;0.115

Module M7

Age_category	Structure_id	Structure	Main brain structure	n_significant	mean_FWER	min_FWER	Equivalent_structures	FWERs
1	Allen:10163	M1C_primary motor cortex (area M1, area 4)	telencephalon	7	**0.0360**	0	Allen:10163;Allen:10162	0.009;0.003;0;0;0;0.004;0.018;0.105;0.185

Column1 shows the classification of ages from the Developing Human Brain Atlas into five developmental stages: 1= prenatal; 2= 0-2 yrs; 3=3-11 yrs; 4= 12-19yrs;

Supplementry Table S4. Enrichment analysis of genes in each co-expression module in Gene Ontology Biological Processes

Highlighted in green relevant pathways previously related with psychiatric disorders

Module M1

Description	Gene set	Size	Expect	Ratio	P Value	FDR
mRNA processing	GO:0006397	487	37.752	2.437	4.44E-16	3.77E-13
ncRNA processing	GO:0034470	367	28.450	2.320	7.72E-11	2.19E-08
regulation of chromosome organization	GO:0033044	329	25.504	2.157	4.20E-08	5.95E-06
mitotic cell cycle phase transition	GO:0044772	487	37.752	1.854	3.15E-07	2.67E-05
methylation	GO:0032259	346	26.822	1.864	1.32E-05	7.03E-04
protein polyubiquitination	GO:0000209	255	19.768	2.024	1.41E-05	7.06E-04
protein modification by small protein removal	GO:0070646	288	22.326	1.926	2.37E-05	9.60E-04
nucleic acid phosphodiester bond hydrolysis	GO:0090305	288	22.326	1.926	2.37E-05	9.60E-04
cilium organization	GO:0044782	409	31.706	1.735	4.03E-05	1.43E-03
posttranscriptional regulation of gene expression	GO:0010608	486	37.675	1.646	6.55E-05	2.03E-03

Module M2

Description	Gene set	Size	Expect	Ratio	P Value	FDR
ribonucleoprotein complex biogenesis	GO:0022613	440	28.048	2.852	0	0
translational elongation	GO:0006414	133	8.478	4.836	0	0
protein targeting	GO:0006605	412	26.263	2.589	2.97E-13	6.31E-11
nucleoside triphosphate metabolic process	GO:0009141	304	19.378	2.838	1.42E-12	2.42E-10
protein-containing complex disassembly	GO:0032984	323	20.590	2.623	5.41E-11	4.91E-09
generation of precursor metabolites and energy	GO:0006091	469	29.896	2.107	1.32E-08	7.01E-07
mRNA processing	GO:0006397	487	31.044	2.029	5.52E-08	2.61E-06
response to endoplasmic reticulum stress	GO:0034976	268	17.084	2.283	1.09E-06	4.04E-05
proteasomal protein catabolic process	GO:0010498	449	28.622	1.782	4.13E-05	0.001032
process utilizing autophagic mechanism	GO:0061919	473	30.151	1.692	1.56E-04	0.003157

Module M4

Description	Gene set	Size	Expect	Ratio	P Value	FDR
covalent chromatin modification	GO:0016569	468	22.248	2.517	1.59E-10	1.35E-07
peptidyl-lysine modification	GO:0018205	360	17.114	2.162	8.59E-06	3.21E-03
RNA 3'-end processing	GO:0031123	141	6.703	2.984	1.13E-05	3.21E-03
regulation of gene expression, epigenetic	GO:0040029	258	12.265	2.365	1.52E-05	3.24E-03
proteasomal protein catabolic process	GO:0010498	449	21.345	1.921	4.77E-05	8.11E-03
beta-catenin-TCF complex assembly	GO:1904837	30	1.426	5.610	5.77E-05	8.18E-03
protein alkylation	GO:0008213	174	8.272	2.418	2.31E-04	0.02802
positive regulation of catabolic process	GO:0009896	409	19.443	1.852	2.80E-04	0.02976
regulation of binding	GO:0051098	367	17.447	1.892	3.39E-04	0.03168
negative regulation of phosphorylation	GO:0042326	423	20.109	1.790	5.28E-04	0.04084

Module M5

Description	Gene set	Size	Expect	Ratio	P Value	FDR
response to molecule of bacterial origin	GO:0002237	330	2.273	11.879	0	0
leukocyte differentiation	GO:0002521	496	3.416	6.147	1.82E-11	5.16E-09
cell chemotaxis	GO:0060326	289	1.991	8.038	1.23E-10	2.05E-08
response to peptide	GO:1901652	487	3.354	5.366	5.22E-09	4.43E-07
regulation of DNA-binding transcription factor activity	GO:0051090	404	2.783	5.750	1.56E-08	1.06E-06
regulation of peptide secretion	GO:0002791	463	3.189	5.331	1.61E-08	1.06E-06
positive regulation of cell motility	GO:2000147	493	3.396	4.712	2.42E-07	1.08E-05
negative regulation of intracellular signal transduction	GO:1902532	495	3.409	4.693	2.55E-07	1.09E-05
rhythmic process	GO:0048511	279	1.922	5.724	3.40E-06	6.73E-05
positive regulation of proteolysis	GO:0045862	343	2.363	4.656	2.38E-05	3.27E-04

Module M6

Description	Gene set	Size	Expect	Ratio	P Value	FDR
mRNA processing	GO:0006397	487	5.623	3.379	3.58E-06	3.04E-03

Module M7

Description	Gene set	Size	Expect	Ratio	P Value	FDR
RNA catabolic process	GO:0006401	341	2.947	4.071	3.97E-05	0.016803
nuclear transport	GO:0051169	356	3.077	3.900	6.02E-05	0.016803
posttranscriptional regulation of gene expression	GO:0010608	486	4.201	3.333	7.91E-05	0.016803
signal transduction by p53 class mediator	GO:0072331	218	1.884	4.776	1.15E-04	0.019612
cell cycle arrest	GO:0007050	240	2.074	4.339	2.37E-04	0.033639

Supplementry Table S5. Enrichment analysis of genes in each co-expression module in REACTOME.
Highlighted in green relevant pathways previously related with psychiatric disorders

Module M1

Description	Gene Set	Size	Expect	Ratio	P Value	FDR
Gene expression (Transcription)	R-HSA-74160	1430	111.380	1.715	2.66E-15	4.60E-12
Metabolism of RNA	R-HSA-8953854	674	52.495	1.962	9.34E-12	5.38E-09
Organelle biogenesis and maintenance	R-HSA-1852241	294	22.898	1.922	1.85E-05	3.23E-03
Class C/3 (Metabotropic glutamate/pheromone receptors)	R-HSA-420499	38	2.960	4.055	1.87E-05	3.23E-03
DNA Repair	R-HSA-73894	316	24.612	1.828	5.22E-05	7.47E-03
SUMOylation of DNA damage response and repair proteins	R-HSA-3108214	76	5.919	2.703	2.02E-04	0.0233
Signaling by cytosolic FGFR1 fusion mutants	R-HSA-1839117	18	1.402	4.993	2.51E-04	0.0255
Antigen processing: Ubiquitination & Proteasome degradation	R-HSA-983168	309	24.067	1.704	5.09E-04	0.0382
HIV Life Cycle	R-HSA-162587	151	11.761	2.041	6.00E-04	0.0414

Module M2

Description	Gene Set	Size	Expect	Ratio	P Value	FDR
Metabolism of RNA	R-HSA-8953854	674	46.619	2.553	0.00E+00	0.00E+00
Translation	R-HSA-72766	291	20.128	4.024	0.00E+00	0.00E+00
Respiratory electron transport, ATP synthesis by chemiosmotic coupling, and heat production by uncoupling proteins.	R-HSA-163200	123	8.508	3.761	3.17E-11	2.38E-09
Metabolism of amino acids and derivatives	R-HSA-71291	370	25.592	2.384	1.19E-10	7.91E-09
Cellular responses to external stimuli	R-HSA-8953897	503	34.792	1.955	5.24E-08	1.86E-06
PTEN Regulation	R-HSA-6807070	140	9.684	2.995	7.20E-08	2.44E-06
Disease	R-HSA-1643685	1054	72.903	1.577	2.88E-07	7.53E-06
Mitochondrial protein import	R-HSA-1268020	64	4.427	3.840	9.97E-07	1.87E-05
DNA Repair	R-HSA-73894	316	21.857	2.105	1.06E-06	1.92E-05
Gluconeogenesis	R-HSA-70263	34	2.352	5.103	1.46E-06	2.54E-05

Module M3

Description	Gene Set	Size	Expect	Ratio	P Value	FDR
CTLA4 inhibitory signaling	R-HSA-389513	21	0.060	50.257	2.66E-05	0.0460

Module M4

Description	Gene Set	Size	Expect	Ratio	P Value	FDR
SUMOylation of transcription cofactors	R-HSA-3899300	45	2.136	5.149	5.77E-06	9.97E-03

Module M5

Description	Gene Set	Size	Expect	Ratio	P Value	FDR
Signaling by Interleukins	R-HSA-449147	462	3.546	6.487	2.94E-13	2.54E-10
Immune System	R-HSA-168256	1997	15.327	2.349	1.18E-07	4.07E-05
TP53 regulates transcription of additional cell cycle genes whose exact role in the p53 pathway remain uncertain	R-HSA-6804115	21	0.161	18.614	5.24E-04	0.0302
RAF-independent MAPK1/3 activation	R-HSA-112409	23	0.177	16.995	6.90E-04	0.0376
Signaling by GPCR	R-HSA-372790	1162	8.918	2.131	1.03E-03	0.0495

Module M7

Description	Gene Set	Size	Expect	Ratio	P Value	FDR
Gene expression (Transcription)	R-HSA-74160	1430	12.194	2.542	3.61E-07	6.24E-04
Chromatin modifying enzymes	R-HSA-3247509	275	2.345	4.264	1.12E-04	0.0386

Supplementry Table S6. Enrichment analysis of genes in each co-expression module in KEGG pathways

Highlighted in green relevant pathways previously related with psychiatric disorders

Module M1

Description	Gene Set	Size	Expect	Ratio	P Value	FDR
RNA degradation	hsa03018	79	5.045	4.162	1.21E-08	3.96E-06
Ubiquitin mediated proteolysis	hsa04120	137	8.749	2.629	1.60E-05	2.60E-03
Spliceosome	hsa03040	134	8.558	2.571	3.49E-05	3.79E-03

Module M2

Description	Gene Set	Size	Expect	Ratio	P Value	FDR
Ribosome	hsa03010	153	10.550	3.792	6.00E-14	1.95E-11
Huntington disease	hsa05016	193	13.308	3.306	6.12E-13	9.98E-11
Alzheimer disease	hsa05010	171	11.791	2.884	1.27E-08	8.28E-07
Thermogenesis	hsa04714	229	15.790	2.470	9.44E-08	5.13E-06
Proteasome	hsa03050	45	3.103	4.834	1.54E-07	7.19E-06
Metabolic pathways	hsa01100	1305	89.982	1.478	5.26E-07	2.14E-05
Non-alcoholic fatty liver disease (NAFLD)	hsa04932	149	10.274	2.725	8.48E-07	3.07E-05
RNA transport	hsa03013	171	11.791	2.036	6.28E-04	0.0137
Spliceosome	hsa03040	134	9.240	2.165	8.02E-04	0.0163
Protein processing in endoplasmic reticulum	hsa04141	165	11.377	2.022	8.92E-04	0.0171

Module M4

Description	Gene Set	Size	Expect	Ratio	P Value	FDR
Insulin signaling pathway	hsa04910	137	5.815	3.268	4.54E-06	1.48E-03
Longevity regulating pathway	hsa04211	89	3.777	3.706	1.97E-05	3.21E-03
Phospholipase D signaling pathway	hsa04072	146	6.197	2.905	4.16E-05	4.52E-03
Apelin signaling pathway	hsa04371	137	5.815	2.924	6.26E-05	5.10E-03
Endocytosis	hsa04144	244	10.356	2.221	2.59E-04	0.0121
Human papillomavirus infection	hsa05165	339	14.388	1.946	5.18E-04	0.0188
Cushing syndrome	hsa04934	154	6.536	2.448	7.92E-04	0.0244
Autophagy	hsa04140	128	5.433	2.577	1.01E-03	0.0274
Human T-cell leukemia virus 1 infection	hsa05166	255	10.823	2.033	1.17E-03	0.0293
Lysosome	hsa04142	123	5.220	2.490	2.07E-03	0.0374

Module M5

Description	Gene Set	Size	Expect	Ratio	P Value	FDR
IL-17 signaling pathway	hsa04657	93	0.859	17.459	2.66E-15	8.69E-13
TNF signaling pathway	hsa04668	110	1.016	14.761	3.67E-14	5.99E-12
NF-kappa B signaling pathway	hsa04064	95	0.878	13.673	4.28E-11	3.96E-09
Legionellosis	hsa05134	55	0.508	19.681	4.85E-11	3.96E-09
NOD-like receptor signaling pathway	hsa04621	168	1.552	9.021	2.61E-10	1.42E-08
C-type lectin receptor signaling pathway	hsa04625	104	0.961	11.449	2.08E-09	8.47E-08
Cytokine-cytokine receptor interaction	hsa04060	294	2.716	5.155	3.45E-07	9.34E-06
MAPK signaling pathway	hsa04010	295	2.725	4.403	1.35E-05	2.33E-04
Human T-cell leukemia virus 1 infection	hsa05166	255	2.356	4.670	1.87E-05	3.04E-04
cAMP signaling pathway	hsa04024	199	1.838	3.808	2.29E-03	0.0162

Supplementary Table S7. Enrichment analysis on druggable genes in each co-expression module using data from PharmaKG.
FDR: False Discovery Rate; ATC: Anatomical Therapeutic Chemical; np: Information not present in the ATC.

Module M1

Description	Gene Set	Size	Expect	Ratio	P Value	FDR	ATC5	ATC4	DEFINITION	ATC3	DEFINITION	ATC2	DEFINITION	ATC1	DEFINITION
threonine	PA451673	875	52.880	1.853	1.49E-09	1.37E-06	np	np	np	np	np	np	np	np	np
serine	PA451330	993	60.011	1.683	1.22E-07	7.45E-05	np	np	np	np	np	np	np	np	np
hydroxyurea	PA449942	96	5.802	2.930	5.58E-05	0.0117	np	np	np	np	np	np	np	np	np
zinc	PA451956	776	46.897	1.919	1.30E-09	1.37E-06	A12CB	np	np	A12C	OTHER MINERALS	A12	MINERALS	A.	ALIMENTARY SYSTEM AND METABOLISM
citric acid	PA449021	97	5.862	2.900	6.40E-05	0.0118	A09AB04	A09AB	Acid-containing	A09A	DIGESTIVA, INCL. ENZYMES	A09	DIGESTIVA, INCL. ENZYMES	A.	ALIMENTARY SYSTEM AND METABOLISM
mitomycin	PA450524	102	6.164	3.082	9.80E-06	4.50E-03	L01DC03	L01DC	Other cytotoxic antibiotics	L01D	CYTOTOXIC ANTIBIOTICS AND RELATED SUBSTANCES	L01	ANTINEOPLASTIC AGENTS	L.	ANTINEOPLASTIC AND IMMUNOMODULATIN G AGENTS
Alkylating Agents	PA16471233 1	161	9.730	2.467	3.65E-05	0.0117	L01A	np	np	L01A	ALKYLATING AGENTS	L01	ANTINEOPLASTIC AGENTS	L.	ANTINEOPLASTIC AND IMMUNOMODULATIN G AGENTS
Protein kinase inhibitors	PA16471320 4	504	30.459	1.740	5.71E-05	0.0117	L01XE	L01XE	protein kinase inhibitors	L01X	OTHER ANTINEOPLASTIC AGENTS	L01	ANTINEOPLASTIC AGENTS	L.	ANTINEOPLASTIC AND IMMUNOMODULATIN G AGENTS
lysine	PA450280	932	56.324	1.527	4.86E-05	0.0117	B05XB03	B05XB	amino acids	B05X	ADDITIVES TO IV SOLUTIONS	B07	BLOOD SUBSTITUTES AND PERFUSION SOLUTIONS	B.	BLOOD AND HEMATOPOIETIC ORGANS
quinine	PA451213	60	3.626	3.585	4.79E-05	0.0117	C05AF01	C05AF	sclerosing agent	C05A	AGENTS FOR TREATMENT OF HEMORRHOIDS AND ANALFISSURES FOR TOPICAL	C05	VASOPROTECTO RS	C.	HEART THERAPY
adenosine	PA448049	604	36.502	1.644	9.70E-05	0.0162	C01EB10	C01EB	Other cardiac drugs	C01E	OTHER HEART PRODUCTS	C01	HEART THERAPY	C.	HEART THERAPY

Module M2

Description	Gene Set	Size	Expect	Ratio	P Value	FDR	ATC5	ATC4	DEFINITION	ATC3	DEFINITION	ATC2	DEFINITION	ATC1	DEFINITION
adenosine	PA16474347	350	17.287	2.256	1.74E-06	5.32E-04	np	np	np	np	np	np	np	np	np
reduced nicotinamide adenine dinucleotide	PA16475508 5	253	12.496	2.401	7.98E-06	1.63E-03	np	np	np	np	np	np	np	np	np
adenosine	PA16617828	155	7.656	2.743	2.51E-05	3.85E-03	np	np	np	np	np	np	np	np	np
cysteine	PA449173	498	24.597	1.830	6.72E-05	8.23E-03	np	np	np	np	np	np	np	np	np
threonine	PA451673	875	43.218	1.550	2.20E-04	0.0193	np	np	np	np	np	np	np	np	np

adenine	PA448048	209	10.323	2.228	2.73E-04	0.0228	np	np	np	np	np	np	np	np	np
hydrogen peroxide	PA449917	157	7.755	2.837	9.42E-06	1.73E-03	A01AB02	A01AB	Anti-infectives and antiseptics for local oral	A01A	STOMATOLOGICS	A01	STOMATOLOGIC S	A.	ALIMENTARY SYSTEM AND METABOLISM
kanamycin	PA450137	116	5.730	2.793	1.83E-04	0.0177	A07AA08	A07AA	antibiotics	A07A	INTESTINAL ANTIINFECTIVES	A07	ANTIDIARRHOICS AND INTESTINAL ANTIPHLOGISTIC	A.	ALIMENTARY SYSTEM AND METABOLISM
netilmicin	PA16475491 3	90	4.445	4.274	6.32E-08	3.87E-05	J01GB07	J01GB	Other aminoglycosid es	J01G	AMINOGLYCOSIDE ANTIBIOTICS	J01	ANTIBIOTICS FOR SYSTEMIC USE	J.	ANTIINFECTIVE FOR SYSTEMIC USE
cefacetrile	PA16477675 2	91	4.495	4.005	3.95E-07	1.45E-04	J01DB10	J01DB	1st generation cephalosporin	J01D	OTHER BETA LACTAM ANTIBIOTICS	J01	ANTIBIOTICS FOR SYSTEMIC	J.	ANTIINFECTIVE FOR SYSTEMIC USE
cefotaxime	PA448852	91	4.495	4.005	3.95E-07	1.45E-04	J01DD01	J01DD	3rd generation cephalosporin	J01D	OTHER BETA LACTAM ANTIBIOTICS	J01	ANTIBIOTICS FOR SYSTEMIC	J.	ANTIINFECTIVE FOR SYSTEMIC USE
ciprofloxacin	PA449009	114	5.631	3.197	1.15E-05	1.93E-03	J01MA02	J01MA	fluoroquinolo nes	J01M	CHINOLONES	J01	ANTIBIOTICS FOR SYSTEMIC	J.	ANTIINFECTIVE FOR SYSTEMIC USE
fluoroquinolones	PA452613	69	3.408	3.815	2.79E-05	3.95E-03	J01MA	J01MA	fluoroquinolo nes	J01M	CHINOLONES	J01	ANTIBIOTICS FOR SYSTEMIC	J.	ANTIINFECTIVE FOR SYSTEMIC USE
Macrolides	PA16471288 2	134	6.619	2.720	1.05E-04	0.0120	J01FA	J01FA	Macrolides	J01F	MACROLID, LINCOSAMIDE AND STREPTOGRAMINE	J01	ANTIBIOTICS FOR SYSTEMIC USE	J.	ANTIINFECTIVE FOR SYSTEMIC USE
tobramycin	PA451704	123	6.075	2.798	1.13E-04	0.0122	J01GB01	J01GB	Other aminoglycosid	J01G	AMINOGLYCOSIDE ANTIBIOTICS	J01	ANTIBIOTICS FOR SYSTEMIC	J.	ANTIINFECTIVE FOR SYSTEMIC USE
Quinolone Antibacterials	PA16471322 9	62	3.062	3.592	2.01E-04	0.0184	J01M	np	0	J01M	CHINOLONES	J01	ANTIBIOTICS FOR SYSTEMIC	J.	ANTIINFECTIVE FOR SYSTEMIC USE
Actinomycines	PA16471231 4	207	10.224	2.641	3.82E-06	8.77E-04	L01DA	L01DA	Actinomycines	L01D	CYTOTOXIC ANTIBIOTICS AND RELATED SUBSTANCES	L01	ANTINEOPLASTIC AGENTS	L.	ANTINEOPLASTIC AND IMMUNOMODULATIN G AGENTS
dactinomycin	PA15191701 2	207	10.224	2.641	3.82E-06	8.77E-04	L01DA01	L01DA	Actinomycines	L01D	CYTOTOXIC ANTIBIOTICS AND RELATED SUBSTANCES	L01	ANTINEOPLASTIC AGENTS	L.	ANTINEOPLASTIC AND IMMUNOMODULATIN G AGENTS
midostaurin	PA16616991 7	162	8.002	2.625	4.89E-05	6.42E-03	L01XE39	L01XE	protein kinase inhibitors	L01X	OTHER ANTINEOPLASTIC AGENTS	L01	ANTINEOPLASTIC AGENTS	L.	ANTINEOPLASTIC AND IMMUNOMODULATIN G AGENTS
cobimetinib	PA16616004 4	32	1.581	5.062	1.25E-04	0.0127	L01XE38	L01XE	protein kinase inhibitors	L01X	OTHER ANTINEOPLASTIC AGENTS	L01	ANTINEOPLASTIC AGENTS	L.	ANTINEOPLASTIC AND IMMUNOMODULATIN G AGENTS

lysine	PA450280	932	46.034	2.129	5.32E-13	9.77E-10	B05XB03	B05XB	amino acids	B05X	ADDITIVES TO IV SOLUTIONS	B07	BLOOD SUBSTITUTES AND PERFUSION SOLUTIONS	B.	BLOOD AND HEMATOPOIETIC ORGANS
hydroquinone	PA449924	78	3.853	3.115	4.17E-04	0.0333	D11AX11	D11AX	Other dermatics	D11A	OTHER DERMATICS	D11	OTHER DERMATICS	D.	DERMATICS
antivirals	PA452174	418	20.646	1.792	4.36E-04	0.0334	D06BB	D06BB	Antivirals	D06B	CHEMOTHERAPEUTICS FOR TOPICAL APPLICATION	D06	ANTIBIOTICS AND CHEMOTHERAPEUTICS FOR DERMATOLOGICAL USE	D.	DERMATICS
adenosine	PA448049	604	29.833	2.145	5.84E-09	5.37E-06	C01EB10	C01EB	Other cardiac drugs	C01E	OTHER HEART PRODUCTS	C01	HEART THERAPY	C.	HEART THERAPY

Module M4

Description	Gene Set	Size	Expect	Ratio	P Value	FDR	ATC5	ATC4	DEFINITION	ATC3	DEFINITION	ATC2	DEFINITION	ATC1	DEFINITION
threonine	PA451673	875	31.613	2.467	8.28E-14	1.52E-10	np	np	np	np	np	np	np	np	np
serine	PA451330	993	35.877	2.146	1.27E-10	1.17E-07	np	np	np	np	np	np	np	np	np
Protein kinase inhibitors	PA164713204	504	18.209	2.636	8.28E-10	5.07E-07	L01XE	L01XE	protein kinase inhibitors	L01X	OTHER ANTINEOPLASTIC AGENTS	L01	ANTINEOPLASTIC AGENTS	L.	ANTINEOPLASTIC AND IMMUNOMODULATING AGENTS
lysine	PA450280	932	33.673	2.109	1.58E-09	7.26E-07	B05XB03	B05XB	amino acids	B05X	ADDITIVES TO IV SOLUTIONS	B07	BLOOD SUBSTITUTES AND PERFUSION SOLUTIONS	B.	BLOOD AND HEMATOPOIETIC ORGANS
proline	PA451125	461	16.656	2.041	6.74E-05	0.0248	np	np	np	np	np	np	np	np	np

Module M5

Description	Gene Set	Size	Expect	Ratio	P Value	FDR	ATC5	ATC4	DEFINITION	ATC3	DEFINITION	ATC2	DEFINITION	ATC1	DEFINITION
prostaglandin E2	PA449345	98	0.685	16.056	7.79E-11	3.58E-08	np	np	np	np	np	np	np	np	np
granulocyte colony-stimulating factor	PA130620649	137	0.958	12.530	1.95E-10	7.17E-08	np	np	np	np	np	np	np	np	np
Oxytocics	PA164713141	96	0.671	14.901	1.24E-09	2.86E-07	np	np	np	np	np	np	np	np	np
Other oxytocics	PA164713113	42	0.294	17.029	1.05E-05	8.06E-04	np	np	np	np	np	np	np	np	np
curcumin	PA151958596	60	0.419	11.921	6.11E-05	3.63E-03	np	np	np	np	np	np	np	np	np
niacin	PA450617	33	0.231	17.339	7.90E-05	4.40E-03	np	np	np	np	np	np	np	np	np

MI-63	PA165290932	66	0.461	10.837	9.68E-05	4.95E-03	np	np	np	np	np	np	np	np	np
polymyxin b sulfate	PA451027	20	0.140	21.457	3.47E-04	0.0133	np	np	np	np	np	np	np	np	np
cilomilast	PA165948031	5	0.035	57.219	4.78E-04	0.0166	np	np	np	np	np	np	np	np	np
epoetin alfa	PA10072	7	0.049	40.870	9.94E-04	0.0257	np	np	np	np	np	np	np	np	np
threonine	PA451673	875	6.117	2.452	1.10E-03	0.0277	np	np	np	np	np	np	np	np	np
prostaglandins	PA452363	140	0.979	10.218	4.90E-08	6.00E-06	A02BB	A02BB	prostaglandins	A02B	MEDICINE FOR PEPTIC ULCUS AND GASTRO-ESOPHAGEAL REFLUX	A02	REMEDY FOR ACID DISEASES	A.	ALIMENTARY SYSTEM AND METABOLISM
Intestinal Antiinflammatory Agents	PA164712843	53	0.371	16.194	1.78E-06	1.82E-04	A07E	np	np	A07E	INTESTINAL ANTIPHLOGISTICS	A07	ANTIDIARRHOICS AND INTESTINAL ANTIPHLOGISTIC	A.	ALIMENTARY SYSTEM AND METABOLISM
dexamethasone	PA449247	122	0.853	9.380	2.18E-06	2.05E-04	A01AC02	A01AC	Corticosteroids for oral topical	A01A	STOMATOLOGICS	A01	STOMATOLOGICS	A.	ALIMENTARY SYSTEM AND METABOLISM
rosiglitazone	PA451283	54	0.378	13.245	3.66E-05	2.24E-03	A10BG02	A10BG	thiazolidinediones	A10B	ANTIDIABETICS, EXCL. INSULINE	A10	ANTIDIABETICS	A.	ALIMENTARY SYSTEM AND METABOLISM
clarithromycin	PA449028	18	0.126	31.788	6.41E-06	5.35E-04	J01FA09	J01FA	Macrolides	J01F	MACROLID, LINCOSAMIDE AND STREPTOGRAMINE	J01	ANTIBIOTICS FOR SYSTEMIC USE	J.	ANTIINFECTIVE FOR SYSTEMIC USE
Other viral vaccines	PA164713135	102	0.713	9.817	7.17E-06	5.73E-04	J07BX	J07BX	Other viral vaccines	J07B	VIRAL VACCINES	J07	Vaccines	J.	ANTIINFECTIVE FOR SYSTEMIC USE
Viral Vaccines	PA164713390	147	1.028	6.812	7.64E-05	4.39E-03	J07B	np	np	J07B	VIRAL VACCINES	J07	Vaccines	J.	ANTIINFECTIVE FOR SYSTEMIC USE
amoxicillin	PA448406	13	0.091	33.011	9.03E-05	4.88E-03	J01CA04	J01CA	Penicillins with an extended spectrum of	J01C	BETALACTAM ANTIBIOTICS, PENICILLIN	J01	ANTIBIOTICS FOR SYSTEMIC USE	J.	ANTIINFECTIVE FOR SYSTEMIC USE
Other Vaccines	PA164712997	106	0.741	6.748	8.80E-04	0.0241	J07X	np	np	J07X	OTHER VACCINES	J07	Vaccines	J.	ANTIINFECTIVE FOR SYSTEMIC USE
Interleukins	PA164712839	351	2.454	12.634	0	0	L03AC	L03AC	interleukins	L03A	IMMUNE STIMULANTS	L03	IMMUNE STIMULANTS	L.	ANTINEOPLASTIC AND IMMUNOMODULATING AGENTS
Interleukin inhibitors	PA164712838	260	1.818	15.405	0	0	L04AC	L04AC	Interleukin Inhibitors	L04A	IMMUNSUPPRESSIVA	L04	IMMUNSUPPRESSIVA	L.	ANTINEOPLASTIC AND IMMUNOMODULATING AGENTS
Tumor necrosis factor alpha (TNF-alpha) inhibitors	PA164713366	177	1.237	21.013	0	0	L04AB	L04AB	Tumor Necrosis Factor alpha (TNF-alpha)	L04A	IMMUNSUPPRESSIVA	L04	IMMUNSUPPRESSIVA	L.	ANTINEOPLASTIC AND IMMUNOMODULATING AGENTS

anakinra	PA10799	39	0.273	25.675	8.56E-09	1.75E-06	L04AC03	L04AC	Interleukin Inhibitors	L04A	IMMUNSUPPRESSIVA	L04	IMMUNSUPPRES SIVA	L.	ANTINEOPLASTIC AND IMMUNOMODULATIN G AGENTS
Immunostimulants	PA16471281 9	236	1.650	7.880	1.01E-08	1.85E-06	L03A	np	np	L03A	IMMUNE STIMULANTS	L03	IMMUNE STIMULANTS	L.	ANTINEOPLASTIC AND IMMUNOMODULATIN G AGENTS
Protein kinase inhibitors	PA16471320 4	504	3.523	4.825	7.21E-08	8.29E-06	L01XE	L01XE	protein kinase inhibitors	L01X	OTHER ANTINEOPLASTIC AGENTS	L01	ANTINEOPLASTIC AGENTS	L.	ANTINEOPLASTIC AND IMMUNOMODULATIN G AGENTS
colony stimulating factors	PA10420	148	1.035	8.699	9.28E-07	1.00E-04	L03AA	L03AA	Colony Stimulating Factors	L03A	IMMUNE STIMULANTS	L03	IMMUNE STIMULANTS	L.	ANTINEOPLASTIC AND IMMUNOMODULATIN G AGENTS
interferons	PA451999	342	2.391	4.183	1.40E-04	6.44E-03	L03AB	L03AB	interferons	L03A	IMMUNE STIMULANTS	L03	IMMUNE STIMULANTS	L.	ANTINEOPLASTIC AND IMMUNOMODULATIN G AGENTS
antineoplastic agents	PA452621	492	3.439	3.489	1.65E-04	7.40E-03	L01	np	np	np	np	L01	ANTINEOPLASTIC AGENTS	L.	ANTINEOPLASTIC AND IMMUNOMODULATIN G AGENTS
canakinumab	PA16595835 8	6	0.042	47.682	7.13E-04	0.0205	L04AC08	L04AC	Interleukin Inhibitors	L04A	IMMUNSUPPRESSIVA	L04	IMMUNSUPPRES SIVA	L.	ANTINEOPLASTIC AND IMMUNOMODULATIN G AGENTS
Blood And Related Products	PA16471256 7	68	0.475	10.518	1.12E-04	0.0054	B05A	np	np	B05A	BLOOD AND RELATED PRODUCTS	B05	BLOOD SUBSTITUTES AND PERFUSION SOLUTIONS	B.	BLOOD AND HEMATOPOIETIC ORGANS
carbohydrates	PA448797	790	5.523	2.897	1.15E-04	5.43E-03	B05BA03	B05BA	solutions for parenteral nutrition	B05B	IV SOLUTIONS	B06	BLOOD SUBSTITUTES AND PERFUSION SOLUTIONS	B.	BLOOD AND HEMATOPOIETIC ORGANS
collagenase	PA449107	138	0.965	10.366	4.27E-08	6.00E-06	D03BA02	D03BA	Proteolytic Enzymes	D03B	ENZYMES	D03	PREPARATIONS FOR THE TREATMENT OF WOUNDS AND Ulcers	D.	DERMATICS
zinc oxide	PA451965	31	0.217	23.072	2.23E-06	2.05E-04	D02AB01	D02AB	Zinc-containing agents	D02A	EMOLLIENTIA AND SKIN PROTECTION PRODUCTS	D02	EMOLLIENTIA AND SKIN PROTECTION PRODUCTS	D.	DERMATICS
erythromycin	PA449493	27	0.189	21.192	3.50E-05	2.22E-03	D10AF02	D10AF	anti-infectives for the treatment of acne	D10A	ACNEA FOR TOPICAL APPLICATION	D10	ACNEMICANTS	D.	DERMATICS

Phenol and derivatives	PA164713161	146	1.021	5.879	5.57E-04	0.0183	D08AE	D08AE	phenol and derivatives	D08A	ANTISEPTICS AND DISINFECTANTS	D08	ANTISEPTICS AND DISINFECTANTS	D.	DERMATICS
antivirals	PA452174	418	2.922	3.422	6.88E-04	0.0205	D06BB	D06BB	Antivirals	D06B	CHEMOTHERAPEUTICS FOR TOPICAL APPLICATION	D06	ANTIBIOTICS AND CHEMOTHERAPEUTICS FOR DERMATOLOGICAL USE	D.	DERMATICS
Thiazides, combinations with psycholeptics and/or analgesics	PA164713346	269	1.881	6.913	4.75E-08	6.00E-06	C03AH	C03AH	Thiazides, combinations with psycholeptics and/or analgesics	C03A	LOW-CEILING DIURETICS, THIAZIDE	C03	DIURETICS	C.	HEART THERAPY
indomethacin	PA449982	51	0.357	11.219	4.38E-04	0.0161	C01EB03	C01EB	Other cardiac drugs	C01E	OTHER HEART PRODUCTS	C01	HEART THERAPY	C.	HEART THERAPY
Antiinflammatory And Antirheumatic Products	PA164712461	42	0.294	27.247	4.24E-10	1.11E-07	M01	np	np	np	np	M01	ANTIPHLOGISTICS AND ANTIRHEUMATICS	M.	MUSCULO-SKELETAL SYSTEM
Specific Antirheumatic Agents	PA164713273	92	0.643	10.884	3.60E-06	3.15E-04	M01C	np	np	M01C	SPECIFIC ANTIRHEUMATIC	M01	ANTIPHLOGISTICS AND ANTIRHEUMATICS	M.	MUSCULO-SKELETAL SYSTEM
Other specific antirheumatic agents	PA164713127	48	0.336	14.901	2.05E-05	1.39E-03	M01CX	M01CX	Other specific anti-inflammatory drugs	M01C	SPECIFIC ANTIRHEUMATIC	M01	ANTIPHLOGISTICS AND ANTIRHEUMATICS	M.	MUSCULO-SKELETAL SYSTEM
Analgesics	PA16471236	269	1.881	6.913	4.75E-08	6.00E-06	N02	np	np	np	np	N02	Analgesics	N	NERVOUS SYSTEM
Respiratory System	PA16471323	348	2.433	6.166	1.88E-08	3.15E-06	R	np	np	np	np	np	np	R.	RESPIRATION TRACT
nitric oxide	PA450635	154	1.077	6.502	1.02E-04	5.09E-03	R07AX01	R07AX	Other means for the respiratory tract	R07A	OTHER RESPIRATION MEANS	R07	OTHER RESPIRATION MEANS	R.	RESPIRATION TRACT
Antiinflammatory	PA16471245	214	1.496	9.358	2.76E-10	8.45E-08	S01B	np	np	S01B	ANTIPHLOGISTICS	S01	Ophthalmics	S.	SENSORY ORGANS
Antiinfectives	PA16471244	529	3.698	3.786	1.86E-05	1.31E-03	S01A	np	np	S01A	ANTIINFECTIVE	S01	Ophthalmics	S.	SENSORY ORGANS
Antiinflammatory agents, non-steroids	PA164712462	128	0.895	7.823	3.16E-05	2.08E-03	S01BC	S01BC	Non-Steroidal Anti-Inflammatory Drugs	S01B	ANTIPHLOGISTICS	S01	Ophthalmics	S.	SENSORY ORGANS

glucocorticoids	PA452347	153	1.070	7.480	1.17E-05	8.62E-04	H02AB	H02AB	Glucocorticoids	H02A	CORTICOSTEROIDS FOR SYSTEMIC USE, PURE	H02	CORTICOSTEROIDS FOR SYSTEMIC USE	H	SYSTEMIC HORMONE PREPARATIONS, EXCL. SEX HORMONES AND INSULINS
oxygen	PA450744	327	2.286	4.375	9.70E-05	4.95E-03	V03AN01	V03AN	Medical gases	V03A	ALL OTHER THERAPEUTIC MEANS	V03A	ALL OTHER THERAPEUTIC MEANS	V.	VARIOUS

Module M6

Description	Gene Set	Size	Expect	Ratio	P Value	FDR	ATC5	ATC4	DEFINITION	ATC3	DEFINITION	ATC2	DEFINITION	ATC1	DEFINITION
tobramycin	PA451704	123	1.101	8.175	1.57E-06	2.89E-03	J01GB01	J01GB	Other aminoglycosid	J01G	AMINOGLYCOSIDE ANTIBIOTICS	J01	ANTIBIOTICS FOR SYSTEMIC	J.	ANTIINFECTIVE FOR SYSTEMIC USE

Module M7

Description	Gene Set	Size	Expect	Ratio	P Value	FDR	ATC5	ATC4	DEFINITION	ATC3	DEFINITION	ATC2	DEFINITION	ATC1	DEFINITION
lysine	PA450280	932	7.063	3.256	4.21E-07	7.74E-04	B05XB03	B05XB	amino acids	B05X	ADDITIVES TO IV SOLUTIONS	B07	BLOOD SUBSTITUTES AND PERFUSION SOLUTIONS	B.	BLOOD AND HEMATOPOIETIC ORGANS

Supplementary Table S8. Summary of the enrichment analysis on druggable genes in each co-expression module.

Module	A. ALIMENTARY SYSTEM AND METABOLISM	B. BLOOD AND HEMATOPOIETIC ORGANS	C. HEART THERAPY	D. DERMATICS	[illegible] HORMONE PREPARATIONS, EXCL. SEX HORMONES AND INSULINS	J. ANTIINFECTIVE FOR SYSTEMIC USE	L. ANTINEOPLASTIC AND IMMUNOMODULATING AGENTS	M. MUSCULO-SKELETAL SYSTEM	N. NERVOUS SYSTEM	R.RESPIRATION TRACT	S.SENSORY ORGANS
**M1**	2	1	2	-	-	-	3	-	-	-	-
**M2**	2	1	1	2	-	8	4	-	-	-	-
**M3**	-	-	-	-	-	-	-	-	-	-	-
**M4**	-	1	-	-	-	-	1	-	-	-	-
**M5**	4	2	2	5	1	5	10	3	1	2	3
**M6**	-	-	-	-	-	1	-	-	-	-	-
**M7**	-	1	-	-	-	-	-	-	-	-	-
**TOTAL**	**8**	**6**	**5**	**7**	**1**	**14**	**18**	**3**	**1**	**2**	**3**

Supplementary Table S12. Main results from all SNPs associated with each module eigengene showing a P-value <0.05 and an r2≧0.6 with one of the independent significant SNPs

ANNOVAR = functional variant classification based on position in or outside of a gene; CADD = Combined Annotation-Dependent depletion score, which predict how deleterious the SNP effect is on protein structure/function (higher scores indicate more deleterious, in bold SNPs with CADD > 12.37); RDB = RegulomeDB scores predict likelihood of regulatory functionality (lower scores indicate higher likelihood); minChrState = minimum chromatin state across 127 tissue types (lower scores indicate more open chromatin); commonChrState = most common chromatin state across 127 tissue types.

Module M1

rsID	Chr	Pos	Non_effect_allele	Effect_allele	MAF	Gwas P	Beta	r2	IndSigSNP	nearestGene	dist	func	CADD	RDB	minChrState	commonChrState
rs73170564	7	147930643	T	G	0.05467	6.77E-06	0.0263	0.89	rs73170578	*CNTNAP2*	0	intronic	2.429	6	5	15
rs73170568	7	147932566	A	T	0.05467	1.31E-06	0.0265	0.89	rs73170578	*CNTNAP2*	0	intronic	2.698	6	5	15
rs144468175	7	147935180	C	A	0.05467	1.34E-06	0.0261	0.89	rs73170578	*CNTNAP2*	0	intronic	0.238	7	5	15
rs17170907	7	147935662	T	C	0.05467	1.34E-06	0.0261	0.89	rs73170578	*CNTNAP2*	0	intronic	2.386	4	5	15
rs73170573	7	147939037	G	T	0.05467	1.19E-06	0.0264	0.89	rs73170578	*CNTNAP2*	0	intronic	**13.03**	7	2	15
rs73170578	7	147953298	C	G	0.05467	2.17E-07	0.0294	1.00	rs73170578	*CNTNAP2*	0	intronic	0.436	7	5	15
rs75374169	7	147960255	G	A	0.03579	3.83E-04	0.0290	0.60	rs73170578	*CNTNAP2*	0	intronic	2.131	4	1	15
rs62096498	18	32836931	G	C	0.07157	8.69E-07	-0.0275	1.00	rs62096513	*ZNF397:ZSCAN30*	0	intronic	1.386	7	4	5
rs62096513	18	32849093	C	T	0.07157	5.40E-07	-0.0283	1.00	rs62096513	*ZSCAN30*	0	intronic	1.243	5	2	15

Module M3

rsID	Chr	Pos	Non_effect_allele	Effect_allele	MAF	Gwas P	Beta	r2	IndSigSNP	nearestGene	dist	func	CADD	RDB	minChrState	commonChrState
rs75370437	16	20670196	A	T	0.06064	2.56E-07	0.0310	1.00	rs75370437	*ACSM3:ACSM1*	0	intronic	8.055	5	5	15

Module M4

rsID	Chr	Pos	Non_effect_allele	Effect_allele	MAF	Gwas P	Beta	r2	IndSigSNP	nearestGene	dist	func	CADD	RDB	minChrState	commonChrState
rs17497197	2	37162975	T	C	0.2465	4.42E-04	0.0118	0.73	rs72873859	*STRN*	0	intronic	2.696	6	5	15
rs7424096	2	37188140	A	C	0.2714	7.26E-04	0.0108	0.70	rs72873860	*STRN*	0	intronic	5.668	7	1	5
rs3770772	2	37192607	T	C	0.2555	2.02E-05	0.0136	0.76	rs72873861	*STRN*	0	intronic	7.945	NA	1	1
rs10181959	2	37200739	A	T	0.3171	1.16E-04	0.0114	0.86	rs72873862	*HEATR5B*	0	intronic	3.5	4	2	15

rs13383000	2	37202741	G	A	0.3131	7.55E-05	0.0117	0.88	rs72873863	*HEATR5B*	0	intronic	2.017	7	5	5
rs13408302	2	37202824	A	T	0.3131	7.55E-05	0.0117	0.88	rs72873864	*HEATR5B*	0	intronic	3.866	6	5	5
rs13408514	2	37203006	A	C	0.3141	1.82E-05	0.0127	0.88	rs72873865	*HEATR5B*	0	intronic	**13.25**	7	5	5
rs6716891	2	37206683	A	G	0.3151	3.49E-06	0.0135	0.88	rs72873866	*HEATR5B*	0	intronic	1.915	6	4	5
rs111664461	2	37210633	G	T	0.2833	1.40E-05	0.0134	0.97	rs72873867	*HEATR5B*	0	intronic	0.627	7	4	5
rs112553879	2	37210637	G	A	0.2833	1.40E-05	0.0134	0.97	rs72873868	*HEATR5B*	0	intronic	1.055	7	4	5
rs143938690	2	37211275	G	A	0.3082	7.73E-05	0.0119	0.86	rs72873869	*HEATR5B*	0	intronic	7.414	7	4	5
rs2247010	2	37212102	A	G	0.3091	8.17E-05	0.0119	0.85	rs72873870	*HEATR5B*	0	intronic	9.068	7	4	5
rs28429673	2	37212854	C	T	0.3082	8.17E-05	0.0119	0.86	rs72873871	*HEATR5B*	0	intronic	0.36	7	4	5
rs6717174	2	37213932	G	T	0.2913	3.86E-05	0.0126	0.91	rs72873872	*HEATR5B*	0	intronic	0.541	7	4	5
rs58762847	2	37214142	C	T	0.3072	7.73E-05	0.0119	0.86	rs72873873	*HEATR5B*	0	intronic	0.22	7	4	5
rs111911796	2	37214321	G	C	0.2863	1.80E-05	0.0130	0.98	rs72873874	*HEATR5B*	0	intronic	0.095	7	4	4
rs2372444	2	37215079	G	C	0.3121	7.55E-05	0.0117	0.88	rs72873875	*HEATR5B*	0	intronic	2.021	6	4	5
rs56163481	2	37216322	G	A	0.3121	7.55E-05	0.0117	0.88	rs72873876	*HEATR5B*	0	intronic	3.546	6	4	5
rs7562158	2	37217191	C	T	0.2853	1.80E-05	0.0130	0.98	rs72873877	*HEATR5B*	0	intronic	0.616	6	4	5
rs7600245	2	37220614	A	C	0.2813	2.60E-06	0.0145	0.96	rs72873878	*HEATR5B*	0	intronic	2.051	7	4	5
rs12105781	2	37221567	T	C	0.2843	2.44E-06	0.0145	0.94	rs72873879	*HEATR5B*	0	intronic	3.718	5	4	5
rs75219072	2	37225706	C	T	0.2803	2.57E-06	0.0145	0.96	rs72873880	*HEATR5B*	0	intronic	0.073	7	4	5
rs10490656	2	37231708	A	G	0.2386	4.30E-06	0.0149	0.80	rs72873881	*HEATR5B*	0	intronic	0.212	NA	2	4
rs17332671	2	37244124	C	G	0.2843	5.92E-07	0.0154	0.98	rs72873882	*HEATR5B*	0	intronic	0.298	6	4	5
rs72873859	2	37246738	T	G	0.2893	5.65E-07	0.0152	1.00	rs72873883	*HEATR5B*	0	intronic	3.25	6	2	4
rs111805423	2	37248924	G	T	0.2843	7.37E-07	0.0152	0.98	rs72873884	*HEATR5B*	0	intronic	1.938	6	4	5
rs146074994	2	37257004	C	T	0.2406	1.12E-05	0.0141	0.80	rs72873885	*HEATR5B*	0	intronic	1.793	7	4	5
rs17404648	2	37263508	T	C	0.3032	8.03E-06	0.0137	0.85	rs72873886	*HEATR5B*	0	intronic	9.279	5	4	5
rs13010038	2	174740242	G	A	0.3857	1.16E-06	-0.0136	1.00	rs6707596	*RPL5P7*	3048	intergenic	2.951	7	5	15
rs6707596	2	174741213	A	G	0.3847	5.53E-07	-0.0140	1.00	rs6707596	*RPL5P7*	4019	intergenic	0.172	7	5	15

Module M5

rsID	Chr	Pos	Non_effect_allele	Effect_allele	MAF	Gwas P	Beta	r2	IndSigSNP	nearestGene	dist	func	CADD	RDB	minChrState	commonChrState
rs10427253	2	166399158	G	T	0.08449	1.12E-06	0.6743	0.99	rs66506812	*CSRNP3*	0	intronic	2.693	6	5	15
rs10427254	2	166399298	C	A	0.08449	1.12E-06	0.6743	0.99	rs66506813	*CSRNP3*	0	intronic	3.301	7	5	15
rs66506812	2	166401325	G	A	0.08549	3.35E-07	0.6945	1.00	rs66506814	*CSRNP3*	0	intronic	0.919	6	5	15
rs66596372	2	166401538	G	C	0.08449	1.12E-06	0.6743	0.99	rs66506815	*CSRNP3*	0	intronic	7.309	4	5	15
rs1514756	2	166405015	T	C	0.05666	8.89E-05	0.6696	0.63	rs66506816	*CSRNP3*	0	intronic	3.723	5	5	15
rs7118843	11	92804005	G	A	0.4622	8.20E-04	0.2198	0.75	rs10830974	*RPL26P31*	18011	intergenic	6.366	7	5	15

rs10830974	11	92807099	T	C	0.3986	7.21E-07	0.3367	1.00	rs10830975	*RPL26P31*	21105	intergenic	9.392	**2b**	5	15
rs1876602	11	92809205	G	T	0.4622	1.35E-03	0.2113	0.75	rs10830976	*RPL26P31*	23211	intergenic	0.834	NA	5	15
rs16918495	11	92810275	C	T	0.4622	7.99E-04	0.2198	0.75	rs10830977	*RPL26P31*	24281	intergenic	1.286	5	5	15
rs1355065	11	92812499	C	G	0.4622	7.99E-04	0.2198	0.75	rs10830978	*RPL26P31*	26505	intergenic	0.886	6	5	15
rs1508617	11	92812922	A	G	0.4622	7.99E-04	0.2198	0.75	rs10830979	*RPL26P31*	26928	intergenic	**17.17**	6	5	15
rs10765584	11	92815687	T	C	0.4781	8.99E-03	0.1701	0.70	rs10830980	*RPL26P31*	29693	intergenic	1.403	7	5	15
rs1508614	11	92816900	T	C	0.4622	1.16E-03	0.2157	0.75	rs10830981	*RPL26P31*	30906	intergenic	2.24	NA	5	15

Module M6

rsID	Chr	Pos	Non_effect_allele	Effect_allele	MAF	Gwas P	Beta	r2	IndSigSNP	nearestGene	dist	func	CADD	RDB	minChrState	commonChrState
rs73866245	3	142648098	A	G	0.02982	1.35E-07	-0.0467	1.00	rs73866245	*RP11-372E1.4*	0	ncRNA_intronic	4.705	4	2	15
rs78403878	3	142648333	G	C	0.02286	6.18E-05	-0.0390	0.77	rs73866245	*RP11-372E1.4*	0	ncRNA_intronic	0.533	4	5	15
rs73866246	3	142651212	C	T	0.02187	1.19E-04	-0.0376	0.74	rs73866245	*RP11-372E1.4*	0	ncRNA_intronic	2.226	6	5	15
rs61397501	3	142651233	A	G	0.02187	7.25E-04	-0.0316	0.74	rs73866245	*RP11-372E1.4*	0	ncRNA_intronic	3.23	6	5	15
rs58601662	3	142651708	G	A	0.02187	7.25E-04	-0.0316	0.74	rs73866245	*RP11-372E1.4*	0	ncRNA_intronic	6.594	6	5	15
rs56183917	3	142652079	G	T	0.02187	1.19E-04	-0.0376	0.74	rs73866245	*RP11-372E1.4*	0	ncRNA_exonic	0.51	5	5	15
rs55930333	3	142652316	G	A	0.02187	7.25E-04	-0.0316	0.74	rs73866245	*RP11-372E1.4*	0	ncRNA_intronic	5.232	5	5	15
rs55756915	3	142652447	G	A	0.02187	1.19E-04	-0.0376	0.74	rs73866245	*RP11-372E1.4*	0	ncRNA_intronic	3.015	5	5	15
rs55785564	3	142652485	G	A	0.02187	1.19E-04	-0.0376	0.74	rs73866245	*RP11-372E1.4*	0	ncRNA_intronic	5.914	5	5	15
rs59026104	3	142652625	C	T	0.02187	1.19E-04	-0.0376	0.74	rs73866245	*RP11-372E1.4*	0	ncRNA_intronic	0.697	7	5	15
rs36098630	3	142659370	T	A	0.02087	1.42E-04	-0.0373	0.71	rs73866245	*RP11-372E1.4*	0	ncRNA_intronic	0.197	**2b**	2	15
rs12636079	3	142659599	C	T	0.02087	1.42E-04	-0.0373	0.71	rs73866245	*RP11-372E1.4*	0	ncRNA_intronic	7.904	4	7	15

rs73866265	3	142661046	C	A	0.02087	1.45E-04	-0.0373	0.71	rs73866245	*RP11-372E1.4*	0	ncRNA_exonic	0.464	5	2	15
rs12629721	3	142661576	T	G	0.02087	1.45E-04	-0.0373	0.71	rs73866245	*RP11-372E1.4*	199	downstream	7.759	5	5	15
rs73866266	3	142662946	G	A	0.02087	1.45E-04	-0.0373	0.71	rs73866245	*RP11-372E1.4*	1569	intergenic	1.888	**2b**	5	14
rs73866268	3	142663465	T	C	0.02087	1.45E-04	-0.0373	0.71	rs73866245	*RP11-372E1.4*	2088	intergenic	1.677	4	1	14
rs12629187	3	142664569	G	A	0.02087	1.45E-04	-0.0373	0.71	rs73866245	*RP11-372E1.4*	3192	intergenic	0.016	3a	5	14
rs59928606	3	142667110	T	A	0.02087	1.45E-04	-0.0373	0.71	rs73866245	*PAQR9*	895	downstream	0.054	**2b**	5	14
rs114943986	5	119664374	T	C	0.03678	1.37E-07	-0.0382	1.00	rs114943986	*CTC-552D5.1*	0	ncRNA_intronic	4.134	7	5	15
rs2462337	7	101440156	C	T	0.2535	3.52E-07	0.0159	1.00	rs2462337	*CUX1*	18802	intergenic	0.268	NA	1	15
rs2410893	7	101441346	G	A	0.2535	3.52E-07	0.0159	1.00	rs2462338	*CUX1*	17612	intergenic	4.676	5	5	15
rs2690913	7	101445313	T	A	0.2535	7.00E-07	0.0157	1.00	rs2462339	*CUX1*	13645	intergenic	2.884	5	5	14
rs72806699	16	84521397	C	G	0.006958	6.39E-07	-0.0717	1.00	rs72806699	*TLDC1*	0	intronic	0.748	7	4	4

Module M7

rsID	Chr	Pos	Non_effect_allele	Effect_allele	MAF	Gwas P	Beta	r2	IndSigSNP	nearestGene	dist	func	CADD	RDB	minChrState	commonChrState
rs9544534	13	36094716	G	A	0.168	2.20E-06	0.0177	0.91	rs12583109	*NBEA*	0	intronic	1.612	7	5	15
rs12583109	13	36096890	C	G	0.1789	6.00E-07	0.0183	1.00	rs12583109	*NBEA*	0	intronic	5.526	6	5	15
rs35056029	13	36096909	A	C	0.1789	6.27E-07	0.0182	1.00	rs12583109	*NBEA*	0	intronic	5.72	7	5	15
rs9530670	13	36099633	G	A	0.1799	2.18E-05	0.0157	0.87	rs12583109	*NBEA*	0	intronic	0.27	6	5	15
rs9544548	13	36099721	A	C	0.1829	4.70E-06	0.0167	0.89	rs12583109	*NBEA*	0	intronic	0.202	6	5	15
rs9574088	13	36101486	G	A	0.1799	2.68E-05	0.0156	0.87	rs12583109	*NBEA*	0	intronic	1.526	7	4	15
rs9565359	13	36104431	T	C	0.1272	4.70E-04	0.0148	0.65	rs12583109	*NBEA*	0	intronic	**18.2**	3a	5	15
rs9565360	13	36104532	G	A	0.17	1.44E-05	0.0165	0.94	rs12583109	*NBEA*	0	intronic	**17.72**	3a	5	15
rs9565361	13	36105341	A	G	0.1272	4.70E-04	0.0148	0.65	rs12583109	*NBEA*	0	intronic	1.43	6	5	15
